# Type I interferon autoantibody footprints reveal neutralizing mechanisms and allow inhibitory decoy design

**DOI:** 10.1084/jem.20242039

**Published:** 2025-03-20

**Authors:** Kevin Groen, Roger Kuratli, Jannik Enkelmann, Sonja Fernbach, Pedro D. Wendel-Garcia, Willy I. Staiger, Marylène Lejeune, Esther Sauras-Colón, Ferran Roche-Campo, Paraskevas Filippidis, Andri Rauch, Swiss HIV Cohort Study, Irene A. Abela, Karoline Aebi-Popp, Alexia Anagnostopoulos, Manuel Battegay, Enos Bernasconi, Dominique Laurent Braun, Heiner C. Bucher, Alexandra Calmy, Matthias Cavassini, Angela Ciuffi, Günter Dollenmaier, Mattias Egger, Luisa Elzi, Jan Fehr, Jacques Fellay, Hansjakob Furrer, Christoph A. Fux, Huldrych Fritz Günthard, Anna Hachfeld, David Haerry, Barbara Hasse, Hans H. Hirsch, Matthias Hoffmann, Irene Hösli, Michael Huber, David Jackson-Perry, Christian R. Kahlert, Laurent Kaiser, Olivia Keiser, Thomas Klimkait, Roger Dimitri Kouyos, Helen Kovari, Katharina Kusejko, Niklaus Labhardt, Karoline Leuzinger, Begogna Martinez de Tejada, Catja Marzolini, Karin Jutta Metzner, Nicolas Müller, Johannes Nemeth, Dunja Nicca, Julia Notter, Paolo Paioni, Giuseppe Pantaleo, Matthieu Perreau, Andri Rauch, Luisa Salazar-Vizcaya, Patrick Schmid, Roberto Speck, Marcel Stöckle, Philip Tarr, Alexandra Trkola, Gilles Wandeler, Maja Weisser, Sabine Yerly, Alexandra Trkola, Huldrych F. Günthard, Roger D. Kouyos, Silvio D. Brugger, Benjamin G. Hale

**Affiliations:** 1 Institute of Medical Virology, University of Zurich, Zurich, Switzerland; 2 Institute of Intensive Care Medicine, University Hospital Zurich, University of Zurich, Zurich, Switzerland; 3 Department of Infectious Diseases and Hospital Epidemiology, University Hospital Zurich, University of Zurich, Zurich, Switzerland; 4 Biobank IISPV-Node Tortosa, Hospital Verge de la Cinta, Institut d’Investigació Sanitària Pere Virgili (IISPV), Tortosa, Spain; 5 Clinical Studies Unit, Hospital Verge de la Cinta, Institut d’Investigació Sanitària Pere Virgili (IISPV), Tortosa, Spain; 6 Intensive Care Unit, Hospital Verge de la Cinta, Institut d’Investigació Sanitària Pere Virgili (IISPV), Tortosa, Spain; 7 Infectious Diseases Service, Department of Medicine, Lausanne University Hospital and University of Lausanne, Lausanne, Switzerland; 8 Department of Infectious Diseases, Inselspital, Bern University Hospital, University of Bern, Bern, Switzerland

## Abstract

Autoantibodies neutralizing type I interferons (IFN-Is; IFNα or IFNω) exacerbate severe viral disease, but specific treatments are unavailable. With footprint profiling, we delineate two dominant IFN-I faces commonly recognized by neutralizing IFN-I autoantibody–containing plasmas from aged individuals with HIV-1 and from individuals with severe COVID-19. These faces overlap with IFN-I regions independently essential for engaging the IFNAR1/IFNAR2 heterodimer, and neutralizing plasmas efficiently block the interaction of IFN-I with both receptor subunits in vitro. In contrast, non-neutralizing autoantibody–containing plasmas limit the interaction of IFN-I with only one receptor subunit and display relatively low IFN-I–binding avidities, thus likely hindering neutralizing function. Iterative engineering of signaling-inert mutant IFN-Is (simIFN-Is) retaining dominant autoantibody targets created potent decoys that prevent IFN-I neutralization by autoantibody-containing plasmas and that restore IFN-I–mediated antiviral activity. Additionally, microparticle-coupled simIFN-Is were effective at depleting IFN-I autoantibodies from plasmas, leaving antiviral antibodies unaffected. Our study reveals mechanisms of action for IFN-I autoantibodies and demonstrates a proof-of-concept strategy to alleviate pathogenic effects.

## Teaser

Autoantibodies neutralizing IFN-Is exacerbate severe viral diseases such as COVID-19. Here, the authors identify IFN-I regions commonly targeted by these autoantibodies, characterize the pathogenic mechanism of autoantibody action, and develop a proof-of-concept inhibitory decoy strategy to alleviate IFN-I autoantibody effects.

## Graphical Abstract

**Figure:**
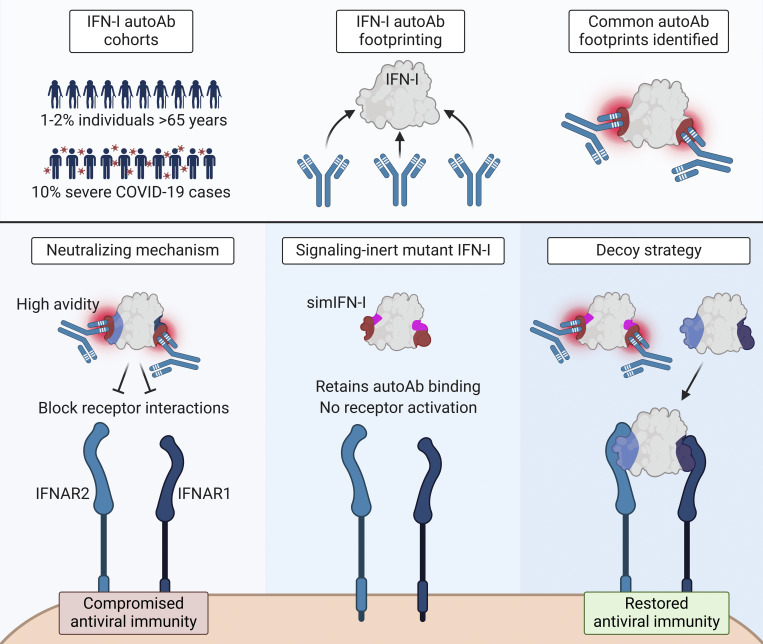

## Introduction

The human type I interferon (IFN-I) system constitutes a crucial component of innate immunity against viral pathogens (McNab et al., 2015). Detection of viral infection by cells triggers a signaling cascade that leads to the production and secretion of soluble IFN-I cytokines (mainly IFNα, IFNβ, and IFNω). IFN-Is then act in autocrine and paracrine manners by binding to a heterodimeric receptor comprised of IFNAR1 and IFNAR2 expressed on the surface of cells (Lazear et al., 2019). This initiates the production of a myriad of IFN-stimulated genes (ISGs) that collectively act to limit virus replication and disease (Lazear et al., 2019). Importantly, several human deficiencies in IFN-I system components are associated with an increased susceptibility of individuals to severe infectious diseases (Crow and Casanova, 2024; Matuozzo et al., 2023; Stertz and Hale, 2021; Zhang et al., 2021, 2022a). Most notable is the recently described association of autoantibodies (autoAbs) targeting IFN-Is with exacerbated viral infections (Abers et al., 2021; Bastard et al., 2021d, 2024a; Casanova et al., 2024; Hale, 2023; Puel et al., 2022; Zhang et al., 2020a), for example COVID-19 caused by SARS-CoV-2 (Bastard et al., 2020, 2022; Casanova and Anderson, 2023; Eto et al., 2022; Koning et al., 2021; Manry et al., 2022). Specifically, ∼10% of critically ill COVID-19 patients and 20% of COVID-19–related deaths are associated with anti-IFN-I autoAbs (Arrestier et al., 2022; Bastard et al., 2020; Chauvineau-Grenier et al., 2022). In addition, anti-IFN-I autoAbs have been found in a significant number of individuals with herpesvirus reactivations (Busnadiego et al., 2022; Pozzetto et al., 1984), in 5% of patients with critical influenza pneumonia (Zhang et al., 2022b), in 20% of patients with critical Middle East respiratory syndrome (MERS) pneumonia (Alotaibi et al., 2023), in 10% of patients with severe tick-borne encephalitis virus (TBEV) disease (Gervais et al., 2024b), in 40% of patients with West Nile virus encephalitis (Barzaghi et al., 2025; Gervais et al., 2023), and in several cases of other rare arboviral diseases (Gervais et al., 2024a). Anti-IFN-I autoAbs also underlie some severe reactions to live-attenuated vaccines (Bastard et al., 2021c). Disease-exacerbating anti-IFN-I autoAbs can neutralize the function of IFN-Is in vitro and are associated with reduced baseline levels of antiviral ISGs (Fernbach et al., 2024), systemic immune alterations (van der Wijst et al., 2021), and compromised nasal immunity against viruses in vivo (Lopez et al., 2021). It is striking that anti-IFN-I autoAbs that neutralize the IFNα or IFNω subtypes have been identified more frequently in disease cohorts than those neutralizing IFNβ (Bastard et al., 2020; Fernbach et al., 2024). Furthermore, children with anti-IFNα autoAbs are more likely to develop COVID-19 pneumonia than those with anti-IFNω autoAbs, indicating that anti-IFNα autoAbs may be the most pathogenic (Bastard et al., 2024b). Currently, little is known about the molecular properties of anti-IFN-I autoAbs, and in particular how these antibodies exert their neutralizing effects.

While some rare individuals are genetically predisposed to develop anti-IFN-I autoAbs early in life (Bodansky et al., 2023; Duncan et al., 2021; Le Voyer et al., 2023; Meager et al., 2006; Meyer et al., 2016; Ramakrishnan et al., 2018; Sjøgren et al., 2022), a proportion of seemingly healthy individuals also appear to spontaneously produce anti-IFN-I autoAbs as they age, and it is estimated that around 2–4% of individuals over the age of 70 years harbor these autoAbs (Bastard et al., 2021a; Fernbach et al., 2024). Once produced, anti-IFN-I autoAbs are maintained lifelong in many individuals and can thus underlie susceptibility to severe viral diseases, such as COVID-19, decades later (Fernbach et al., 2024). Thus, with a sizable proportion of the worldwide elderly population at risk of developing exacerbated viral disease due to anti-IFN-I autoAbs (Bastard et al., 2024a), it is critical that viable and specific treatment options become available. Previously investigated emergency treatment regimens for critically ill COVID-19 patients with anti-IFN-I autoAbs have included plasmapheresis to transiently remove pathogenic autoAbs from circulating blood, as well as treatment of patients harboring anti-IFNα autoAbs with IFNβ to bypass neutralizing autoAbs and thereby restore IFN-I function (Bastard et al., 2021b; de Prost et al., 2021). However, both these strategies have proved suboptimal. For example, in the plasmapheresis trials, multiple rounds of plasmapheresis were required to reduce anti-IFNα autoAb levels in four patients with severe COVID-19 (de Prost et al., 2021), yet this intervention failed to demonstrate improved clinical outcomes. Moreover, plasmapheresis removes all antibodies and other important blood components nonspecifically, which means that not only anti-IFN-I autoAbs are removed by this procedure, but also any protective virus-specific neutralizing antibodies that an individual may have already developed to the infecting agent (Potter et al., 2024). In addition, treatment of anti-IFN-I autoAb–positive individuals with type I IFNs not already targeted by autoAbs may have short-term benefits (Bastard et al., 2021b), but runs the risk of that individual potentially developing new lifelong pathogenic autoAbs against the therapeutic IFN-I, as previously demonstrated (Fernbach et al., 2024). Furthermore, excessive IFN-I signaling can result in uncontrolled inflammation and impairs lung epithelial repair during recovery from viral infections (Davidson et al., 2014; Major et al., 2020), and treatment of COVID-19 patients with IFNβ1a in early trials slightly increased mortality risks in patients requiring treatment with supplemental oxygen (WHO Solidarity Trial Consortium et al., 2021), indicating that delicately balancing the endogenous IFN-I system in vivo is critical to survival.

In this work, we aimed to profile dominant antibody footprints on IFN-Is commonly recognized by anti-IFN-I autoAbs derived from diverse cohorts, with a focus on the more prevalent and pathogenic anti-IFNα autoAbs. We employed this information to understand the precise molecular mechanisms by which neutralizing anti-IFN-I autoAbs block the function of IFN-Is and thereby compromise antiviral immunity. Furthermore, we applied this information to guide the rational engineering of signaling-inert mutant IFN-I (simIFN-I) epitope–containing structures that could have therapeutic potential. In this regard, proof-of-concept experiments demonstrated the efficacy of simIFN-Is to act as decoy proteins to prevent anti-IFN-I autoAbs from neutralizing IFN-I, or to be used as capture proteins to specifically deplete anti-IFN-I autoAbs from plasmas and leave virus-specific neutralizing antibodies unaffected. Development of such tailored strategies has the potential to overcome the pathogenic effects of anti-IFN-I autoAbs by ultimately restoring the body’s own natural IFN-I–mediated antiviral defenses.

## Results

### Identification of IFNα residues targeted by anti-IFNα autoAbs

**Figure 1.**
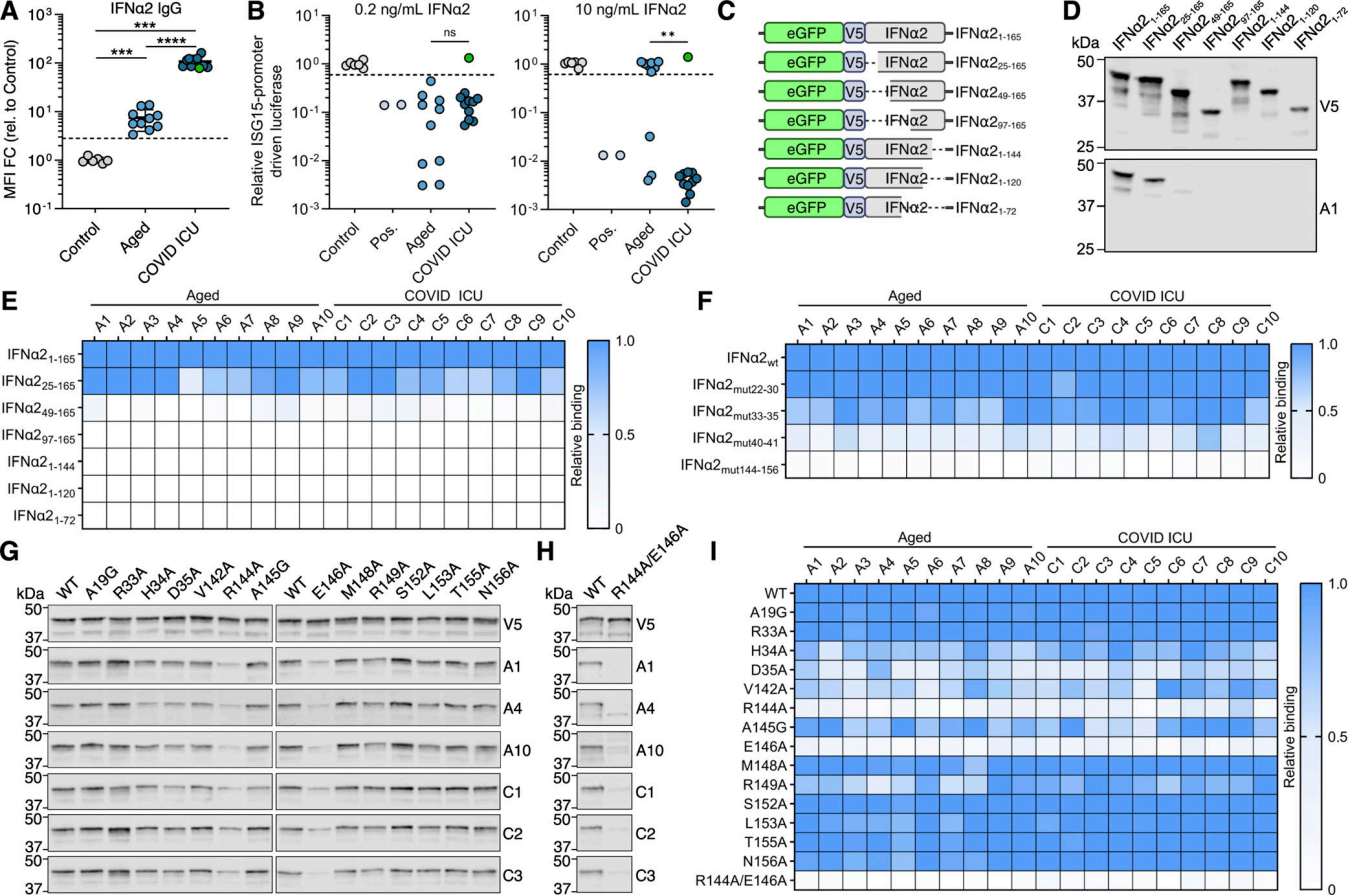
**Identification of IFNα R2 residues targeted by anti-IFNα autoAbs. (A)** Anti-IFNα IgG levels in plasma samples from Aged (*n* = 10) and COVID ICU (*n* = 11) cohorts. Data are expressed as MFI FC values compared with the values of six negative control plasma samples. The dashed line represents the threshold to determine positivity, set as the mean plus 5 standard deviations of the control group. **(B)** Abilities of plasma samples to functionally neutralize IFNα2 at 0.2 or 10 ng/ml. Dashed lines represent neutralization thresholds that were set as the mean minus five standard deviations of the control group. Data are normalized to the luciferase signal from the control group. Pos indicates data from an anti-IFNα mAb–positive control. The green circle (A and B) indicates plasma C11 with non-neutralizing anti-IFNα IgG. **(C)** Schematic overview of eGFP/V5-tagged IFNα2 deletion constructs used for initial screening. **(D)** Western blot reactivity of anti-IFNα IgGs from donor plasma A1 to the IFNα2 deletion constructs shown in C, with anti-V5 IgG used as a loading control. **(E and F)** Western blot quantification of relative anti-IFNα IgG-binding levels from 20 neutralizing plasma samples to the IFNα2 deletion constructs (E) or to the indicated IFNα2 mutant constructs with amino acid stretches substituted for alanines (F). All original blot data are shown in Fig. S1 and Fig. S2. **(G and H)** Western blot reactivity of anti-IFNα IgGs from six plasmas to IFNα2 constructs harboring the indicated single amino acid substitutions (G) or IFNα2_R144A/E146A_ (H), with anti-V5 IgG used as a loading control. **(I)** Quantification of the data in G and H, along with additional plasmas shown in Fig. S3. For all panels, results shown are representative of at least *n* = 2 independent experiments. Statistical significance between groups was determined by the Mann–Whitney U test (A and B): ns, not significant; **P < 0.01; ***P < 0.001; ****P < 0.0001. See also Fig. S1, Fig. S2, and Fig. S3. FC, fold change. Source data are available for this figure: SourceData F1.

**Figure S1.**
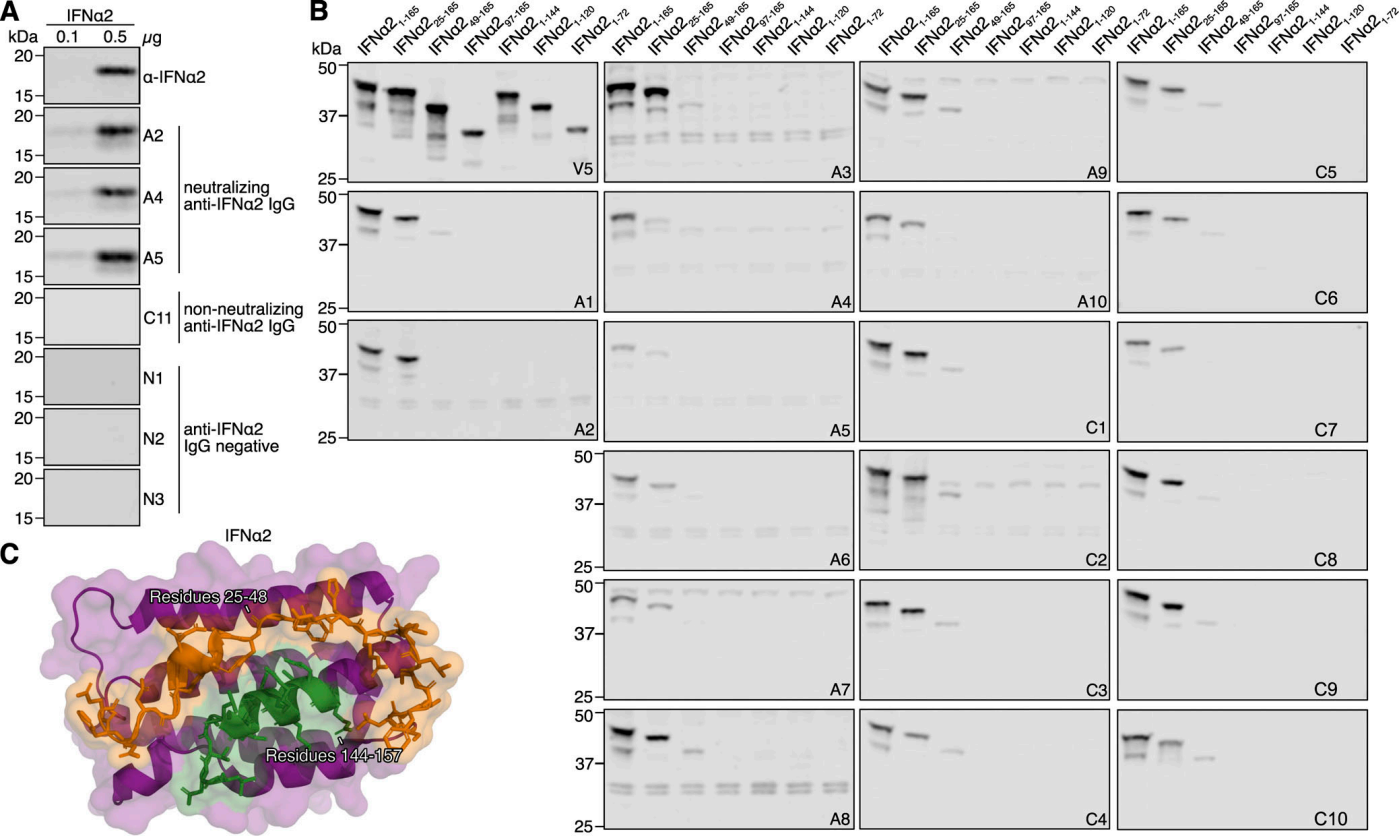
**Western blot reactivity of anti-IFNα IgGs from donor plasmas** **to** **IFNα2 deletion constructs. (A)** Western blot reactivity of the indicated plasma samples positive (A2, A4, A5 from the Aged cohort; C11 from the COVID ICU cohort) or negative (N1–3) for anti-IFNα IgG, or a mouse anti-IFNα2 mAb, to 0.1 or 0.5 µg of recombinant IFNα2. **(B)** Western blot reactivity of anti-IFNα IgGs from 20 neutralizing plasmas (A1–A10 from the Aged cohort, C1–C10 from the COVID ICU cohort) to the IFNα2 deletion constructs shown in Fig. 1 C, with anti-V5 IgG used as a loading control. The V5 and A1 panels are also shown in Fig. 1. **(C)** Previously described structure of the IFNα2 protein (PDB: 3SE3) with identified autoAb-reactive residues 25–48 colored in orange, and identified autoAb-reactive residues 144–157 colored in green. All data shown are representative of at least *n* = 2 similar experiments. Source data are available for this figure: SourceData FS1.

**Figure S2.**
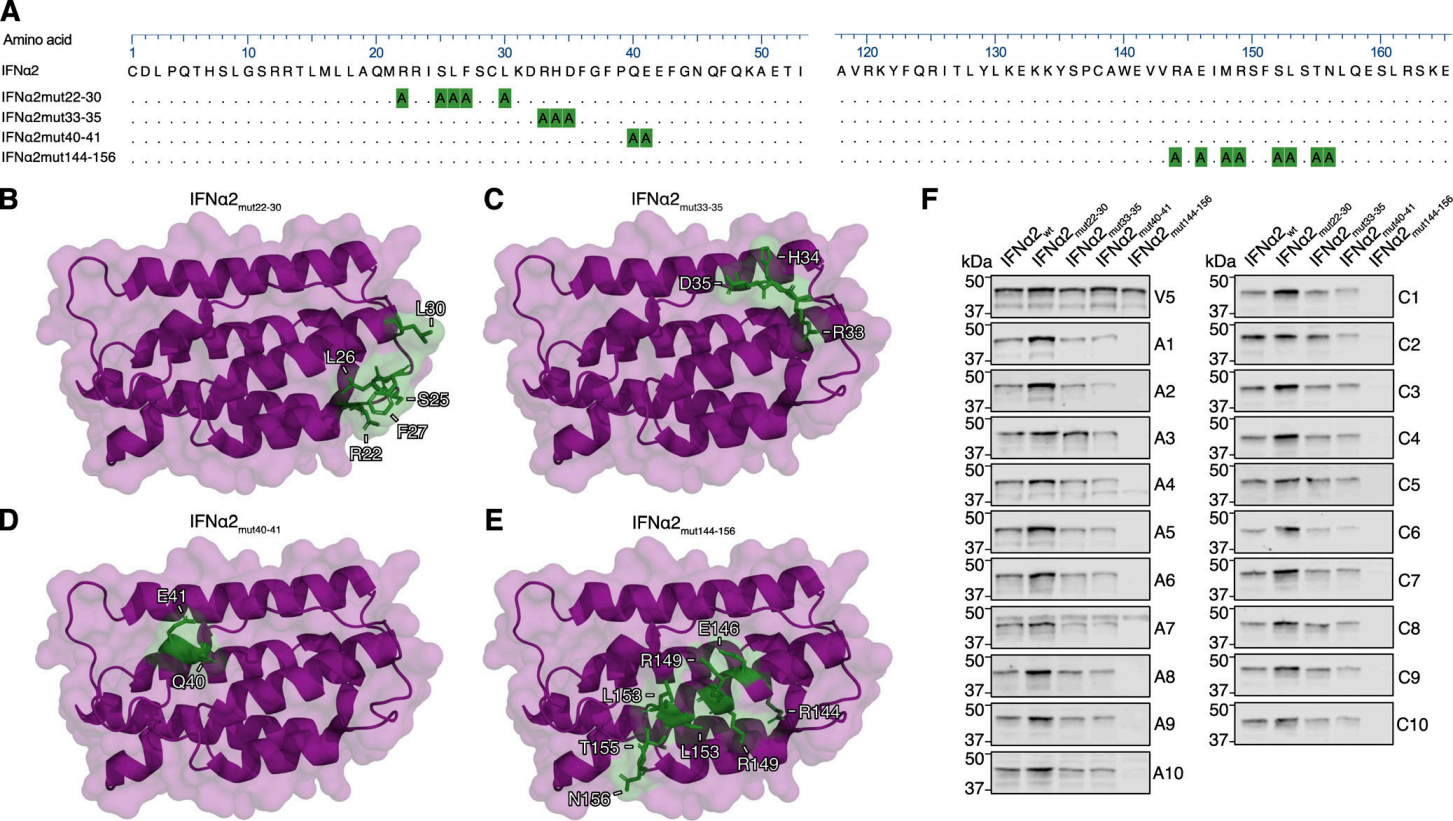
**Western blot reactivity of anti-IFNα IgGs from donor plasmas to IFNα2 mutant constructs with amino acid stretches substituted for alanines. (A)** Amino acid sequence alignment of IFNα2 mutant constructs containing stretches of alanine substitutions. Residues substituted for alanine in each construct are highlighted in green. Amino acid numbering refers to the mature form of IFNα2. **(B–E)** Previously described structures of the IFNα2 protein (PDB: 3SE3) with residues substituted for alanine in each construct colored green. **(F)** Western blot reactivity of anti-IFNα IgGs from 20 neutralizing donor plasmas (A1–A10 from the Aged cohort, C1–C10 from the COVID ICU cohort) to the IFNα2 alanine mutants shown in panels A–E. Anti-V5 IgG was used as a loading control. All data shown are representative of at least *n* = 2 similar experiments. Source data are available for this figure: SourceData FS2.

**Figure S3.**
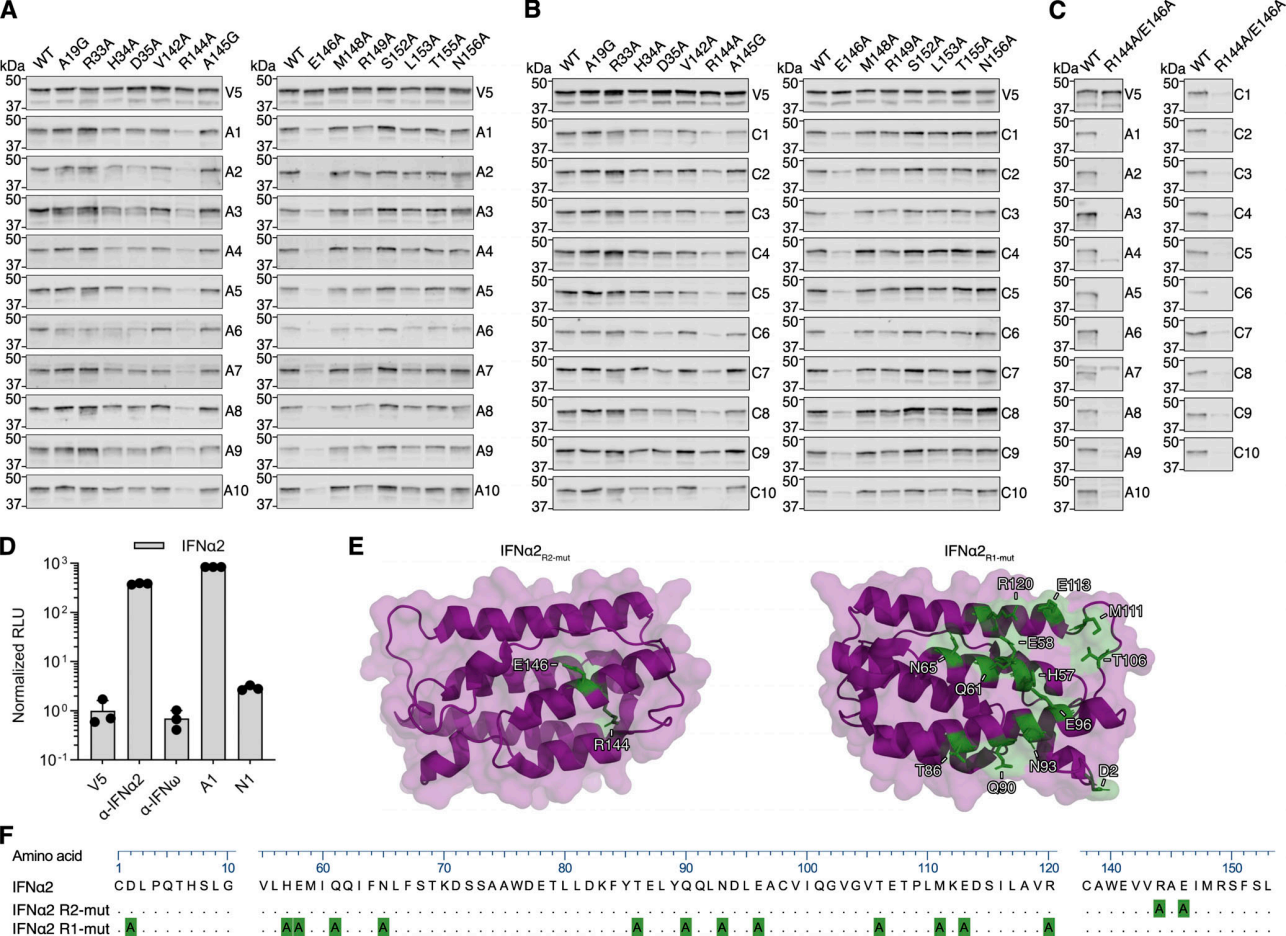
**Fine-mapping of the IFNα2 R2-footprint and generation of an IFNα2 R1 mutant construct. (A–C)** Western blot reactivity of anti-IFNα IgGs from 20 neutralizing plasmas (A1–A10 from the Aged cohort, C1–C10 from the COVID ICU cohort) to IFNα2 constructs harboring single amino acid substitutions (A and B) or IFNα2_R144A/E146A_ (C). Anti-V5 IgG was used as a loading control. The V5, A1, A4, A10, C1, C2, and C3 panels are also shown in Fig. 1. **(D)** Validation of the HiBiT-based qIP assay for the immunoprecipitation of IFNα2 protein using negative control (anti-V5, anti-IFNω) or positive control (anti-IFNα2) mAbs, as well as plasmas A1 (positive) or N1 (negative). Mean values from *n* = 3 replicates are shown, and error bars indicate standard deviations. **(E and F)** Structure of the IFNα2 protein (PDB: 3SE3) with residues substituted for alanine to generate the R1 and R2 mutants colored green (E) or depicted as an amino acid sequence alignment (F), where the numbering refers to the mature form of IFNα2. For all data panels, results are representative of at least *n* = 2 similar experiments. Source data are available for this figure: SourceData FS3.

**Figure 2.**
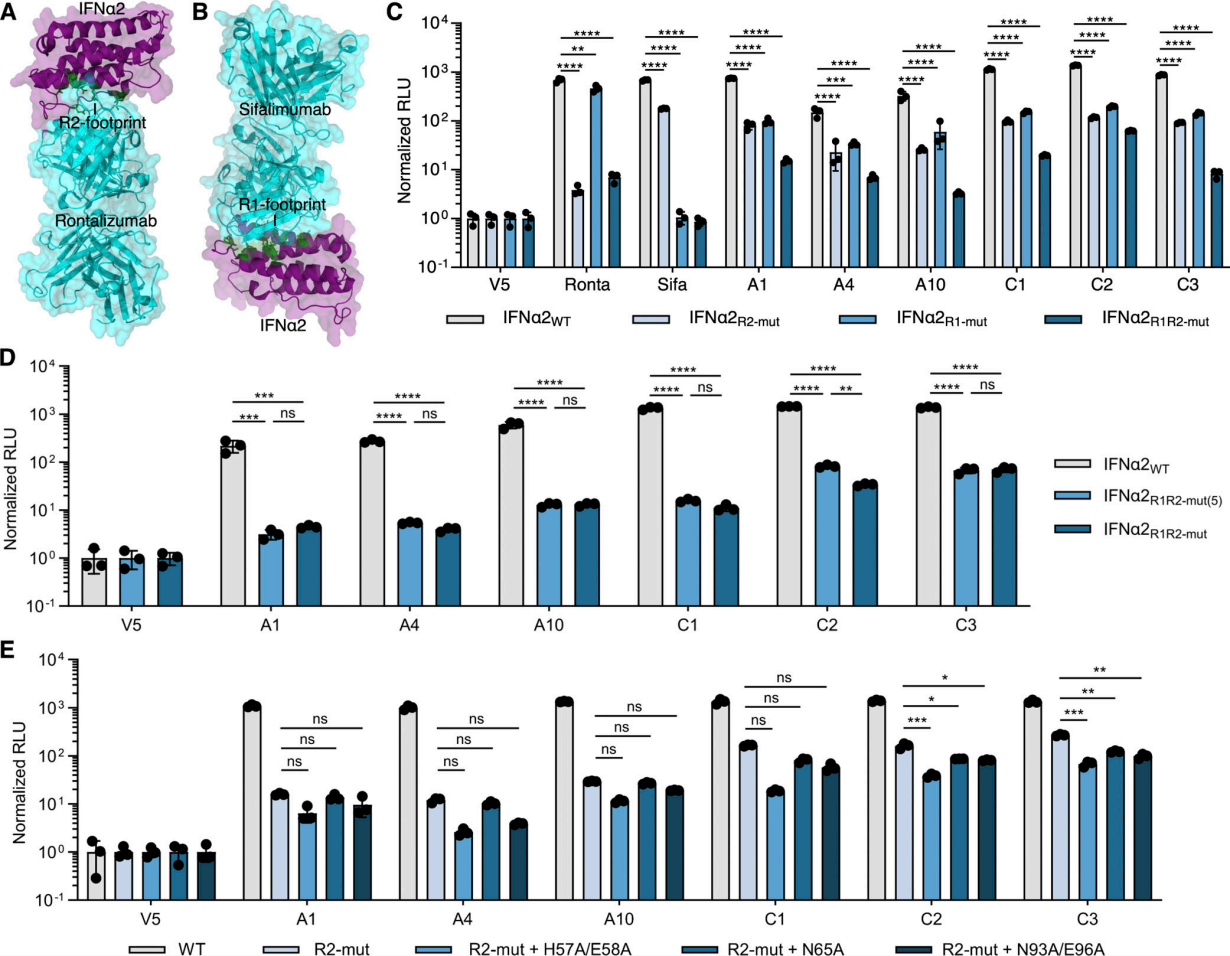
**Identification of IFNα R1 residues targeted by anti-IFNα autoAbs. (A)** Previously determined structure of rontalizumab bound to IFNα2 (PDB: 4Z5R) with the identified R2-footprint recognized by anti-IFNα autoAbs colored in green. **(B)** Previously determined structure of sifalimumab bound to IFNα2 (PDB: 4YPG) with a second footprint, denoted as R1, highlighted in green. **(C)** Ability of Ronta, Sifa, or the indicated plasma samples to immunoprecipitate various HiBiT-tagged IFNα2 mutant proteins. Immunoprecipitation with anti-V5 IgG was used as a normalization control. **(D and E)** Ability of the indicated plasma samples to immunoprecipitate various HiBiT-tagged IFNα2 mutant proteins. Immunoprecipitation with anti-V5 IgG was used as a normalization control. For all data panels, mean values from *n* = 3 replicates are shown. Error bars indicate standard deviations. Results shown are representative of at least *n* = 2 independent experiments. Statistical significance between groups was determined by one-way ANOVA with Dunnett’s multiple comparison correction (C and E) or one-way ANOVA with Tukey’s multiple comparison correction (D): ns, not significant; *P < 0.05; **P < 0.01; ***P < 0.001; ****P < 0.0001. Ronta, rontalizumab; Sifa, sifalimumab.

Previously, we identified neutralizing anti-IFNα autoAbs in the plasmas of ∼10% of individuals from a cohort of patients suffering from severe COVID-19 (“COVID ICU” cohort), as well as in ∼0.85% of individuals over the age of 65 years in a well-treated cohort of people living with HIV-1 (“Aged” cohort) (Busnadiego et al., 2022; Fernbach et al., 2024). Plasma samples from 10 positive individuals from the Aged cohort (termed A1–A10) and 11 positive individuals from the COVID ICU cohort (10 neutralizing and one non-neutralizing, termed C1–C11) were reanalyzed to demonstrate their IFNα-binding IgG levels (Fig. 1 A) and their IFNα-neutralizing potential (Fig. 1 B) as compared to autoAb-negative control donors. Notably, positive plasmas from the COVID ICU cohort contained significantly higher levels of IFNα-binding IgG, and could functionally neutralize higher amounts of IFNα, than positive plasmas from the Aged cohort. To map important IFNα residues engaged by anti-IFNα autoAbs, we first made use of our observation that denatured IFNα could be specifically recognized by plasma-derived neutralizing anti-IFNα IgG in western blot assays (Fig. S1 A), which suggested that at least one important IFNα epitope may be linear in nature. Interestingly, we could not observe recognition of denatured IFNα in western blot assays using the plasma with non-neutralizing anti-IFNα autoAbs (plasma C11) (Fig. S1 A). We therefore initially generated a panel of eGFP/V5-tagged N- and C-terminally truncated IFNα2 proteins that should retain linear epitopes in their denatured states (Fig. 1 C), and assessed reactivity patterns of the polyclonal anti-IFNα IgG from all 20 neutralization-positive plasmas (10 from each cohort). Strikingly, all neutralization-positive plasma samples tested from both cohorts displayed similar differential binding to each of the eGFP/V5-tagged IFNα2 constructs (Fig. 1, D and E; and Fig. S1 B). While removal of the first 24 N-terminal amino acid residues of mature IFNα2 did not generally result in loss of anti-IFNα IgG reactivity for most neutralizing plasmas tested, removal of the first 48 N-terminal amino acid residues led to a major loss of reactivity for all neutralizing plasmas, irrespective of the cohort tested (Fig. 1, D and E; and Fig. S1 B). Furthermore, anti-IFNα IgG reactivity was completely lost for all neutralizing plasmas from both cohorts when the final 20 C-terminal amino acids of IFNα2 were removed (Fig. 1, D and E; and Fig. S1 B). These data indicate that IFNα2 N-terminal residues 25–48, along with C-terminal residues 145–165, contain common binding sites for neutralizing anti-IFNα IgG autoAbs that can be recognized on linear IFNα2 by western blot. From the previously determined structure of IFNα2 (Thomas et al., 2011), it is apparent that these two linear regions are normally adjacent to one another, and thus, the anti-IFNα IgG autoAbs may be partially recognizing each of these regions independently in the western blot assay that otherwise may form a single recognition face comprised of both regions in the three-dimensional structure (Fig. S1 C). To dissect the binding residues further, four additional IFNα2 mutant constructs were generated that each contained multiple alanine substitutions in regions 25–48 and 145–165. The selection of residues for alanine substitution was based on their usual surface accessibility and proximity to one another in the three-dimensional structure of IFNα2 (Fig. S2, A–E). Western blot analysis of neutralizing plasma anti-IFNα IgG reactivity to these four linearized mutant IFNα2 constructs demonstrated that for all 20 neutralizing plasma samples tested across both cohorts, residues in regions 144–156 generally dominated IFNα2 reactivity, while residues in regions 33–35 and 40–41 had a more minor or donor-specific role (Fig. 1 F and Fig. S2 F). Next, 14 IFNα2 constructs harboring single amino acid substitutions were generated to refine the autoAb footprinting. Across the 20 neutralizing plasma samples tested, the most striking loss of anti-IFNα IgG western blot reactivity was observed against constructs IFNα2_R144A_ and IFNα2_E146A_ (Fig. 1 G and Fig. S3, A and B). Furthermore, a double-substitution construct harboring alanine substitutions at both R144 and E146, IFNα2_R144A/E146A_, completely lost all western blot recognition by these neutralizing plasmas (Fig. 1, H and I; and Fig. S3 C). Next to these global reactivities, we also observed donor-specific anti-IFNα IgG autoAb reactivities to mutant IFNα2 constructs harboring substitutions at H34, D35, V142, A145, and R149. Together, these data demonstrate that neutralizing anti-IFNα IgG autoAbs from 20 individuals across two independent cohorts can recognize shared IFNα2-binding residues that are dominated by R144 and E146. Notably, this region overlaps with the previously determined epitope of rontalizumab (Fig. 2 A), a therapeutic antibody that exerts its neutralizing function by blocking the interaction between IFNα and IFNAR2 (Maurer et al., 2015). From here on in, we therefore refer to these autoAb-binding residues as the R2-footprint.

**Figure 3.**
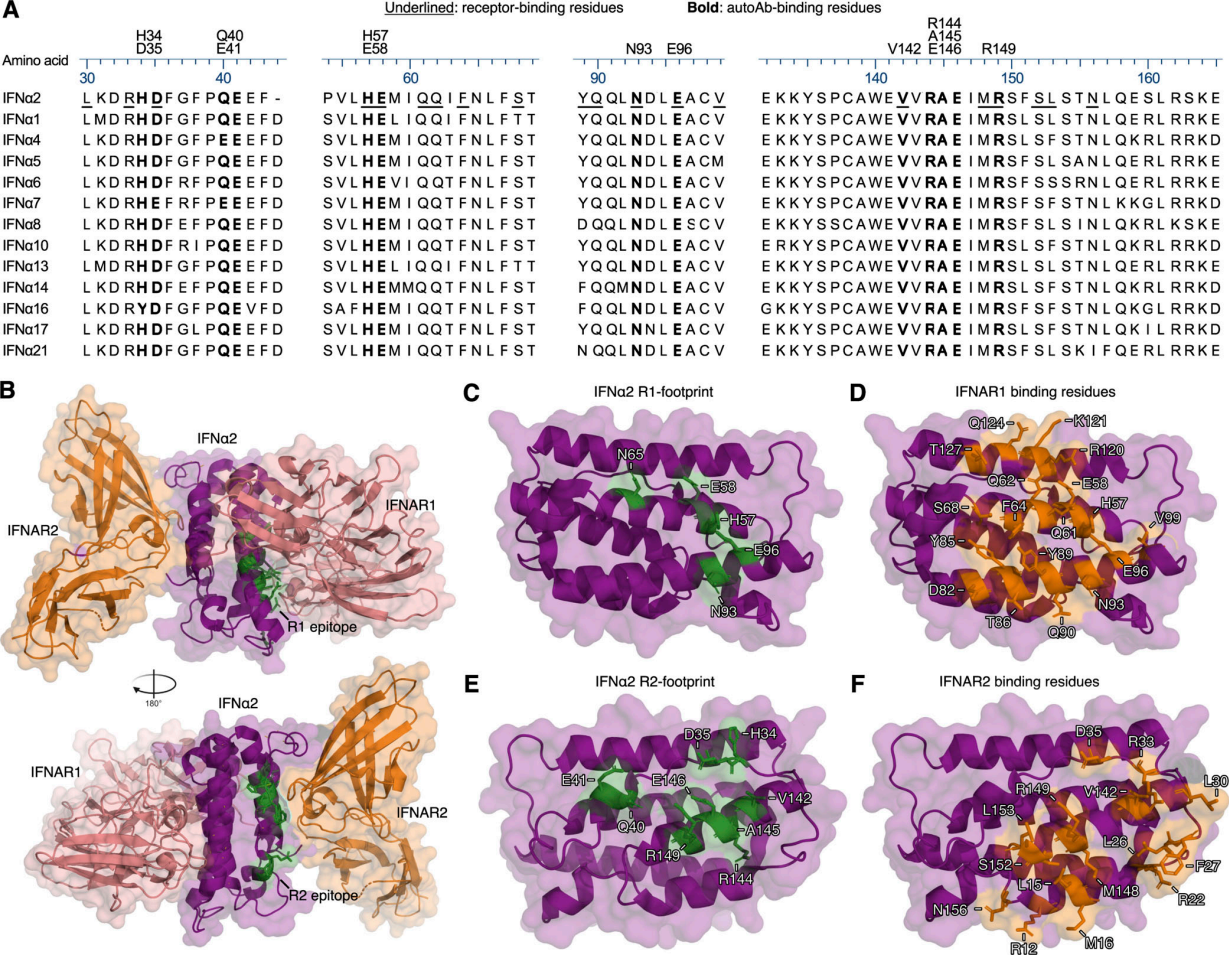
**Comparison of IFNα residues targeted by autoAbs or bound by IFNAR1/IFNAR2. (A)** Amino acid sequence alignment of all human IFNα subtypes. Residues that are part of the R1 or R2 autoAb footprints are highlighted in bold, and receptor-binding residues are underlined. Amino acid numbering refers to the mature form of IFNα2. **(B)** Previously determined structure of the IFNα2-IFNAR1-IFNAR2 protein complex (PDB: 3SE3). The identified R1 and R2 autoAb footprints are colored green. **(C and D)** Side-by-side comparison of the IFNα2 structure with important R1-footprint residues colored green (C) and important IFNAR1-binding residues colored orange (D). **(E and F)** Side-by-side comparison of the IFNα2 structure with important R2-footprint residues colored green (E) and important IFNAR2-binding residues colored orange (F).

Since western blotting typically favors the detection of linearized epitopes, we established a quantitative immunoprecipitation (qIP) assay to assess the presence of additional anti-IFNα IgGs in donor plasmas that may only recognize native, folded IFNα, and not linear epitopes. The assay relies on using donor plasma samples to immunoprecipitate cell-expressed HiBiT-tagged IFNα2 proteins, with quantification of relative immunoprecipitation success subsequently assessed by supplementing the recovered IFNα2-HiBiT with LgBiT and determining reconstituted luciferase activity. As a validation of this assay, mature wild-type IFNα2-HiBiT was readily immunoprecipitated by a commercially available mouse anti-IFNα mAb, but not by commercially available mouse anti-V5 or anti-IFNω mAbs (Fig. S3 D). Furthermore, an anti-IFNα IgG autoAb–positive neutralizing plasma sample (A1) showed strong reactivity to wild-type IFNα2-HiBiT in this assay, while an autoAb-negative plasma sample (N1) exhibited no reactivity, thus demonstrating both assay specificity and sensitivity (Fig. S3 D). Our qIP assay was then used to assess the interaction of six autoAb-positive neutralizing plasma samples (three from the Aged cohort and three from the COVID ICU cohort) with wild-type and mutant IFNα2-HiBiT constructs containing alanine substitutions in either the R2-footprint or an R1-footprint, which we defined as the epitope recognized by sifalimumab and which targets the interface between IFNα and IFNAR1 (Oganesyan et al., 2015) (Fig. 2 B). The IFNα2_R2-mut_ contained substitutions R144A/E146A, while IFNα2_R1-mut_ contained alanine substitutions at 13 amino acids based on the previously determined structural interface with sifalimumab (Oganesyan et al., 2015) (Fig. S3, E and F). An IFNα2_R1R2-mut_ contained the combination of these two mutants. As expected, relative to IFNα2_WT_ qIP, rontalizumab specifically lost most reactivity to IFNα2_R2-mut_-containing constructs, while sifalimumab lost most reactivity to IFNα2_R1-mut_-containing constructs (Fig. 2 C). All six donor-neutralizing plasmas showed a similar, strongly reduced reactivity to both the IFNα2_R1-mut_ and IFNα2_R2-mut_ constructs, and all six plasmas lost most reactivity (95–99%) to the IFNα2_R1R2-mut_ construct (Fig. 2 C). The low residual reactivity of plasmas to IFNα2_R1R2-mut_ (1–5%) likely reflects additional minor unmapped binding residues. We then compared the IFNα2_R1R2-mut_, containing 13–amino acid substitutions in the R1-footprint, with a construct in which the R1-footprint contained only five substitutions (IFNα2_R1R2-mut(5)_, with alanine substitutions at residues H57, E58, N65, N93, and E96). Five out of six neutralizing plasmas lost reactivity to both constructs equally (Fig. 2 D), while plasma C2 lost slightly more reactivity to the mutant with 13 substitutions (Fig. 2 D), indicating expected subtle differences in the R1-footprint between plasmas. To further delineate the R1-footprint, three additional constructs were generated in the IFNα2_R2-mut_ backbone that contained the independent substitutions H57A/E58A, N65A, or N93A/E96A. qIP analysis of these constructs suggested that all these residues together likely compose the core of the R1-footprint bound by neutralizing plasma autoAbs, as each substitution had measurable effects on binding that varied between plasmas (Fig. 2 E). These data using native IFNα2 constructs extend the breadth and specificity of our antibody footprint profiling, and demonstrate that neutralizing plasmas from both Aged and COVID ICU cohorts contain anti-IFNα IgG autoAbs that recognize IFNα2 R1- and R2-footprints similar to the epitopes recognized by rontalizumab and sifalimumab. Importantly, these regions are highly conserved among IFNα subtypes (Fig. 3 A), demonstrating that these autoAbs can likely neutralize all IFNα subtypes.

### Mechanisms of IFNα neutralization by anti-IFNα autoAbs

**Figure 4.**
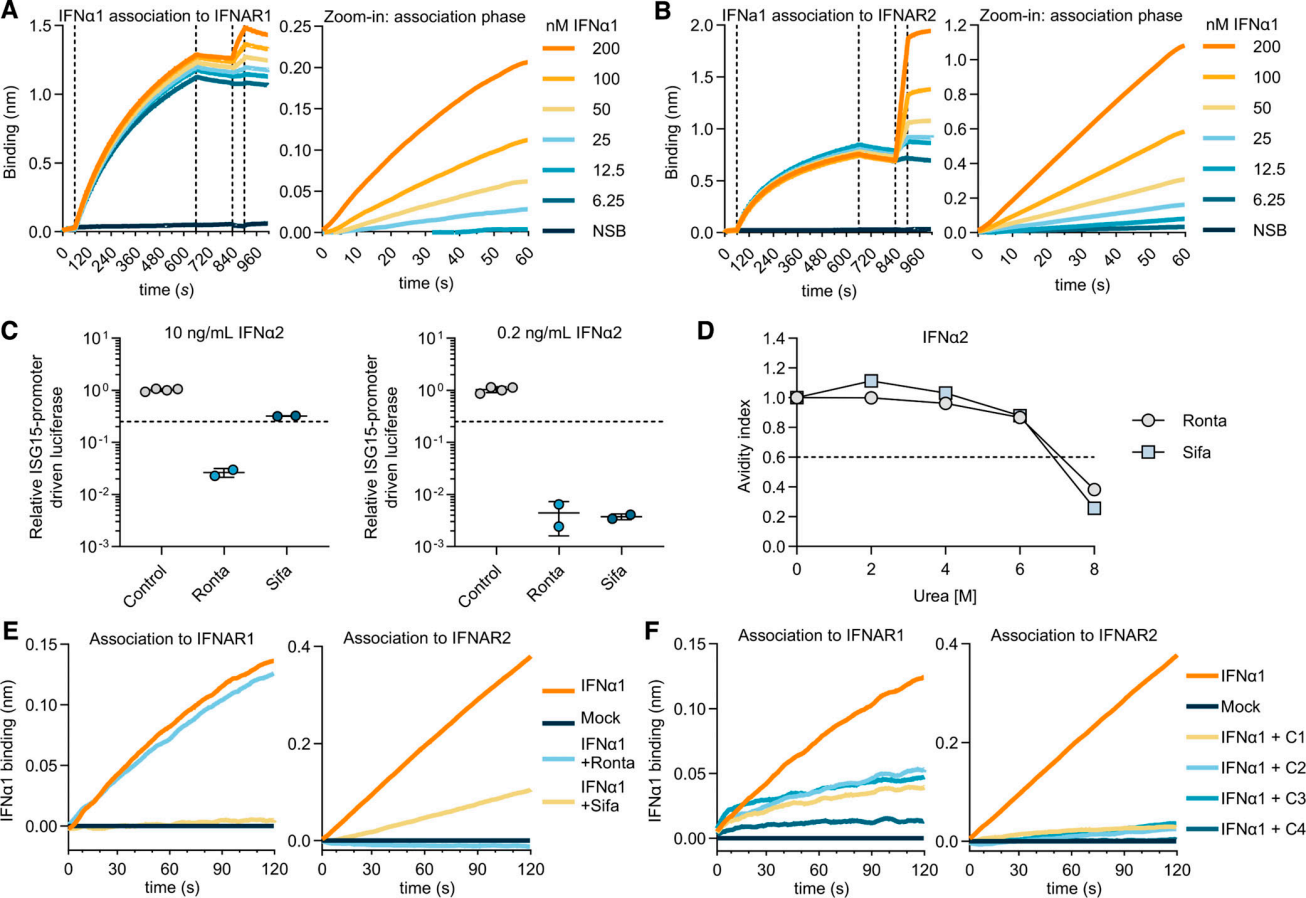
**Mechanism of IFNα neutralization by anti-IFNα autoAbs. (A and B)** Optimization of BLI assays to detect association of IFNα1 with IFNAR1 (A) or IFNAR2 (B). NSB: no receptor loaded, association phase with 200 nM IFNα1. Zoom-in images of the association phases are shown. **(C)** Abilities of rontalizumab and sifalimumab to neutralize IFNα2 at 10 or 0.2 ng/ml. Dashed lines represent neutralization thresholds set at 75% reduction compared with control donor samples. Data are normalized to the luciferase signal from the control group. Mean values and standard deviations are shown and are representative of at least *n* = 2 independent experiments. **(D)** IFNα2 avidity indexes as determined for rontalizumab and sifalimumab at different urea concentrations. The dashed line indicates the threshold for high avidity (>0.6). **(E)** Blocking of the interaction between IFNα1 and IFNAR1 or IFNAR2 by Ronta and Sifa. **(F)** Blocking of the interaction between IFNα1 and IFNAR1 or IFNAR2 by four plasmas containing neutralizing anti-IFNα autoAbs from the COVID ICU cohort. For all data panels, results shown are representative of at least *n* = 2 similar experiments. Ronta, rontalizumab; Sifa, sifalimumab; NSB, nonspecific binding control.

Given our antibody footprinting results, we hypothesized that anti-IFNα autoAb–containing plasmas may commonly exert their neutralizing effects by preventing interactions between IFNα and IFNAR1/IFNAR2. Indeed, such mechanisms seem plausible from the available crystal structures of IFNα2 bound to its heterodimeric receptor (Thomas et al., 2011), as well as a side-by-side comparison of our mapped anti-IFNα autoAb R1- and R2-footprints with the amino acids important for binding of IFNα to each receptor subunit (Fig. 3, B–F). To formally test this, we employed biolayer interferometry (BLI) assays to sensitively measure the interaction of IFNα with each receptor subunit. The lowest concentration of IFNα1 that could be robustly detected as associating with IFNAR1 or IFNAR2 (individually loaded onto Ni-NTA biosensors) was determined as 25 nM (Fig. 4, A and B). We next sought to validate the applicability of BLI assays to detect mechanisms of IFNα neutralization by studying the epitope-specific mAbs rontalizumab and sifalimumab. In our hands, R2-binding rontalizumab was more potent than R1-binding sifalimumab at neutralizing IFNα (Fig. 4 C), but both antibodies exhibited high avidities for IFNα, as defined by experimental IFNα2 avidity indexes >0.6 at 6 M urea (Fig. 4 D) (Bauer et al., 2021; Prince and Lapé-Nixon, 2014). Consequently, preincubation of rontalizumab with IFNα1 specifically blocked the interaction between IFNα1 and IFNAR2, but not between IFNα1 and IFNAR1 (Fig. 4 E) (Maurer et al., 2015), demonstrating that blocking the interaction between IFNα and a single receptor subunit can be sufficient for neutralization. In addition, preincubation of sifalimumab with IFNα1 completely blocked the interaction between IFNα1 and IFNAR1 as expected, but unexpectedly also partially blocked the interaction between IFNα1 and IFNAR2 (Fig. 4 E), presumably via steric hindrance, as the sifalimumab R1-epitope is distal to the IFNAR2-binding site on IFNα2 (Oganesyan et al., 2015). We next assessed the abilities of four neutralizing anti-IFNα autoAb–positive plasmas derived from the COVID ICU cohort to interfere with the interaction between IFNα1 and the receptor subunits. Plasma samples from this cohort were selected for this analysis based on their high anti-IFNα autoAb titers (Fig. 1, A and B) that we found to be required for BLI measurements. All four tested neutralizing plasma samples (C1–C4) were readily able to block the interaction between IFNα1 and IFNAR1, as well as between IFNα1 and IFNAR2 (Fig. 4 F). These data indicate that anti-IFNα autoAb–positive plasmas have the capacity to interfere with IFNα engagement with both IFNAR1 and IFNAR2 receptor subunits.

### AutoAbs neutralizing IFNω act similar to those neutralizing IFNα

**Figure 5.**
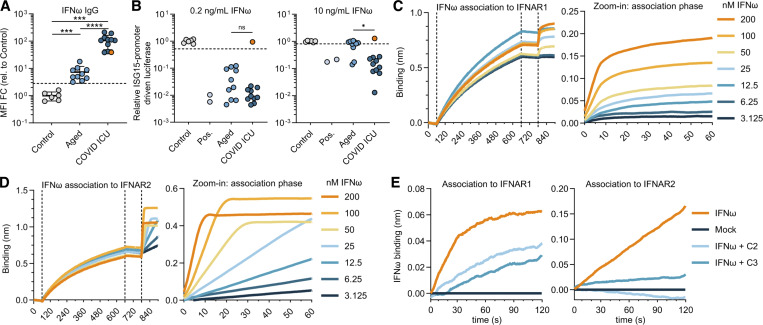
**Mechanism of IFNω neutralization by anti-IFNω autoAbs. (A)** Anti-IFNω IgG levels in plasma samples from Aged (*n* = 10) and COVID ICU (*n* = 11) cohorts. Data are expressed as MFI FC values compared with the values of six negative control plasma samples. The dashed line represents the threshold to determine positivity, set as the mean plus 5 standard deviations of the control group. **(B)** Abilities of plasma samples to functionally neutralize IFNω at 0.2 or 10 ng/ml. Data are normalized to the luciferase signal from the control group. Dashed lines represent neutralization thresholds that were set as the mean minus 5 standard deviations of the control group. Pos indicates data from an anti-IFNω mAb–positive control. The orange circle (A and B) indicates plasma C4 with non-neutralizing anti-IFNω IgG. **(C and D)** Optimization of the concentration of IFNω required for association with IFNAR1 (C) and IFNAR2 (D) in BLI assays. Zoom-in images of the association phases are shown. **(E)** BLI assays to demonstrate inhibition of the interaction between IFNω and IFNAR1 or IFNAR2 by two plasmas harboring neutralizing anti-IFNω IgGs from the COVID ICU cohort. For all data panels, results are representative of at least *n* = 2 independent experiments. Statistical significance between groups was determined by the Mann–Whitney U test (A and B): ns, not significant; *P < 0.05; ***P < 0.001; ****P < 0.0001. FC, fold change.

In addition to autoAbs neutralizing IFNα subtypes, autoAbs that neutralize IFNω are also associated with increased susceptibility to developing severe viral disease (Arrestier et al., 2022; Bastard et al., 2020; Gervais et al., 2023; Hale, 2023; Zhang et al., 2022b). Since plasma samples containing neutralizing anti-IFNω autoAbs, usually together with anti-IFNα autoAbs, were also previously identified in both our COVID ICU and Aged cohorts (Busnadiego et al., 2022; Fernbach et al., 2024), we aimed to assess any similarities in their mechanisms of action to those of anti-IFNα autoAbs. 10 plasma samples from the Aged cohort and 11 from the COVID ICU cohort (10 neutralizing and one non-neutralizing) were selected and reanalyzed to demonstrate their anti-IFNω–binding IgG and neutralizing potential (A3–A12 for the Aged cohort, and C1–C10 plus C12 for the COVID ICU cohort). Similar to what was observed for anti-IFNα autoAbs, the levels of anti-IFNω–binding IgG and neutralizing activities were significantly higher in plasmas from the COVID ICU cohort than in those from the Aged cohort (Fig. 5, A and B). To identify the mechanism of action of neutralizing anti-IFNω autoAbs, BLI assays to measure the interaction between IFNω and both IFNAR1 and IFNAR2 receptor subunits were established and optimized (Fig. 5, C and D). Subsequently, preincubation of IFNω with two high-titer neutralizing anti-IFNω autoAb–positive plasma samples (C2 and C3) resulted in reduced binding of IFNω to both IFNAR1 and IFNAR2 (Fig. 5 E). These results demonstrate that neutralizing plasmas with anti-IFNω autoAbs can block the interaction between IFNω and both IFNAR1 and IFNAR2 receptor subunits, mechanisms similar to that employed by neutralizing anti-IFNα autoAb–containing plasmas. Furthermore, these data suggest that the anti-IFNω autoAb response is also polyclonal, and plasmas contain a mixture of autoAbs targeting IFNω R1- and R2-footprints analogous to those on IFNα.

### Understanding non-neutralizing anti-IFN-I autoAbs

**Figure 6.**
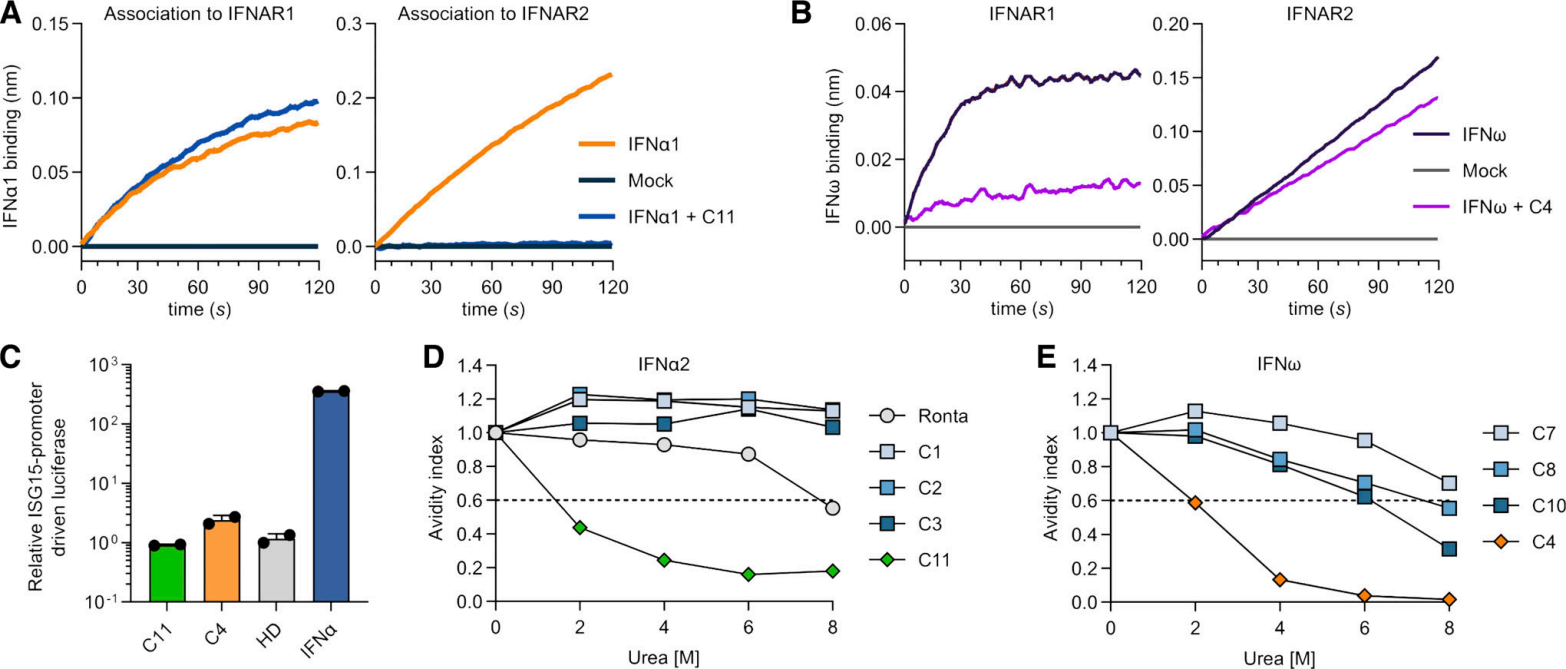
**Non-neutralizing anti-IFN-I autoAbs display relatively low avidities. (A)** BLI assays to assess inhibition of the interaction between IFNα1 and IFNAR1 or IFNAR2 by plasma C11 harboring non-neutralizing anti-IFNα IgGs. **(B)** BLI assays to assess inhibition of the interaction between IFNω and IFNAR1 or IFNAR2 by plasma C4 harboring non-neutralizing anti-IFNω IgGs. **(C)** Immunostimulatory activity of plasmas C11 and C4 compared with a HD plasma and 1,000 U/ml IFNα2. Data are normalized to the luciferase signal from untreated cells. Mean values from *n* = 2 replicates are shown. Error bars indicate standard deviations. **(D)** IFNα2 avidity indexes as determined for Ronta, three plasmas with neutralizing anti-IFNα IgGs (C1–C3), and plasma C11 with non-neutralizing anti-IFNα IgGs at different urea concentrations. **(E)** IFNω avidity indexes as determined for three plasmas with neutralizing anti-IFNω IgGs (C7, C8, C10) and plasma C4 with non-neutralizing anti-IFNω IgGs at different urea concentrations. The dashed lines (D and E) indicate the threshold for high avidity (>0.6). For all data panels, results are representative of at least *n* = 2 similar experiments. Ronta, rontalizumab. HD, healthy donor.

We next aimed to address the mechanism that underlies the functional difference between neutralizing and non-neutralizing anti-IFN-I autoAbs. The COVID ICU cohort contained two plasma samples with high anti-IFN-I–binding IgG, but without any detectable neutralizing activity, even against low IFN-I doses (plasma C11 with non-neutralizing anti-IFNα IgG [Fig. 1, A and B], and plasma C4 with non-neutralizing anti-IFNω IgG [Fig. 5, A and B]). We initially measured the ability of these plasmas to inhibit the interaction between each IFN-I and IFNAR1/IFNAR2. The non-neutralizing plasma C11 limited the interaction between IFNα and IFNAR2, but not between IFNα and IFNAR1 (Fig. 6 A). Conversely, non-neutralizing plasma C4 limited the interaction between IFNω and IFNAR1, but not between IFNω and IFNAR2 (Fig. 6 B). The observation that autoAbs from both non-neutralizing plasmas could only inhibit the interaction between an IFN-I and a single receptor was similar to rontalizumab, albeit rontalizumab has potent neutralizing capabilities. We therefore addressed the possibility that plasmas C11 and C4 contained high levels of immunostimulatory IFN-Is, which might make neutralizing effects undetectable in functional neutralization assays. However, incubation of a highly sensitive reporter cell line with plasmas C11 or C4 alone did not induce IFN-mediated stimulatory activity (Fig. 6 C), confirming that these two plasma samples indeed contained non-neutralizing anti-IFN-I autoAbs. We next hypothesized that non-neutralizing anti-IFN-I autoAbs in plasmas may have only low avidities toward IFN-Is as compared to neutralizing plasmas. Indeed, while rontalizumab and IgGs from three IFNα-neutralizing donor plasmas all exhibited high experimental IFNα2 avidity indexes >0.6 at 6 M urea (Fig. 6 D), the majority of anti-IFNα IgGs from non-neutralizing plasma C11 were removed from IFNα2 with a wash buffer containing 2 M urea and thus had low avidity (Fig. 6 D). Similarly, anti-IFNω IgGs from non-neutralizing plasma C4 lost the ability to bind IFNω following washing with 4 M urea, while anti-IFNω IgGs from three neutralizing donor plasmas remained bound to IFNω (Fig. 6 E). These data indicate that non-neutralizing anti-IFN-I autoAbs may limit the interaction between IFN-Is and a single IFN-I receptor, at least in in vitro assays using unpurified plasmas, but their low overall avidity for IFN-I likely prevents them from functionally inhibiting IFN-I in vivo.

### Development of a rationally engineered signaling-inert mutant IFNα

**Figure 7.**
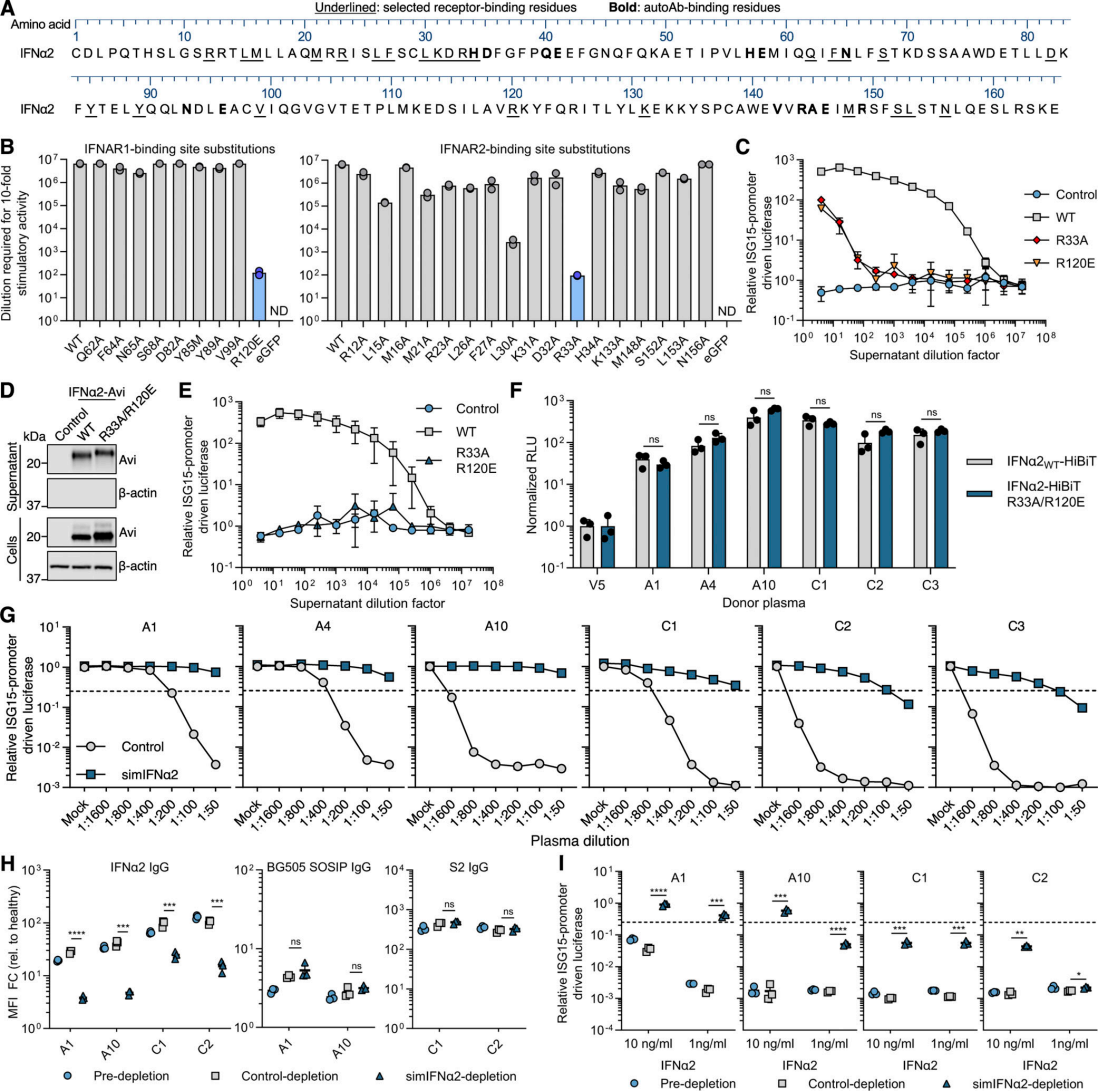
**Development of a simIFNα to counteract anti-IFNα autoAbs. (A)** IFNα2 amino acid sequence with residues targeted by autoAbs in bold, and residues selected for substitution that are bound by IFNAR1/IFNAR2 underlined. Numbering refers to the mature form of IFNα2. **(B)** Immunostimulatory activity of the indicated IFNα2 mutants compared with IFNα2_WT_ on AIR cells at 16 h after stimulation. Input IFNα2 amounts were first normalized to 10^7^ HiBiT luciferase units before the stimulatory activity of each mutant was titrated out using fourfold serial dilutions. The dilutions at which each protein induced AIR cell activity 10-fold over baseline were calculated by nonlinear regression curve fitting using GraphPad Prism 10. Data represent mean values from *n* = 2 replicates. ND: not detectable. Blue bars indicate mutants studied further. **(C)** Immunostimulatory activity of IFNα2_R33A_ and IFNα2_R120E_ mutants using HEK293T cell supernatants at the indicated dilutions. Data are normalized to the luciferase signal from unstimulated cells. Data represent mean values from *n* = 3 replicates. **(D)** Western blot analysis of Avi-tagged IFNα2_R33A/R120E_ protein, as compared to IFNα2_WT_, in cell and supernatant fractions from transfected HEK293T cells. β-Actin was used as a loading/specificity control. **(E)** Immunostimulatory activity of IFNα2_R33A/R120E_, as compared to IFNα2_WT_, on AIR cells at the indicated dilution and at 16 h after stimulation. Data are normalized to the luciferase signal obtained from the unstimulated control. Data represent mean values from *n* = 3 replicates. **(F)** Comparison of anti-IFNα IgG autoAb reactivity with IFNα2_WT_ and IFNα2_R33A/R120E_ proteins using the HiBiT-based qIP assay for six plasmas. Data represent mean values from *n* = 3 replicates. **(G)** Neutralization of 1 ng/ml IFNα2 activity on AIR cells by six plasmas, and inhibition of neutralization by preincubation of plasmas with simIFNα. Data are normalized to the luciferase signals from the mock plasma–treated conditions. **(H)** Relative levels of anti-IFNα IgG autoAbs and virus-specific IgG antibodies (HIV: BG505 SOSIP; COVID: S2) before (pre-) and after plasma depletion using control microparticle beads, or microparticle beads coupled to simIFNα. Data are expressed as MFI FC values made relative to the values derived from six negative control (healthy donor) plasma samples without anti-IFN-I or anti-virus IgG. **(I)** IFNα2 neutralization activities of the indicated anti-IFNα IgG autoAb–positive plasma samples before and after depletion as described in H. Data are normalized to the luciferase signal from a healthy control plasma–treated condition. Dashed lines in G and I indicate neutralization thresholds, set at 25% activity relative to the IFN-only condition (G) or healthy donor control (I). For all data panels, results shown are representative of at least *n* = 2 similar experiments. Statistical significance between groups was determined using unpaired *t* tests (F, H, and I): ns, not significant; *P < 0.05; **P < 0.01; ***P < 0.001; ****P < 0.0001. See also Fig. S4. FC, fold change. Source data are available for this figure: SourceData F7.

**Figure S4.**
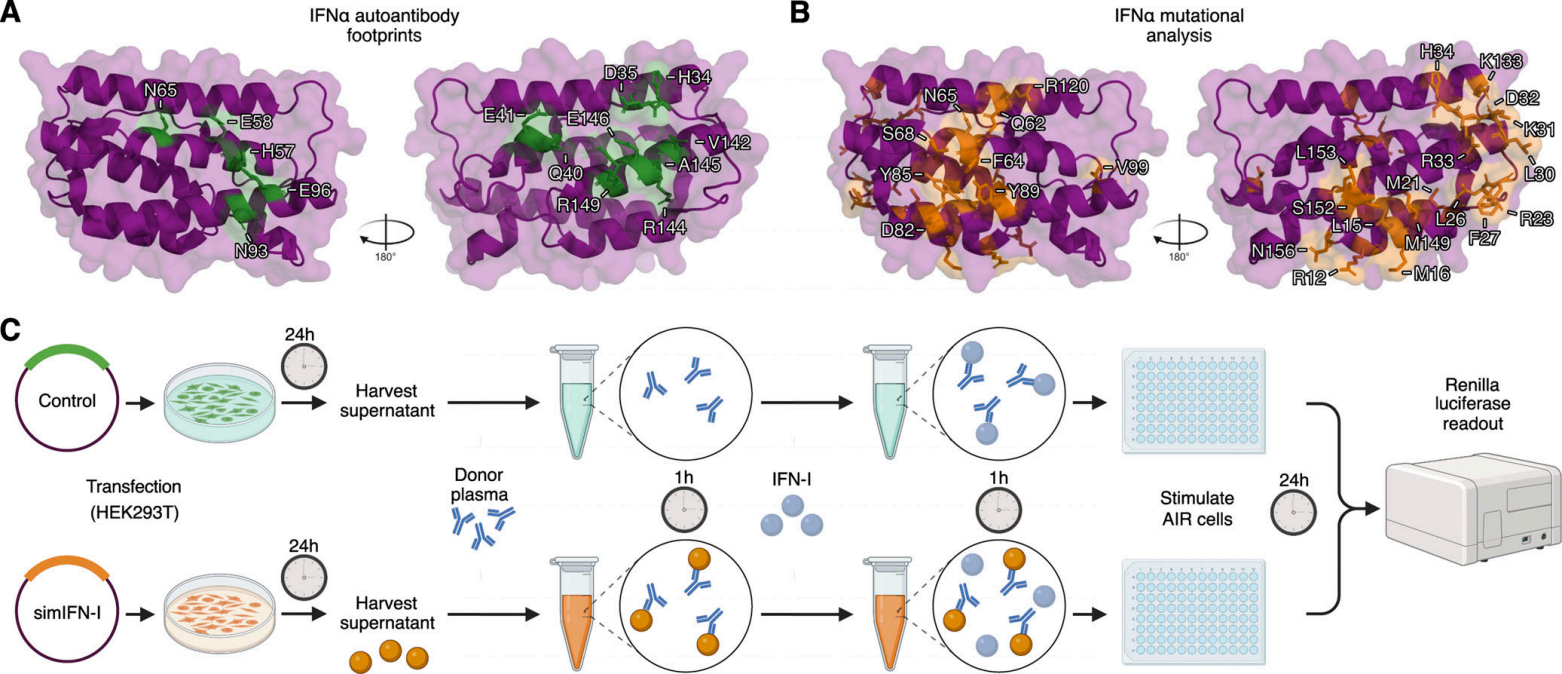
**Design of simIFNα retaining the mapped R1 and R2 autoAb footprints. (A)** Previously described structure of the IFNα2 protein (PDB: 3SE3) with residues in the R1- and R2-footprints colored green. **(B)** Previously described structure of the IFNα2 protein (PDB: 3SE3) with residues selected for mutational analysis, based on their role in mediating interactions with IFNAR1/IFNAR2, colored orange. **(C)** Schematic overview of the assay to determine whether simIFN-Is can block neutralization of IFN-I by anti-IFN-I autoAbs.

Based on the IFNα residues we identified as being recognized by neutralizing plasma anti-IFNα autoAbs, we aimed to develop decoy molecules that could prevent anti-IFNα autoAbs from binding and inhibiting IFNα, with the ultimate goal of restoring natural endogenous antiviral defenses. Given the multiple (and structural) IFNα-footprints herein defined, we focused on the concept of maintaining essential epitopes within the overall structure of IFNα, but ensuring that the resulting IFNα-based decoy molecule generated was a functionally inert protein unable to activate IFN-I signaling. Thus, with the aim of completely removing the ability of IFNα2 to signal via IFNAR1 and IFNAR2, we first performed a targeted mutagenesis functional screen. We independently substituted 10 amino acid residues of IFNα2 that are implicated in mediating the interaction between IFNα and IFNAR1, as well as 16 residues implicated in the interaction with IFNAR2 (Pan et al., 2008; Piehler et al., 2000; Urin et al., 2015). The selected amino acids were mainly outside of the mapped autoAb footprints (Fig. 7 A; and Fig. S4, A and B). All newly generated single amino acid variants of HiBiT-tagged IFNα2 were produced and secreted from HEK293T cells and displayed different degrees of biological signaling activity (Fig. 7 B). IFNα2 substitution R120E was the only IFNAR1-binding site mutant tested that displayed a clear reduction in function, while several IFNα2 substitutions in the IFNAR2-binding site displayed reduced activities, albeit R33A displayed the greatest reduction (Fig. 7 B). Nevertheless, the R120E and R33A substitutions alone did not completely ablate IFNα2 signaling function, particularly at high concentrations in a highly sensitive assay (Fig. 7 C); therefore, these two substitutions were combined in a secondary optimization approach. We confirmed that an IFNα2_R33A/R120E_ mutant could be expressed and secreted from HEK293T cells similar to IFNα2_WT_ (Fig. 7 D) and that IFNα2_R33A/R120E_ was completely nonfunctional at stimulating cells and inducing ISG expression, even at high concentrations (Fig. 7 E). Importantly, the introduction of these two substitutions did not reduce the ability of anti-IFNα autoAbs in the plasma to bind IFNα2_R33A/R120E_, as six selected plasma samples (three from the Aged cohort and three from the COVID ICU cohort) demonstrated equal immunoprecipitation capacity for both IFNα2_WT_ and IFNα2_R33A/R120E_ (Fig. 7 F). We herein refer to the IFNα2_R33A/R120E_ protein as a rationally engineered signaling-inert mutant IFNα (simIFNα).

### simIFNα as a functional decoy, and specific depletion agent, for anti-IFNα autoAbs

We next aimed to explore the proof-of-concept therapeutic potential of simIFNα. To this end, we established an assay to assess the ability of simIFNα to act as a decoy protein that prevents plasma anti-IFNα autoAbs from inhibiting bioactive IFNα (Fig. S4 C). A dilution series of anti-IFNα autoAb–positive plasma samples (or mock) was mixed with control and then incubated with 1 ng/ml IFNα2 prior to assessment of remaining functional IFNα2 activity. As expected, the six tested anti-IFNα autoAb–positive plasmas neutralized IFNα2 in a dose-dependent manner (Fig. 7 G). However, when the six anti-IFNα autoAb–positive plasmas were first mixed with simIFNα before being incubated with IFNα2, the anti-IFNα autoAb–positive plasmas failed to efficiently neutralize IFNα2 activity (Fig. 7 G). In addition, we explored the possible use of simIFNα as a tool to specifically deplete anti-IFNα autoAbs from plasma samples, which could have applications in future optimized plasmapheresis efforts to remove anti-IFNα autoAbs from blood (de Prost et al., 2021). Thus, simIFNα was coupled to magnetic microparticles and incubated with a set of anti-IFNα autoAb–positive plasma sample dilutions before the microparticles were removed along with any captured molecules. The levels of various antibodies in plasma samples were then assessed. simIFNα–microparticle capture treatment of all four plasma samples tested (two from the Aged cohort and two from the COVID ICU cohort) resulted in a significant reduction of anti-IFNα IgG levels, while specific antiviral IgGs against HIV-1 or SARS-CoV-2 antigens (HIV Env: anti-BG505 SOSIP; SARS-CoV-2 Spike: anti-S2) were unaffected (Fig. 7 H). Furthermore, this treatment significantly reduced the ability of all four anti-IFNα autoAb–positive plasmas to neutralize IFNα2 at high (10 ng/ml) or low (1 ng/ml) doses (Fig. 7 I). Nevertheless, complete autoAb depletion from plasmas with high neutralizing titers (such as plasma C2) was inefficient with this experimental setup, and an amount of anti-IFNα autoAbs that could partially neutralize low IFNα2 doses remained (Fig. 7 I). Together, these data indicate that binding of anti-IFNα autoAbs to simIFNα can act as a decoy to prevent their binding and inhibition of bioactive IFNα. Furthermore, immobilized simIFNα can be used to specifically capture and deplete anti-IFNα autoAbs from plasmas without removing important virus-specific antibodies that may be critical to combat ongoing infections.

### Development of signaling-inert mutant IFNω

**Figure 8.**
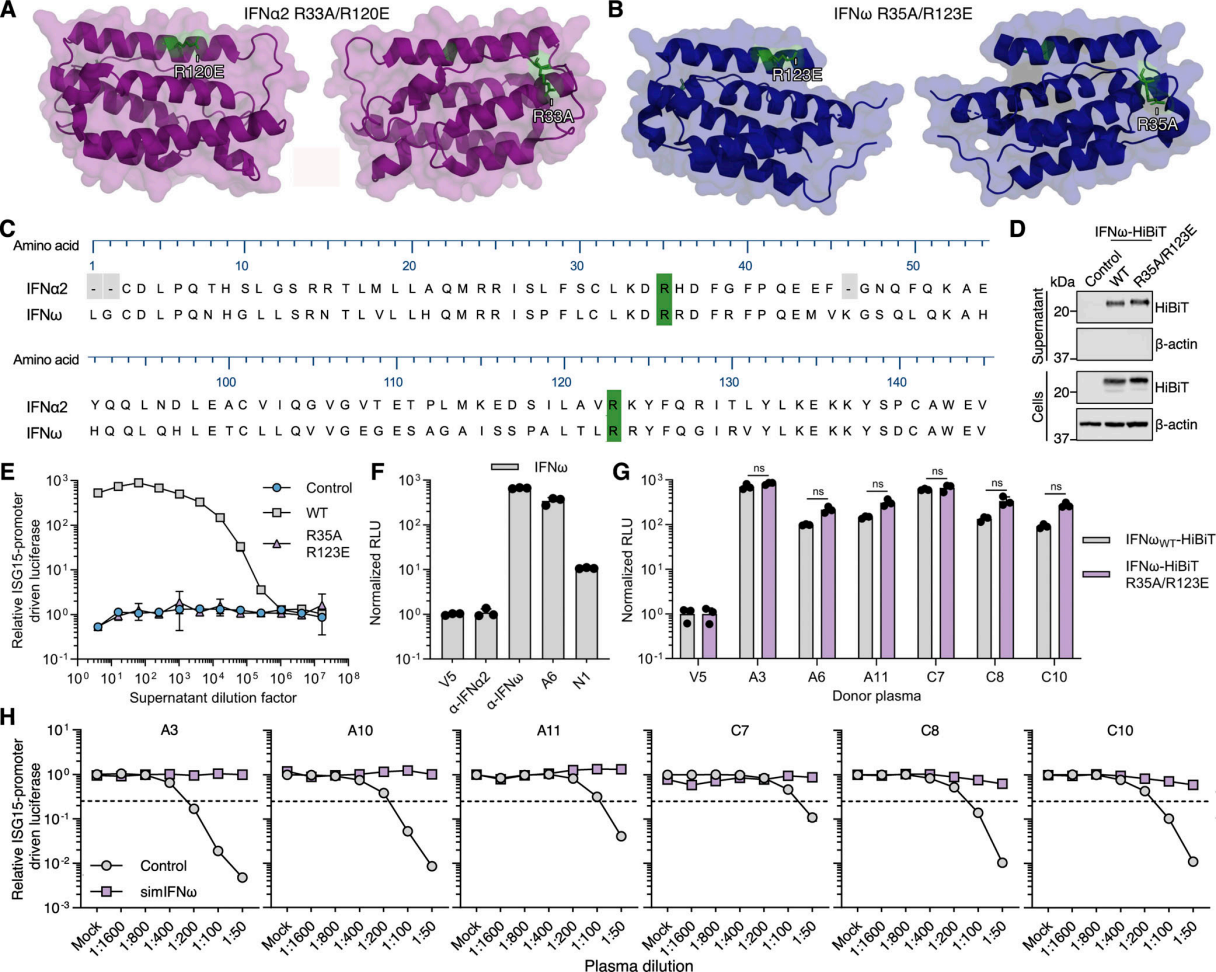
**Development of a simIFNω to counteract anti-IFNω autoAbs. (A)** Structure of the IFNα2 protein (PDB: 3SE3) with amino acid substitutions R33A and R120E (simIFNα) colored green. **(B)** Structure of the IFNω protein (PDB: 3SE4) with amino acid substitutions mimicking those of simIFNα colored green. **(C)** Amino acid sequence alignment of IFNα2 and IFNω with residues substituted to generate simIFN-Is colored green. Amino acid numbering refers to the mature form of IFNω. **(D)** Western blot analysis of HiBiT-tagged IFNω_R35A/R123E_ protein, as compared to IFNω_WT_, in cell and supernatant fractions from transfected HEK293T cells. β-Actin was used as a loading/specificity control. **(E)** Immunostimulatory activity of IFNω_R35A/R123E_, as compared to IFNω_WT_, on AIR cells at the indicated dilution and at 16 h after stimulation. Data are normalized to the luciferase signal from unstimulated control cells. Data represent mean values from *n* = 3 replicates. **(F)** Validation of the HiBiT-based qIP assay for the immunoprecipitation of the IFNω protein using negative control (anti-V5, anti-IFNα2) or positive control (anti-IFNω) antibodies, as well as plasmas A6 (positive) or N1 (negative). Data represent mean values from *n* = 3 replicates. **(G)** Comparison of anti-IFNω IgG autoAb reactivity to IFNω_WT_ and IFNω_R35A/R123E_ proteins using the HiBiT-based qIP assay for six plasmas. Data represent mean values from *n* = 3 replicates. **(H)** Neutralization of 1 ng/ml IFNω activity on AIR cells by six plasmas, and inhibition of neutralization by preincubation of plasmas with simIFNω. Data are normalized to the luciferase signals from the mock plasma–treated conditions. Dashed lines indicate neutralization thresholds, set at 25% activity relative to the IFN-only condition. For all data panels, results are representative of at least *n* = 3 similar experiments. Statistical significance between groups was determined by unpaired *t* tests (G): ns, not significant. See also Fig. S4. Source data are available for this figure: SourceData F8.

The human IFNα and IFNω subtypes are relatively conserved at both the amino acid sequence and structural levels (reviewed in Bekisz et al. [2004]). We therefore hypothesized that the optimized substitutions introduced into IFNα2 to generate the simIFNα protein could be mimicked in IFNω to also generate signaling-inert mutant IFNω (simIFNω) with decoy properties. Based on the sequences and structures of IFNα and IFNω, an IFNω protein harboring the substitutions R35A/R123E was therefore generated as an analogy to IFNα2_R33A/R120E_ (Fig. 8, A–C). The IFNω_R35A/R123E_ protein could be produced and secreted from HEK293T cells at levels similar to IFNω_WT_ (Fig. 8 D), but as predicted had no detectable function in promoting ISG expression when analyzed in a highly sensitive reporter assay (Fig. 8 E). Since the design of simIFNω was not based on extensive antibody footprinting as done for simIFNα, we assessed whether IFNω residues R35 and R123 were important for binding of anti-IFNω autoAbs. We therefore established a qIP assay for IFNω using commercially available anti-IFNα or IFNω mAbs, as well as anti-IFNω autoAb–positive and autoAb–negative plasma samples (Fig. 8 F), before applying this assay to assess plasma autoAb interactions with IFNω_R35A/R123E_. Six selected anti-IFNω autoAb–positive plasma samples (three from the Aged cohort and three from the COVID ICU cohort) were used to immunoprecipitate HiBiT-tagged IFNω_WT_ or IFNω_R35A/R123E_. All six plasma samples displayed similar abilities to immunoprecipitate IFNω_R35A/R123E_ as compared to IFNω_WT_ (Fig. 8 G), demonstrating that IFNω R35 and R123 residues are not essential for recognition by anti-IFNω autoAb IgGs. We next sought to demonstrate the ability of simIFNω to act as a decoy molecule and prevent anti-IFNω autoAbs from inhibiting bioactive IFNω (similar to the assay described in Fig. S4 C). A dilution series of anti-IFNω autoAb–positive plasma samples (or mock) was mixed with control and then incubated with 1 ng/ml IFNω prior to assessment of remaining functional IFNω activity. As expected, the six anti-IFNω autoAb–positive plasmas selected for testing were able to neutralize IFNω in a dose-dependent manner (Fig. 8 H). However, when the six anti-IFNω autoAb–positive plasmas were first mixed with simIFNω before being incubated with IFNω, the anti-IFNω autoAb–positive plasmas failed to efficiently neutralize IFNω activity (Fig. 8 H). These data indicate that simIFNω retaining important autoAb-binding epitopes can be used to prevent anti-IFNω autoAb–positive plasmas from inhibiting IFNω function.

### simIFN-Is can restore the antiviral activity of IFN-I in the presence of anti-IFN-I autoAbs

**Figure S5.**
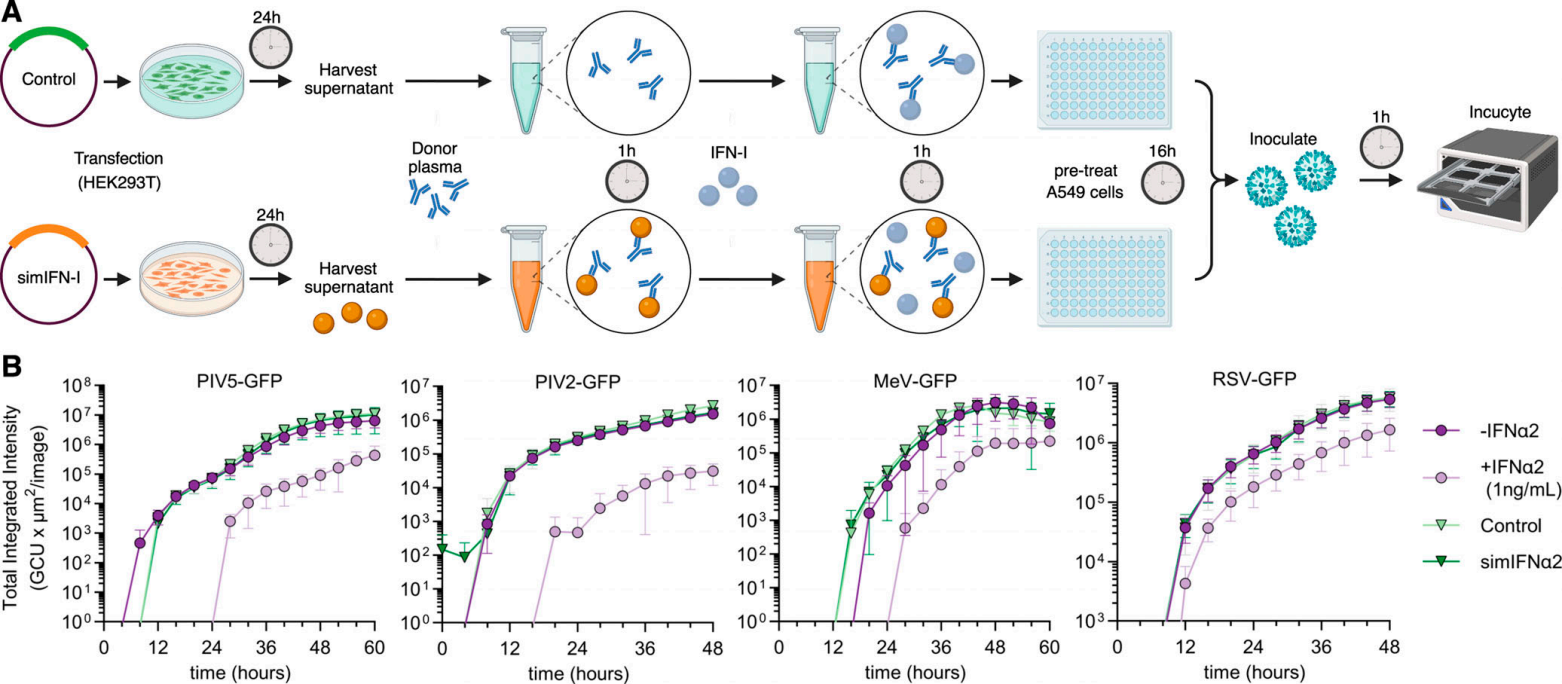
**simIFNα and virus replication. (A)** Schematic overview of the assay to determine whether simIFN-Is restore the antiviral function of IFN-I in the presence of plasma anti-IFN-I autoAbs. **(B)** simIFNα alone has no antiviral activity. Replication kinetics of four GFP-expressing viruses in A549 cells that were pretreated for 16 h with the conditions indicated (simIFNα used). Cells were inoculated with an MOI of 0.01 FFU/cell for PIV5-GFP, 0.1 FFU/cell for PIV2-GFP, 0.03 FFU/cell for MeV-GFP, and 0.1 TCID_50_/cell for RSV-GFP. The GFP signal was monitored with the IncuCyte live-cell imaging system as a surrogate readout for viral replication. Mean values from *n* = 3 replicates are shown. Error bars indicate standard deviations.

**Figure 9.**
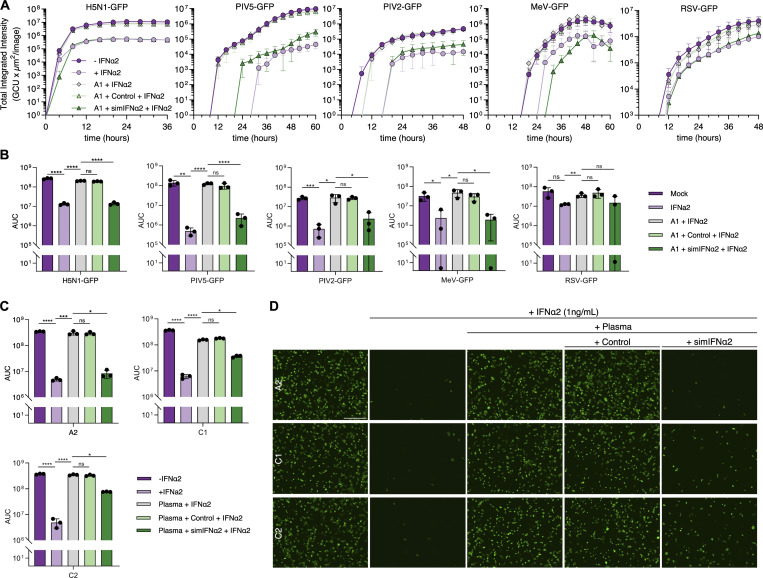
**simIFN-I can restore the antiviral activity of IFN-I in the presence of neutralizing anti-IFN-I autoAbs. (A)** Replication kinetics of five GFP-expressing viruses in A549 cells that were pretreated for 16 h with the conditions indicated (simIFNα used with IFNα-neutralizing plasma A1). Cells were inoculated with an MOI of 0.1 PFU/cell for H5N1-GFP, 0.01 FFU/cell for PIV5-GFP, 0.1 FFU/cell for PIV2-GFP, 0.03 FFU/cell for MeV-GFP, and 0.1 TCID_50_/cell for RSV-GFP. The GFP signal was monitored with the IncuCyte live-cell imaging system as a surrogate readout for viral replication. Data represent mean values from *n* = 3 replicates, and error bars indicate standard deviations. **(B)** AUC values of data shown in A. Data represent mean values from *n* = 3 independent experiments, and error bars indicate standard deviations. **(C)** AUC values of H5N1-GFP replication in A549 cells that were pretreated with similar conditions as described in A, using additional IFNα-neutralizing donor plasma samples as indicated. Data represent mean values from *n* = 3 independent experiments, and error bars indicate standard deviations. **(D)** Fluorescence images of A549 cells infected with H5N1-GFP from the experiments described in C. Images are from 12 h after inoculation, where virus replication had reached peak total integrated intensity values. The scale bar represents 200 μm. For all data panels, results are representative of *n* = 3 similar experiments. Statistical significance between groups was determined by unpaired *t* tests (B and C): ns, not significant; *P < 0.05; **P < 0.01; ***P < 0.001; ****P < 0.0001. See also Fig. S5. AUC, area under the curve.

Finally, we evaluated the potential for our newly created simIFN-Is to inhibit the ability of anti-IFN-I autoAbs to promote virus replication. Thus, as proof-of-concept, cells were pretreated with IFNα2 alone or with IFNα2 preincubated with a donor plasma (A1) containing neutralizing anti-IFNα autoAbs. 16 h later, cells were inoculated with various GFP-expressing respiratory RNA viruses (influenza A virus, H5N1-GFP; respiratory syncytial virus, RSV-GFP; measles virus, MeV-GFP; parainfluenza virus 2, PIV2-GFP; or parainfluenza virus 5, PIV5-GFP), and virus replication was monitored over several days. In parallel, similar experiments were performed using cells pretreated with IFNα2 that had been preincubated with the donor plasma containing neutralizing anti-IFNα autoAbs together with simIFNα (or control) (Fig. S5 A). Treatment of cells with 1 ng/ml IFNα2 strongly attenuated the replication of all five viruses tested, while preincubation of IFNα2 with the neutralizing A1 plasma prevented IFNα2 from exerting antiviral activity, and virus replication was similar to that observed in unstimulated (−IFNα2) cells (Fig. 9 A). Notably, simIFNα (but not control) treatment of neutralizing A1 plasma–containing conditions led to a restoration of IFNα2 antiviral activity and reduced virus replication (Fig. 9 A). This antiviral effect was not due to any direct action of simIFNα alone, as pretreatment of cells with simIFNα in the absence of bioactive IFNα2 or anti-IFNα autoAbs did not inhibit virus replication (Fig. S5 B). The impact of simIFNα could be readily quantified over multiple independent experiments, showing clear (and mostly significant) effects for all viruses tested (Fig. 9 B). Furthermore, simIFNα (but not control) treatment led to complete restoration of IFNα2 antiviral activity with neutralizing plasma A2, and significant restoration of IFNα2 antiviral activity with neutralizing plasmas C1 and C2 (Fig. 9 C). The effective impact of simIFNα was also clearly apparent in the live-cell fluorescent images taken during the course of the experiments (Fig. 9 D). Together, these data demonstrate that simIFN-Is retaining anti-IFN-I autoAb–binding epitopes can be used as decoy proteins to restore the antiviral activity of bioactive IFN-I.

## Discussion

Over the past few years, there has been a growing appreciation for the association of anti-IFN-I autoAbs with severe viral disease outcomes. In particular, the presence of anti-IFNα or anti-IFNω autoAbs appears to increase disease severity caused by viruses that individuals may be encountering for the first time, and to which adaptive immune responses are absent (e.g., SARS-CoV-2 [Abers et al., 2021; Barzaghi et al., 2025; Bastard et al., 2020, 2021a, 2021d; Chauvineau-Grenier et al., 2022; Eto et al., 2022; Koning et al., 2021; Manry et al., 2022; Zhang et al., 2020b], MERS-CoV [Alotaibi et al., 2023], TBEV [Gervais et al., 2024b], West Nile virus [Gervais et al., 2023], other arboviruses [Gervais et al., 2024a], and live-attenuated yellow fever virus vaccines [Bastard et al., 2021c]), or caused by viruses to which previously developed adaptive immune responses may have waned (e.g., seasonal influenza viruses [Zhang et al., 2022b], herpesviruses [Busnadiego et al., 2022]). Under these scenarios, the innate IFN-I system is critically important to compensate for absences in humoral immunity. Given that anti-IFN-I autoAbs have been identified in ∼2–4% of the general population over the age of 70 years (Bastard et al., 2021a; Fernbach et al., 2024), a significant proportion of the aging human population is at constant risk of developing severe disease caused by either future emerging viruses or common viruses to which adaptive defenses have been compromised. This highlights the urgent need for effective prophylactic or therapeutic strategies to counteract anti-IFN-I autoAb functions and thus restore effective innate antiviral IFN-I defenses.

Since anti-IFNβ autoAbs are relatively rare (Bastard et al., 2020; Fernbach et al., 2024), patients harboring anti-IFNα or anti-IFNω autoAbs and suffering from severe viral infections have been treated with IFNβ in early trials to explore whether the negative effects of anti-IFNα/ω autoAbs could be bypassed (Bastard et al., 2021b). However, current anecdotal evidence of efficacy is limited to only one individual who was treated in the early stages of infection (Bastard et al., 2021b), and treatment with excess IFN-I is known to have potentially severe and undesirable pro-inflammatory consequences (WHO Solidarity Trial Consortium et al., 2021; Davidson et al., 2014; Major et al., 2020). Furthermore, we recently reported that an individual prone to developing autoAbs generated lifelong neutralizing anti-IFNα autoAbs following IFNα therapy (Fernbach et al., 2024), suggesting that treatment of tolerance-compromised individuals with high doses of IFNβ could also result in the induction of pathogenic anti-IFNβ autoAbs and new long-term infection-susceptibility consequences. Alternative plasmapheresis efforts to remove anti-IFN-I autoAbs from the circulating blood of patients with severe COVID-19 have also not been associated with improved clinical outcomes (de Prost et al., 2021). This may be because current plasmapheresis procedures also remove virus-specific antibodies that may have already developed and that may be critical to clear the infection. Herein, we therefore explored the potential of a new therapeutic strategy by undertaking a fundamental proof-of-concept approach. We mapped major residues on IFNα bound by donor plasmas and used this information to rationally engineer simIFN-I structural mimetics. We validated these simIFN-Is in vitro as decoy proteins capable of binding anti-IFNα and anti-IFNω autoAbs, thereby restoring normal IFN-I immune function without hyperstimulation. Importantly, the antibody footprints we identified as being bound by anti-IFN-I autoAbs were universally recognized by all donor plasmas tested across two independent cohorts, implying the likely generality of our strategy. We also demonstrated that such simIFN-Is can be used to deplete anti-IFN-I autoAbs from plasmas ex vivo, in a similar way to plasmapheresis, but without depletion of other virus-specific antibodies. Another potential use of simIFN-Is could be to aid in targeting the specific B cells producing pathogenic autoAbs for depletion or killing via immunotherapeutics (Kreitman, 2006; Wang et al., 2024), as these engineered simIFN-Is should specifically bind the relevant B cell receptors without engaging IFNAR1 or IFNAR2. Thus, further development of such proof-of-concept strategies, and perhaps their future additional application to rare anti-IFNβ autoAbs, has the potential to create a therapeutic framework to negate the pathogenic effects of anti-IFN-I autoAbs and ultimately to restore the body’s own natural IFN-I–mediated antiviral defenses. While we provide evidence to support this concept in two distinct infectious disease cohorts, we hypothesize that our results will be broadly applicable to other groups of individuals harboring anti-IFN-I autoAbs.

A fundamental finding from our study is also the identification of the pathogenic mechanism of action by which anti-IFN-I autoAbs neutralize the function of IFN-Is. All donor plasmas that we tested from two independent cohorts of individuals with neutralizing anti-IFNα autoAbs contained IgGs recognizing the same two IFNα faces that overlap with the IFNα-binding sites for IFNAR1 and IFNAR2. Indeed, we noted only a very small fraction of autoAbs (∼1–5%) that bound IFNα outside of the main footprints. This observation, using both full-length linearized and full-length folded antigens, contrasts with a previous interpretation of linear peptide microarray data from a limited number of donor plasma samples that suggested that anti-IFNα autoAbs may only block the interaction between IFNα and IFNAR1 (Aoki et al., 2024). It is unknown whether donor disease phenotype or autoAb detection methods may bias the types of autoAbs identified. Furthermore, we could show experimentally that high-titer neutralizing anti-IFN-I autoAb–containing plasmas (against either IFNα or IFNω) blocked the independent interaction of each IFN-I with both IFNAR1/2 receptor subunits, underlining a clear polyclonal response that functionally targets at least two different epitopes. Importantly, we also noted that high-titer non-neutralizing anti-IFN-I autoAb–containing plasmas could also limit IFN-I interactions with receptors, but this was typically limited to affecting only a single receptor subunit (either IFNAR1 or IFNAR2). The IgG:IFN-I interaction was also of relatively low avidity, at least in unpurified plasma samples, which likely explains the overall lack of in vivo inhibitory function. The observation that donor plasmas containing relatively high-avidity neutralizing autoAbs block engagement of IFNα to both IFNAR1 and IFNAR2 suggests that such autoAbs would prevent the critical IFNAR1:IFNAR2 heterodimerization event required for IFN-I signaling, while low avidity non-neutralizing autoAbs may still allow IFNAR1:IFNAR2 heterodimerization to occur.

IFNα and IFNω proteins share a high structural and sequence homology (∼60% amino acid sequence identity). Given that a large proportion of donors with anti-IFN-I autoAbs harbor neutralizing IgGs targeting IFNα and IFNω proteins, this raises the possibility that some autoAbs may be cross-reactive and neutralize both IFNα and IFNω. Indeed, most plasmas selected for this study contained neutralizing autoAbs against both IFNα and IFNω, and previous work isolated both cross-reactive and subtype-specific high-avidity anti-IFN-I autoAbs (Meyer et al., 2016). Notably, the two plasmas we studied with non-neutralizing anti-IFNα or anti-IFNω autoAbs contained neutralizing autoAbs against the other respective IFN-I. It is tempting to speculate that high-avidity neutralizing autoAbs against one IFN-I subtype may, in some instances, engage another IFN-I subtype with low avidity and in a non-neutralizing manner. A general understanding of autoAb cross-reactivity between different IFN-Is will be important to uncover in the future, and could have implications for understanding the clinical consequences of such antibodies, as well as how to inhibit them.

Our antibody footprinting and mechanistic findings also have implications for understanding the origin of anti-IFN-I autoAbs, at least in the cohorts tested, as the data imply that the autoAbs have been raised against whole IFN-I molecules, possibly as a result of IFN-I immunotriggering in the context of global loss of self-tolerance (Fernbach et al., 2024). This would appear to contrast with the narrower, single, autoAb-bound epitope mainly reported for anti-IFN-II (IFNγ) autoAbs, which have been suggested to be induced by molecular mimicry with a single peptide sequence from a fungal pathogen (Lin et al., 2016).

In summary, we identified that two dominant IFN-I regions are commonly recognized by neutralizing anti-IFN-I autoAbs in the plasmas of donors from two distinct autoAb-positive cohorts (severe COVID-19 and aged individuals living with HIV-1). We provide experimental evidence that neutralizing anti-IFN-I autoAb–positive plasmas have higher avidities than non-neutralizing plasmas, and consequently exert their pathogenic effects by blocking the interactions of IFN-Is with IFNAR1 and IFNAR2 receptor subunits. Building on these findings, we rationally designed simIFN-Is that maintain the key structural epitopes, can bind anti-IFNα and anti-IFNω autoAbs, and can be used as decoys or depletion tools to specifically prevent the neutralizing effects of anti-IFN-I autoAbs. Engineered simIFN-Is thereby have the potential to restore the body’s own natural antiviral defenses and serve as a proof-of-concept treatment strategy for patients with exacerbated viral disease caused by pathogenic anti-IFN-I autoAbs.

## Materials and methods

### Human plasma samples and ethics

Anonymized human plasma samples previously categorized as positive or negative for anti-IFNα and/or anti-IFNω IgG autoAbs were used (Busnadiego et al., 2022; Fernbach et al., 2024). The samples were originally derived from specimens stored in the biobanks of the Swiss HIV Cohort Study (Scherrer et al., 2022) (SHCS; Aged cohort; A series; *n* = 12 autoAb-positive samples [2 positive against IFNα only (A1, A2), 8 positive against both IFNα and IFNω (A3–A10), and 2 positive against IFNω only (A11, A12)], *n* = 6 autoAb-negative samples that were used as controls; N series) or the MicrobiotaCOVID Cohort Study (Buehler et al., 2021) (COVID ICU cohort; C series; *n* = 12 autoAb-positive samples against IFNα and/or IFNω [10 neutralized both IFNα and IFNω (C1–C3, C5–C10, C12), one neutralized IFNα and had non-neutralizing anti-IFNω autoAbs (C4), and one neutralized IFNω and had non-neutralizing anti-IFNα autoAbs (C11)]), which cover their use in the current work. Detailed information on both studies is available at http://www.shcs.ch or https://clinicaltrials.gov (ClinicalTrials.gov Identifier: NCT04410263). The SHCS has been approved by the local ethics committees of all participating institutions (Kantonale Ethikkommission Bern, Ethikkommission des Kantons St. Gallen, Comite Departemental d’Ethique des Specialites Medicales et de Medicine Communataire et de Premier Recours, Kantonale Ethikkommission Zürich, Repubblica et Cantone Ticino–Comitato Ethico Cantonale, Commission Cantonale d’Étique de la Recherche sur l’Être Humain, Ethikkommission beider Basel), and written informed consent has been obtained from all participants. The MicrobiotaCOVID Cohort Study was approved by the local ethics committee (Kantonale Ethikkommission Zürich BASEC ID 2020-00646) in accordance with the provisions of the Declaration of Helsinki and the Good Clinical Practice guidelines of the International Conference on Harmonization. The local ethics committees of additional study sites (Comitè Ètic d’Investigació amb medicaments Institut d’Investigació Sanitària Pere Virgili and Comitè Ètic d’Investigació clînica Servei Andorrà d’atenciò sanitària) also approved the study at their centers according to the BASEC ID 2020-00646 protocol. As described previously (Abela et al., 2021; Busnadiego et al., 2022), additional leftover healthy donor plasma samples (*n* = 6) were derived from specimens provided by the Zurich Blood Transfusion Service of the Swiss Red Cross and were used with approval of the responsible Local Ethics Committee of the Canton of Zurich, Switzerland (Kantonale Ethikkommission Zurich BASEC ID 2021-00437 and 2021-01138). All data were analyzed anonymously.

### Cells and viruses

HEK293T, Vero-CCL81, A549, MDCK, and HEp-2 cells were all originally from the ATCC. A549-interferon-reporter (AIR) cells have been described in depth previously (Groen et al., 2024). All cells were cultured in Dulbecco’s modified Eagle’s medium (DMEM) supplemented with 10% fetal bovine serum (FBS) and 100 U/ml penicillin and streptomycin (15140-122; Gibco). Cells were grown at 37°C with 5% CO_2_. The low-pathogenic H5N1-GFP reporter virus (recombinant influenza virus A/Vietnam/1203/2004 encoding HA without the multibasic cleavage site; HALo) was a kind gift from Balaji Manicassamy (University of Iowa, Iowa City, IA, USA [Kandasamy et al., 2020]) and was propagated in MDCK cells. Virus titers, expressed as plaque-forming units (PFU)/ml, were determined by plaque assay as described previously (Hale et al., 2006). GFP-encoding parainfluenza virus 2 strain V94(15C) (PIV2-GFP; P222; ViraTree), parainfluenza virus 5 strain WA3 (PIV5-GFP, P523; ViraTree), and measles virus strain Edmonston (MeV-GFP, OV1006; Imanis Life Sciences) were propagated in Vero-CCL81 cells, and titers expressed as focus-forming units (FFU)/ml were determined by focus-forming assay as similarly described (Hale et al., 2006). GFP-encoding respiratory syncytial virus lineage A2 (R125; ViraTree) was propagated in HEp-2 cells, and titers, expressed as 50% tissue culture infective dose (TCID_50_)/ml, were determined by endpoint dilution in HEp-2 cells as described previously (Groen et al., 2022).

### Plasmids

A plasmid encoding eGFP- and V5-tagged IFNα2 lacking the signal peptide required for secretion (pcDNA3.1-eGFP-V5-IFNα2) was generated with GeneArt gene synthesis services (Thermo Fisher Scientific). Plasmids encoding N-terminal IFNα2 deletions were generated by PCR-mediated deletion of plasmid DNA using pcDNA3.1-eGFP-V5-IFNα2 as a template DNA (Lee et al., 2004). Plasmids encoding C-terminal IFNα2 deletions were generated by introducing a premature stop codon using the QuikChange site-directed mutagenesis kit (200518; Agilent) according to the manufacturer’s instructions. Plasmids encoding full-length secreted IFNα2 with a C-terminal Avi-tag or HiBiT-tag were generated with GeneArt gene synthesis services (Thermo Fisher Scientific). A plasmid encoding full-length secreted IFNω with a C-terminal HiBiT-tag (pcDNA3.1-IFNω-HiBiT) was generated with GeneArt gene synthesis services (Thermo Fisher Scientific). A plasmid encoding IFNω with a C-terminal Avi-tag was generated by PCR-mediated deletion and insertion using plasmid pcDNA3.1-IFNω-HiBiT as the template (Lee et al., 2004). Primers were designed that contained binding regions flanking the HiBiT-tag sequence and nonbinding tails containing the Avi-tag sequence, which were used to replace the HiBiT-tag with an Avi-tag. Plasmids encoding IFNα2 or IFNω mutant constructs that contained single or double amino acid substitutions were generated using the QuikChange site-directed mutagenesis kit (200518; Agilent) according to the manufacturer’s instructions. Constructs with multiple mutations were generated with GeneArt gene synthesis services (Thermo Fisher Scientific). A plasmid encoding GFP with an N-terminal HA-tag was from Addgene (HA-GFP, #137763; Addgene, a kind gift from Carol Mercer [University of Cincinnati, Cincinnati, OH, USA] [Stefely et al., 2020]) and used as a control plasmid for assays.

### Transfections

150,000 HEK293T cells were seeded per well in a 24-well tissue culture plate and incubated overnight. Cells were transfected with 0.5 µg plasmid DNA per well using FuGENE HD transfection reagent (E2311; Promega) according to the manufacturer’s instructions. After 24 h, cells or supernatants were processed as described below.

### IFN-I activity assays

For assessing relative IFN-I activities, HEK293T cells were transfected as described above with the appropriate IFN-I plasmid. Cell supernatants were harvested 24 h after transfection and centrifuged at 1,000 *g* for 5 min to pellet cell debris. As required, HiBiT-tagged IFN-Is in soluble fractions were normalized (see methodology below) and then serially diluted as indicated in Opti-MEM containing EnduRen (1:10,000 final concentration) and used to stimulate 30,000 AIR cells (that were seeded the day before in 96-well white-bottomed tissue culture plates in Opti-MEM) for 24 h before Renilla luciferase activity levels were determined using a PerkinElmer EnVision plate reader (EV2104). Luciferase values were divided by the average of mock-stimulated cells and expressed as relative Renilla activities (relative ISG15 promoter–driven luciferase). For assessment of plasmas neutralizing IFNα or IFNω, 30,000 AIR cells were seeded per well in a 96-well white-bottomed tissue culture plate in Opti-MEM and incubated overnight. Donor plasmas (or mock) were diluted 1:20 in Opti-MEM containing IFNα2 (NPB2-34971; Novus Biologicals) or IFNω (NBP2-35893; Novus Biologicals) at a final concentration of 0.2, 1, or 10 ng/ml, as well as the live-cell Renilla luciferase substrate (EnduRen, E6481; Promega; 1:10,000), and were incubated together for 1 h at room temperature with constant shaking at 600 rpm. The plasma:IFN-I mixtures were then used to stimulate AIR cells for 24 h before Renilla luciferase activity levels were determined using a PerkinElmer EnVision plate reader (EV2104). Luciferase values were divided by the average of those of the healthy donors (expressed as relative ISG15 promoter–driven luciferase), and the threshold for neutralization was set at either 25% activity or the mean minus 5 standard deviations of the healthy donors.

### Quantitative detection of specific IgG and neutralizing anti-IFN-I autoAbs

Detection of anti-IFNα and anti-IFNω IgG autoAbs in donor plasmas was performed using a multiplexed bead-based assay as described previously (Busnadiego et al., 2022; Fernbach et al., 2024), using a 1:100 dilution of plasma. Similar assays were employed to detect anti-HIV1 Env (BG505 SOSIP) IgG produced as described previously (Kadelka et al., 2018), and anti-SARS-CoV-2 Spike (S2) IgG (Abela et al., 2021). To determine avidity indexes, beads incubated with donor plasmas or mAbs were washed once with assay buffer (1% BSA in PBS) containing increasing amounts of urea (0–8 M) for 5 min prior to incubation with the secondary antibody. The avidity index was defined as the remaining fraction of bound IgG in the presence of urea, calculated by dividing the mean fluorescence intensity (MFI) of the sample treated with urea by the MFI of the sample not treated with urea (Klasse, 2016). High avidity was defined as an index >0.6 at 6 M urea, while low avidity was defined as <0.6 at 6 M urea (Bauer et al., 2021; Prince and Lapé-Nixon, 2014). Neutralizing anti-IFN-I autoAbs were detected as described previously (Groen et al., 2024), using commercial anti-IFNα (551795; BD Biosciences) or anti-IFNω (NBP3-06154; Novus Biologicals) antibodies with neutralizing activity as positive controls (pos; 1:100 dilution), and six plasma samples from donors without anti-IFN-I autoAbs as negative controls (control; 1:100 dilution).

### Western blotting

Cells or supernatants were lysed in urea disruption buffer (3 M urea, 1 M β-mercaptoethanol, 2% SDS) and sonicated to shear nucleic acids. Proteins then were separated on Bolt 4–12% Bis-Tris Plus Gels (NW04120; Thermo Fisher Scientific) and transferred onto 0.45 µm nitrocellulose membranes (10600008; Amersham) according to the manufacturer’s instructions. Membranes were blocked using 10% milk in PBS supplemented with 0.1% Tween-20 (PBS-T) for 30 min followed by incubation with primary antibody diluted in 1% milk in PBS-T overnight at 4°C. Primary antibodies used were as follows: β-actin (1:5,000, sc-47778; Santa Cruz), V5-tag (1:2,000, MCA1360; Bio-Rad), Avi-tag (1:2500, MAB10546; Bio-Techne), HiBiT-tag (1:1,000, N7200; Promega), or individual plasmas (1:500). Membranes were washed three times with PBS-T and incubated with secondary antibody (1:5,000 dilution) for 1 h at room temperature. Secondary antibodies used were as follows: IRDye 800CW goat anti-mouse IgG (926-32210; Li-COR Biosciences), IRDye 800CW goat anti-rabbit IgG (926-32211; Li-COR Biosciences), IRDye 800CW goat anti-human IgG (926-32232; Li-COR Biosciences), and IRDye 680RD goat anti-mouse IgG (926-68070; Li-COR Biosciences). Membranes were then washed three times with PBS-T, and the signal was detected using an Odyssey Fc Imager (Li-COR Biosciences) followed by quantification using Image Studio Lite Quantification Software (Li-COR Biosciences).

### HiBiT-based qIP assays

Cells transfected with wild-type or mutant IFNα or IFNω with a C-terminal HiBiT-tag were lysed using 400 µl IP buffer (50 mM Tris-HCl, pH 7.5, 150 mM NaCl, 1 mM EDTA, 1% Triton X-100) supplemented with cOmplete Mini EDTA-free protease inhibitors (11836170001; Roche), and were centrifuged for 5 min at 1,000 *g* to pellet cell debris. HiBiT-normalized amounts of soluble fractions (see methodology below) were then incubated overnight with 1 µg anti-V5 antibody, 1 µg rontalizumab (MA5-41908; Thermo Fisher Scientific), 1 µg sifalimumab (MA5-41904; Thermo Fisher Scientific), or individual plasmas (diluted 1:100) with constant rotation at 4°C. Protein G Sepharose beads (17061801; Cytiva) were added (12.5 µl per sample), and samples were incubated for a further 2 h with constant rotation at 4°C. After washing beads six times with IP buffer (50 mM Tris-HCl, pH 7.5, 150 mM NaCl, 1 mM EDTA, 1% Triton X-100), bead-associated HiBiT-tagged proteins were quantified using the Nano-Glo HiBiT Lytic Reagent Detection system (Promega) according to the manufacturer’s instructions. The reconstituted luciferase activity levels were determined using a PerkinElmer EnVision plate reader (EV2104). The luciferase activity levels were normalized to the values obtained for the anti-V5 antibody conditions, expressed as normalized relative light units.

### BLI

His-tagged IFNAR1 (IF1-H5225; ACROBiosystems), His-tagged IFNAR2 (IF2-H5224; ACROBiosystems), Fc-tagged IFNα1 (IFA-H5258; ACROBiosystems), and IFNω (NBP2-35893; Novus Biologicals) were diluted to the indicated concentrations in assay buffer (0.02% Tween-20, 0.1% BSA in 1x PBS, pH 7.4). All buffers and samples were then arrayed in a Greiner black microplate at a total volume of 200 µl per well, with the layout defined by the respective experimental protocol. Ni-NTA biosensors (18-5101; Sartorius) that had been prehydrated for 10 min in assay buffer prior to each experiment were used for all assays. For initial optimization of protein concentrations (IFNα1, IFNω), the following protocol was used: baseline in assay buffer for 60 s; IFNAR1 (25 nM) or IFNAR2 (100 nM) loading for 600 s; baseline in assay buffer for 180 s; association of the IFN-I under study for 60–120 s; and dissociation in assay buffer for 120 s. For detecting the inhibition of interactions between the IFN-Is and each receptor, rontalizumab (1:50), sifalimumab (1:50), or individual plasma samples (1:10) were diluted in assay buffer containing 50% goat serum (GS) (16210064; Thermo Fisher Scientific), as well as the indicated IFN-I dilutions, and were incubated for 1 h at room temperature with constant shaking at 600 rpm. The following assay protocol was then used: baseline in assay buffer for 60 s; IFNAR1 or IFNAR2 loading for 600 s; blocking in 100% GS for 360 s; baseline in 50% GS in assay buffer for 120 s; association of plasma:IFN-I or antibody:IFN-I mixtures for 120 s. For samples containing donor plasmas, an additional normalization experiment was always performed in parallel using the same assay protocol, but with an association phase condition lacking IFN-I. This allowed the control of plasma-specific associations with the loaded Ni-NTA sensors during the association phase. All BLI assays were performed with an Octet R2 (Sartorius) using a standard kinetics acquisition rate (5.0 Hz). The values from each association condition were normalized to mock association values and plotted, and curves were smoothened in GraphPad Prism 10 software (20 neighbors on each size, second-order polynomial smoothing).

### simIFN-based IFN-I competition assays

20 ml of supernatant from HEK293T cells transfected with simIFN-Is (or mock as control) was harvested and centrifuged for 5 min at 1,000 *g* to pellet cell debris. The soluble supernatant fractions were then concentrated fivefold using protein concentrators PES, 3k MWCO (88525; Pierce) according to the manufacturer’s instructions. Plasma samples were diluted into concentrated supernatants as indicated, and incubated for 1 h at room temperature with constant shaking at 600 rpm. Plasma dilutions were then mixed 1:1 (volume) with 1 ng/ml IFNα2 or IFNω diluted in Opti-MEM containing EnduRen (1:10,000) and were further incubated for 1 h at room temperature with constant shaking at 600 rpm. Supernatant:plasma:IFN-I mixtures were then used to stimulate AIR cells for 24 h, and Renilla luciferase activity was subsequently determined using a PerkinElmer EnVision plate reader (EV2104). Alternatively, supernatant:plasma:IFNα2 mixtures (without EnduRen) were used to pretreat A549 cells for 16 h. Pretreated cells were then inoculated for 1 h at 37°C with H5N1-GFP (MOI 0.1 PFU/cell), PIV2-GFP (MOI 0.1 FFU/cell), PIV5-GFP (MOI 0.01 FFU/cell), MeV-GFP (MOI 0.03 FFU/cell), or RSV-GFP (MOI 0.1 TCID_50_/cell). The medium was then replaced with DMEM supplemented with 100 U/ml penicillin and streptomycin, 0.3% BSA, 0.1% FBS, 0.2 M HEPES, and 1 µg/ml TPCK trypsin for H5N1-GFP infections, or with DMEM supplemented with 2% FBS and 100 U/ml penicillin and streptomycin for PIV2-GFP, PIV5-GFP, MeV-GFP, and RSV-GFP inoculated cells. GFP expression was then monitored every 4 h over the course of 3 days using an IncuCyte S3 Live-Cell analysis system (Sartorius). Total green integrated intensity (Green Calibrated Units × µm^2^/image) values were derived and used to calculate the area under the curve.

### simIFN-based autoAb depletion assays

HEK293T cells were cotransfected with plasmids expressing the ER-targeted BirA biotin ligase (pDisplay-BirA-ER, #20856; Addgene, a kind gift from Alice Ting [Stanford University, Stanford, CA, USA] [Howarth et al., 2008]) and the C-terminally Avi-tagged (Choi-Rhee et al., 2004) simIFNα (or HA-GFP as control). At 24 h after transfection, cells were lysed and centrifuged for 5 min at 1,000 *g* to pellet cell debris. The soluble fractions were then incubated with streptavidin-coated magnetic beads (Dynabeads MyOne Streptavidin T1, 65601; Thermo Fisher Scientific) overnight at 4°C with constant rotation at 10 rpm in order to capture the biotinylated simIFNα. Beads were then washed three times with PBS prior to incubation with individual plasma samples diluted 1:100 in Opti-MEM for 2 h at room temperature with constant rotation at 10 rpm. The beads were then magnetically separated from supernatants, and supernatants (containing the plasma dilutions) were re-incubated with a fresh set of streptavidin-coated magnetic beads coupled with the simIFNα (or control). After a further incubation for 2 h at room temperature with constant rotation at 10 rpm, beads and supernatants were again magnetically separated and the resulting supernatants (containing the plasma dilutions) were used for detection of remaining anti-IFNα IgG and other specific IgGs as described above. IFNα neutralization activity was also determined as described above by spiking IFNα2 (1 or 10 ng/ml) and EnduRen (1:10,000 final dilution) into the resulting supernatants and assessing function in AIR cells.

### Protein structures

Protein structures from the RCSB Protein Data Bank (PDB) were analyzed using PyMOL version 3.0.2. The following PDB structures were used: human IFNα2:IFNAR ternary complex (PDB: 3SE3 [Thomas et al., 2011]), rontalizumab Fab bound to IFNα2 (PDB: 4Z5R [Maurer et al., 2015]), sifalimumab Fab bound to IFNα2 (PDB: 4YPG [Oganesyan et al., 2015]), IFNω structure from human IFNω:IFNAR ternary complex (PDB: 3SE4 [Thomas et al., 2011]).

### Statistics

Statistical analyses were performed using the GraphPad Prism 10 software. Comparisons were made using the tests indicated in each figure caption. Significance is denoted as follows: ns, not significant; *P < 0.05; **P < 0.01; ***P < 0.001; ****P < 0.0001.

### Online supplemental material

Fig. S1, Fig. S2, and Fig. S3 contain validation and original data supporting the quantifications reported in Fig. 1 and Fig. 2, as well as relevant sequence alignments and annotated protein structures. Fig. S4 contains IFNα2 protein structures with annotated anti-IFNα autoAb footprints and experimental workflows related to Fig. 7. Fig. S5 contains experimental workflows and validation data related to Fig. 9. Source data files for all western blots are provided.

## Supplementary Material

SourceData F1 is the source file for Fig. 1.

SourceData F7 is the source file for Fig. 7.

SourceData F8 is the source file for Fig. 8.

SourceData FS1 is the source file for Fig. S1.

SourceData FS2 is the source file for Fig. S2.

SourceData FS3 is the source file for Fig. S3.

## Data Availability

Data acquired specifically for this study are available within the article itself and its supplementary materials.
