# Supplementary figures and images for: Type I interferon autoantibody footprints reveal neutralizing mechanisms and allow inhibitory decoy design
Source: J Exp Med. 2025 Mar 20;222(6):e20242039. doi: 10.1084/jem.20242039 (PMC11924951; doi:10.1084/jem.20242039)

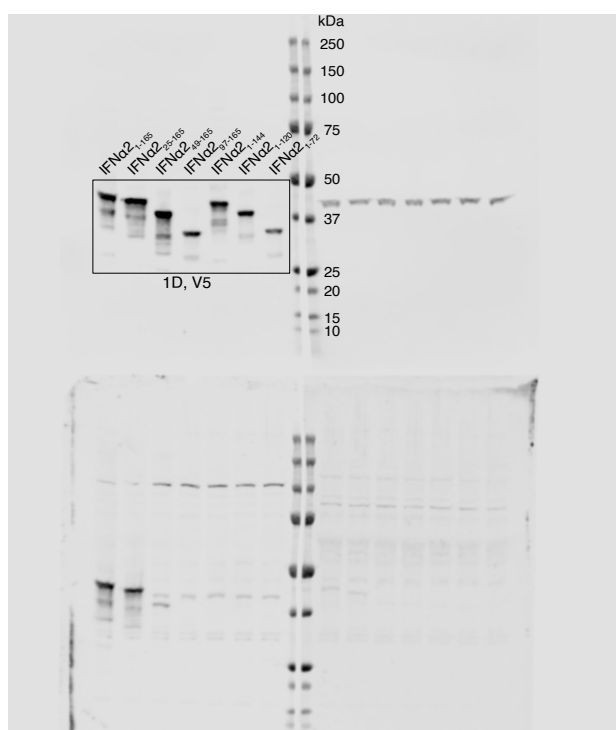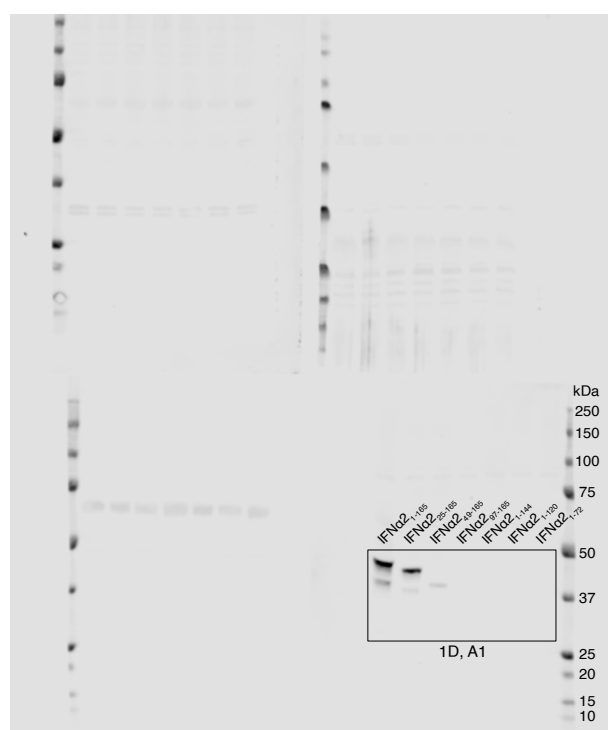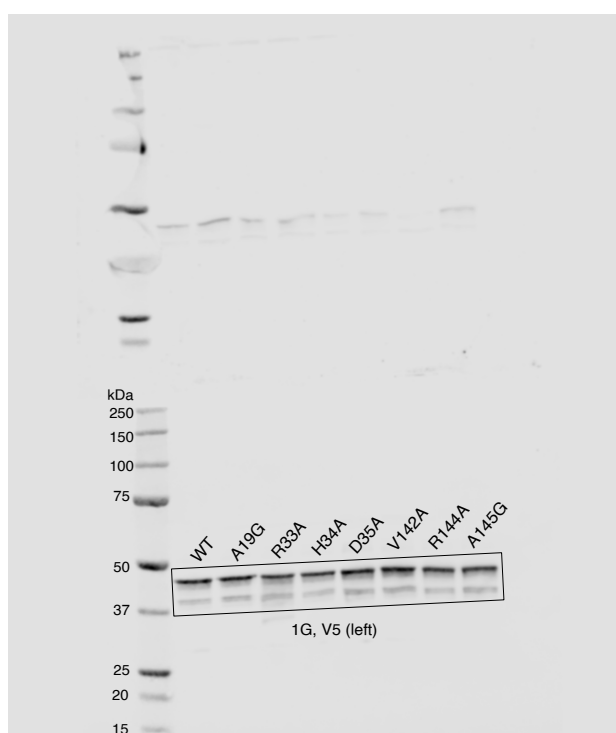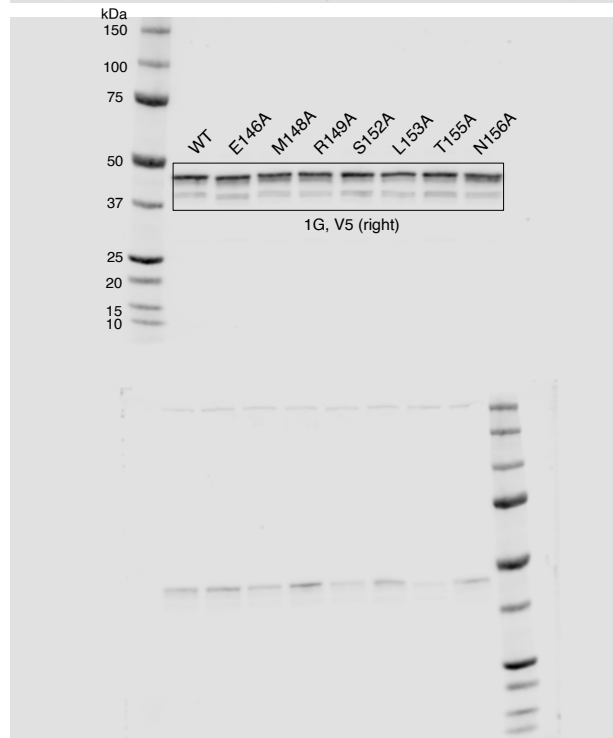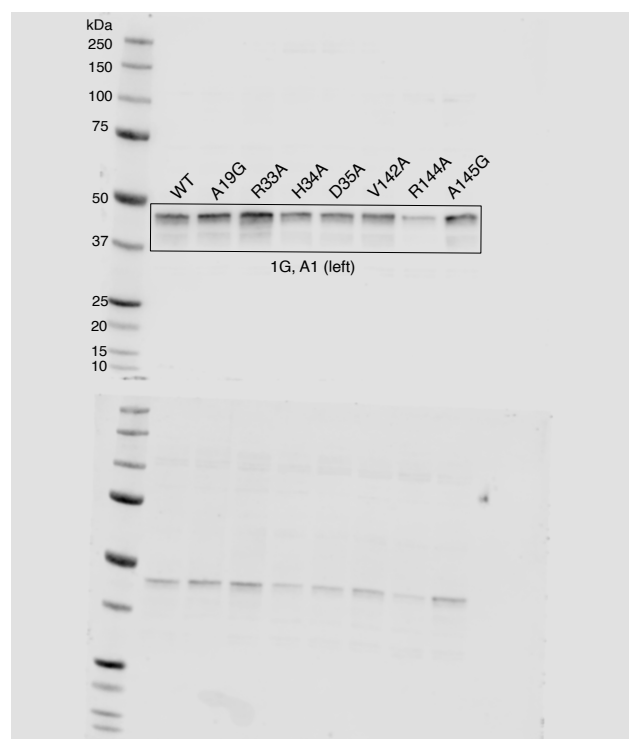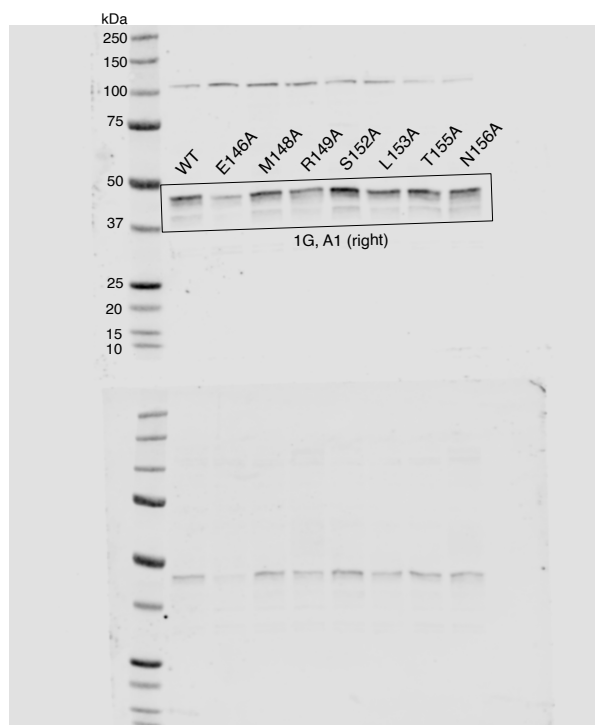

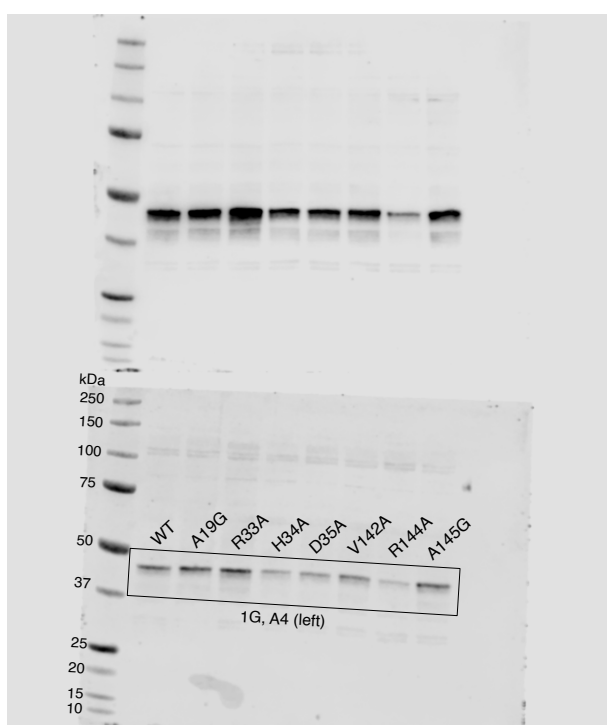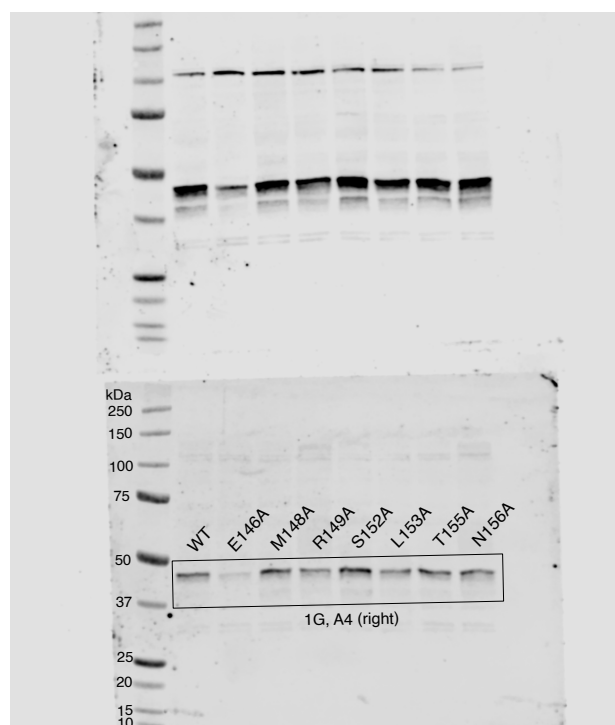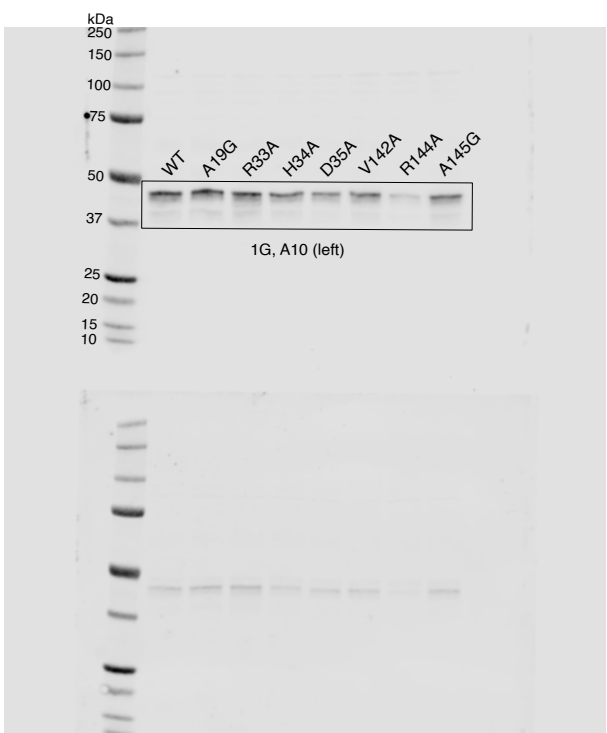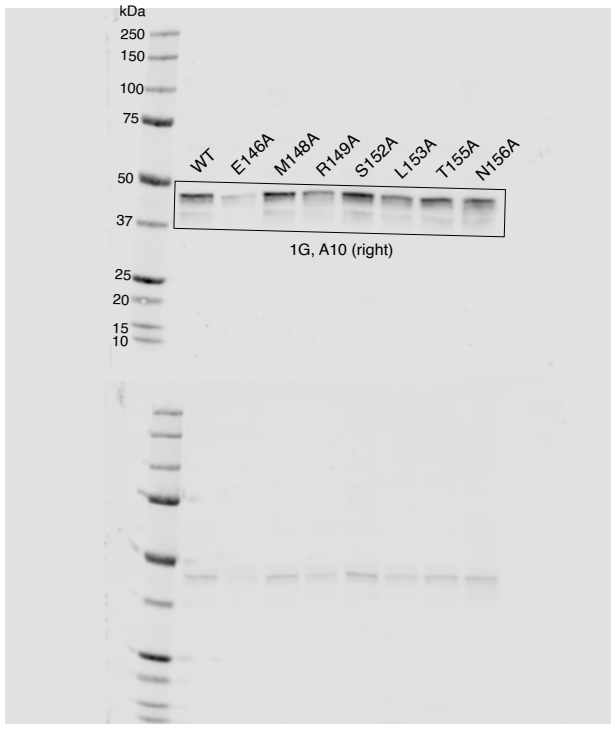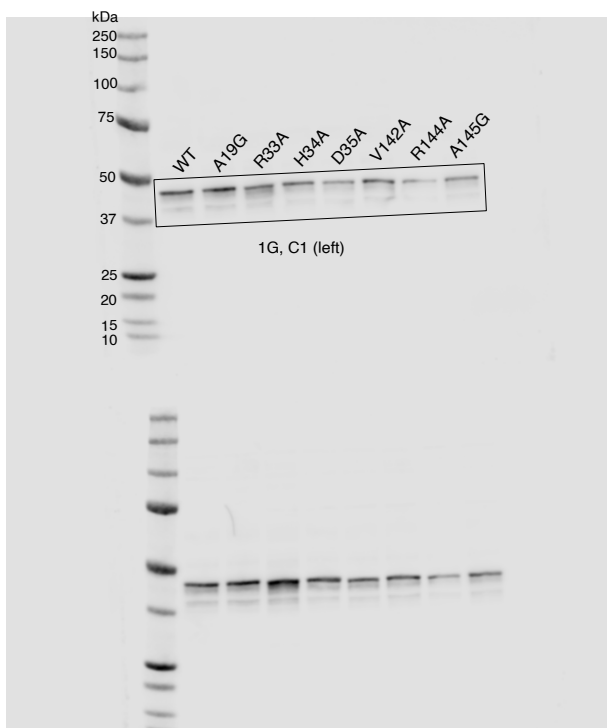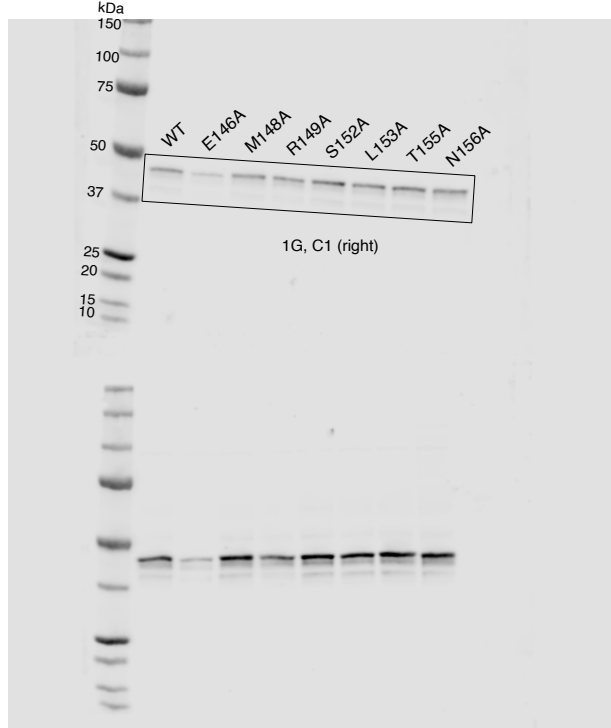

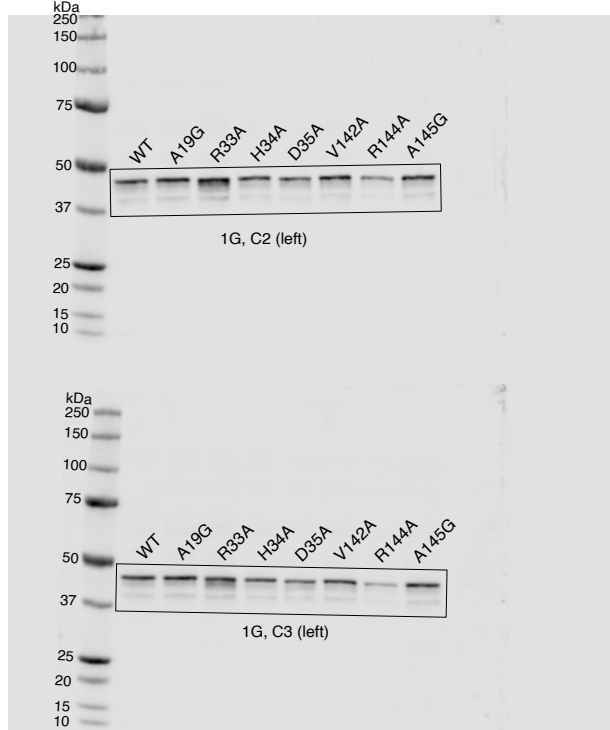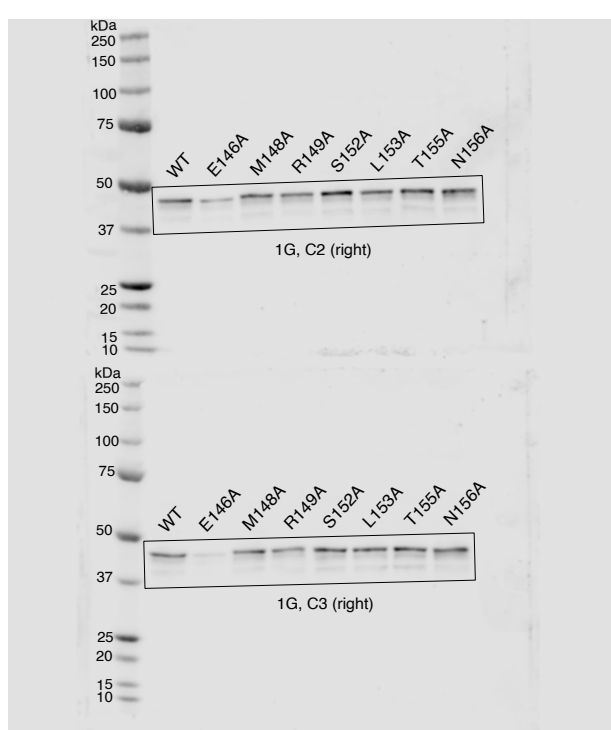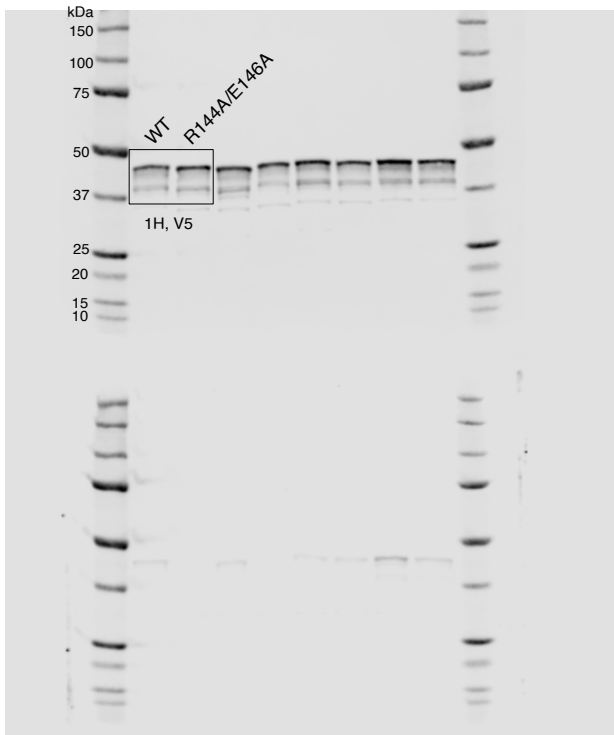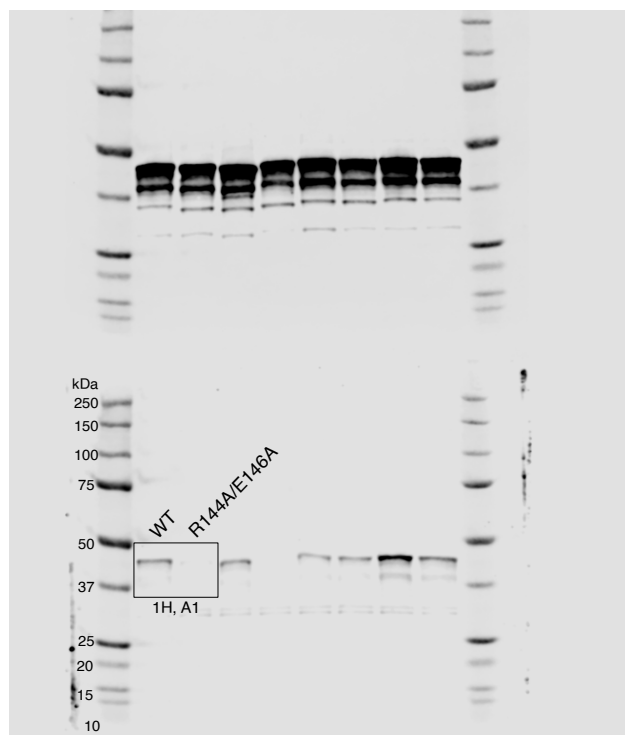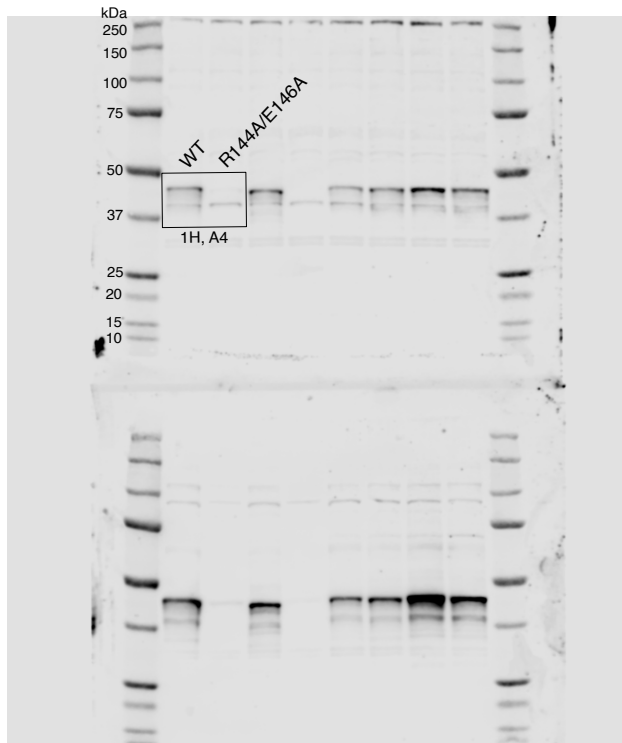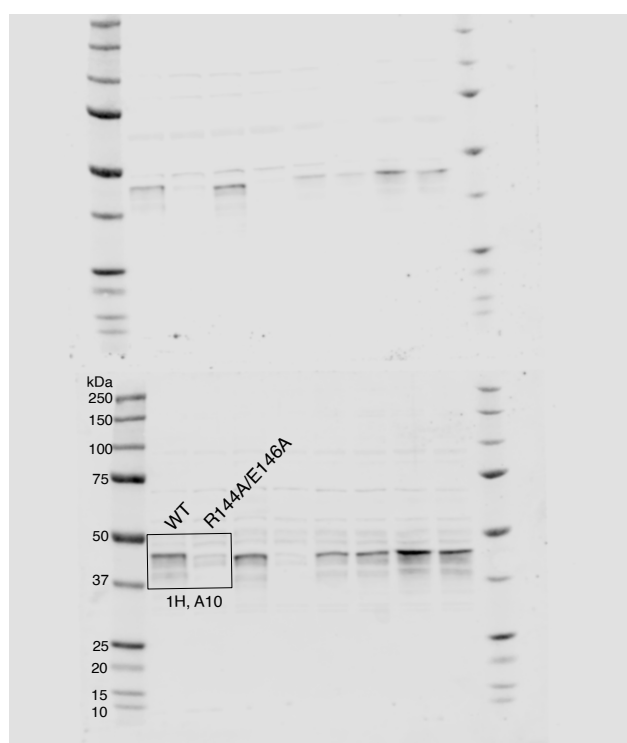

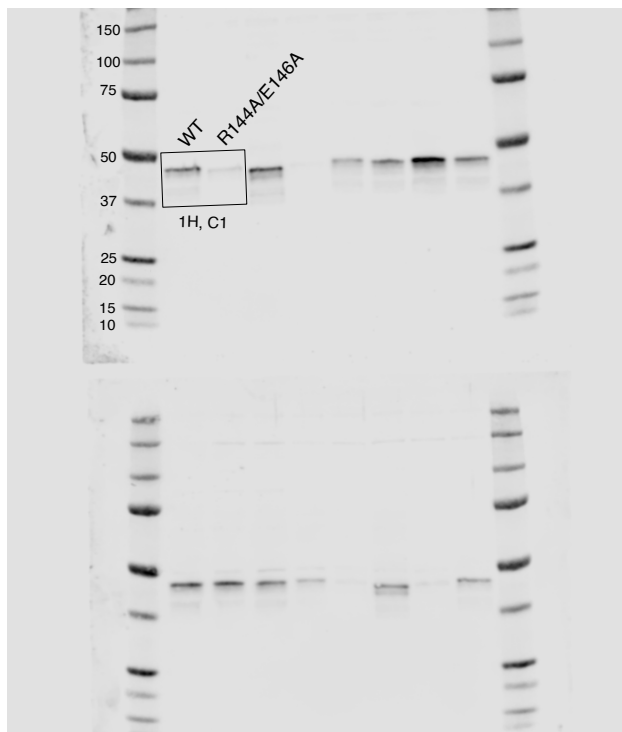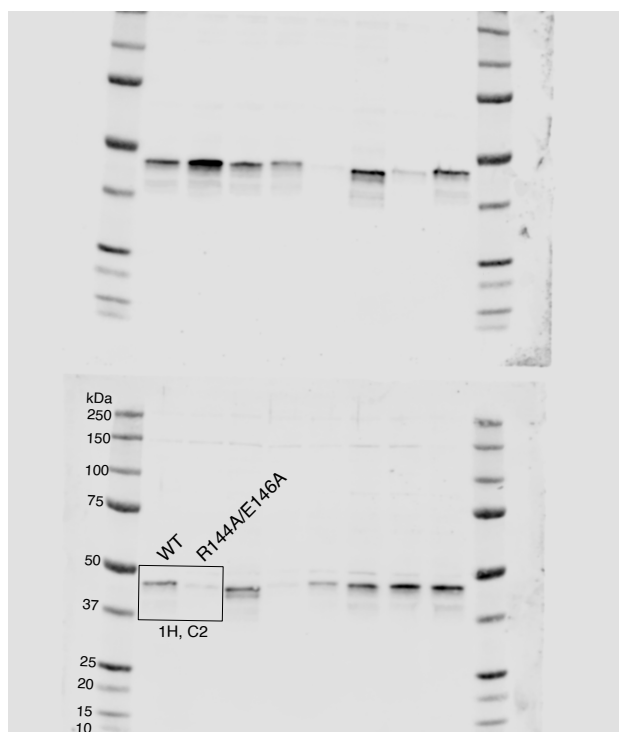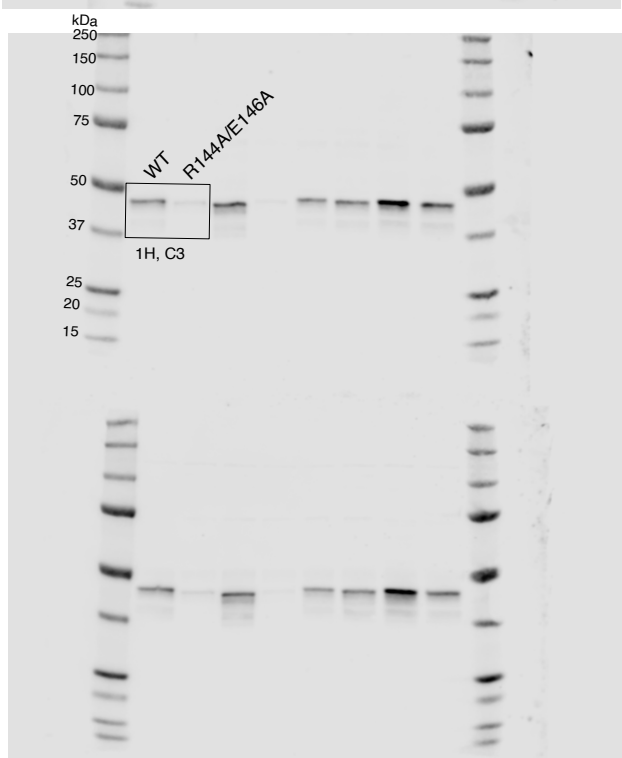

Supplement: SourceData F1 — is the source file for Fig. 1. [file jem_20242039_sourcedataf1.pdf]

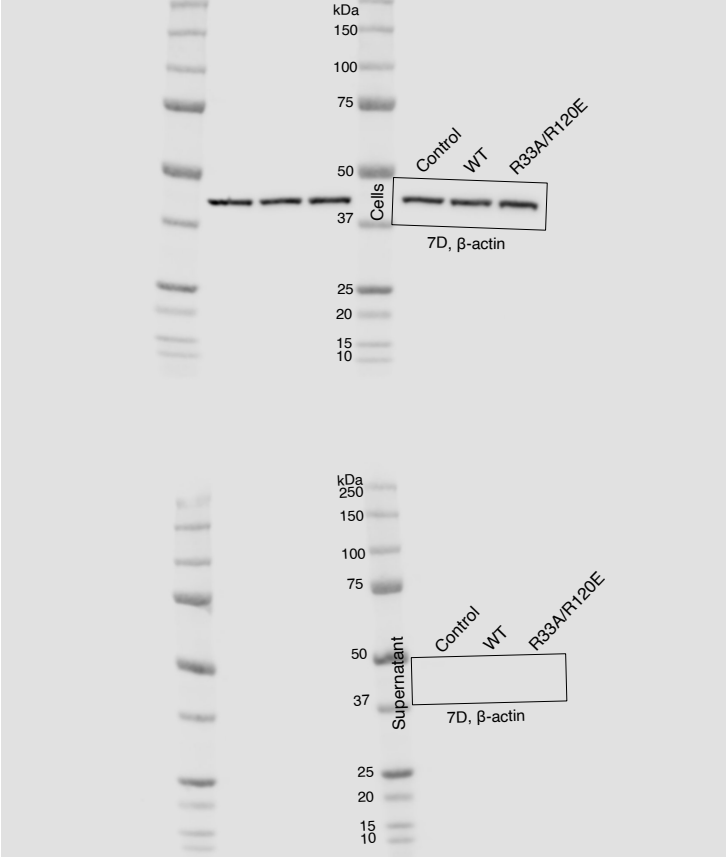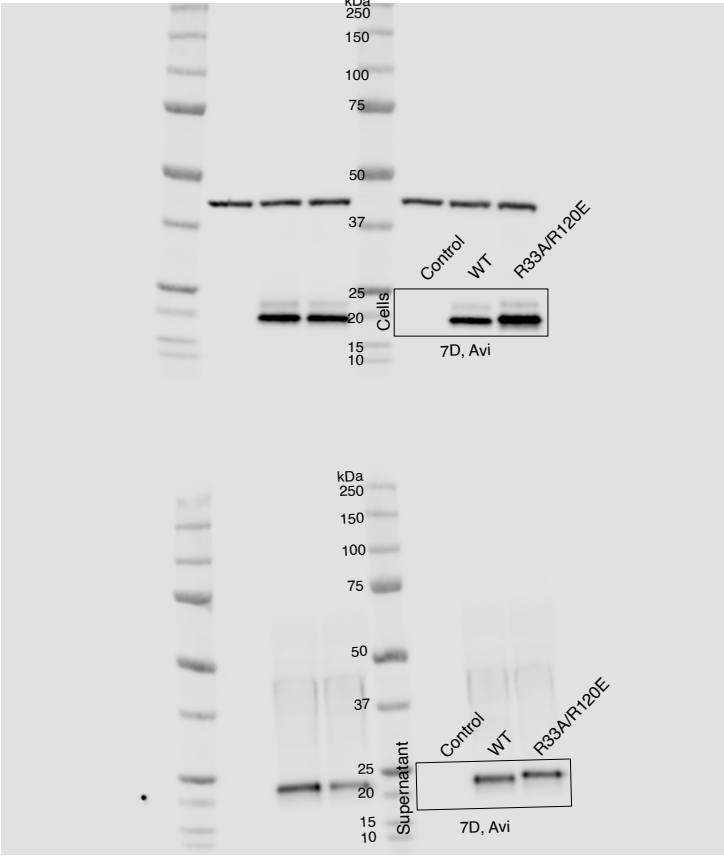

Supplement: SourceData F7 — is the source file for Fig. 7. [file jem_20242039_sourcedataf7.pdf]

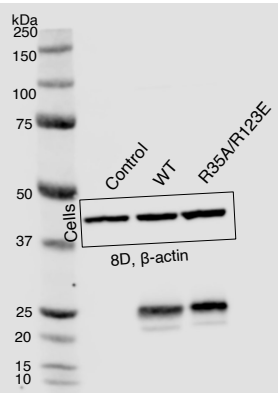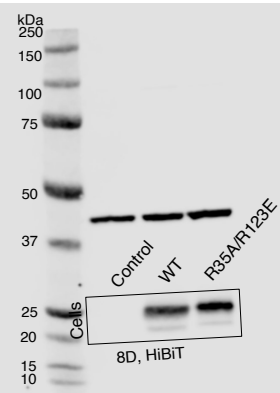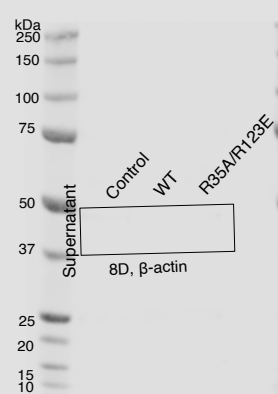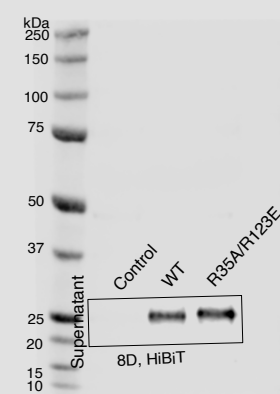

Supplement: SourceData F8 — is the source file for Fig. 8. [file jem_20242039_sourcedataf8.pdf]

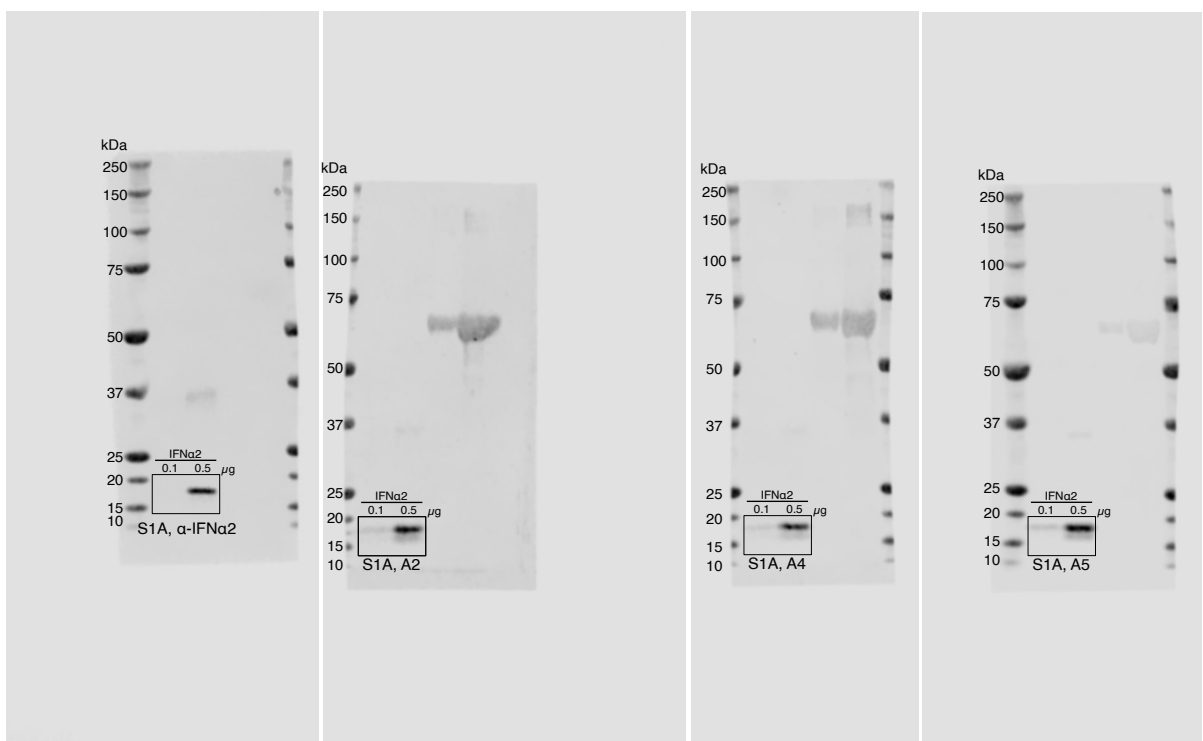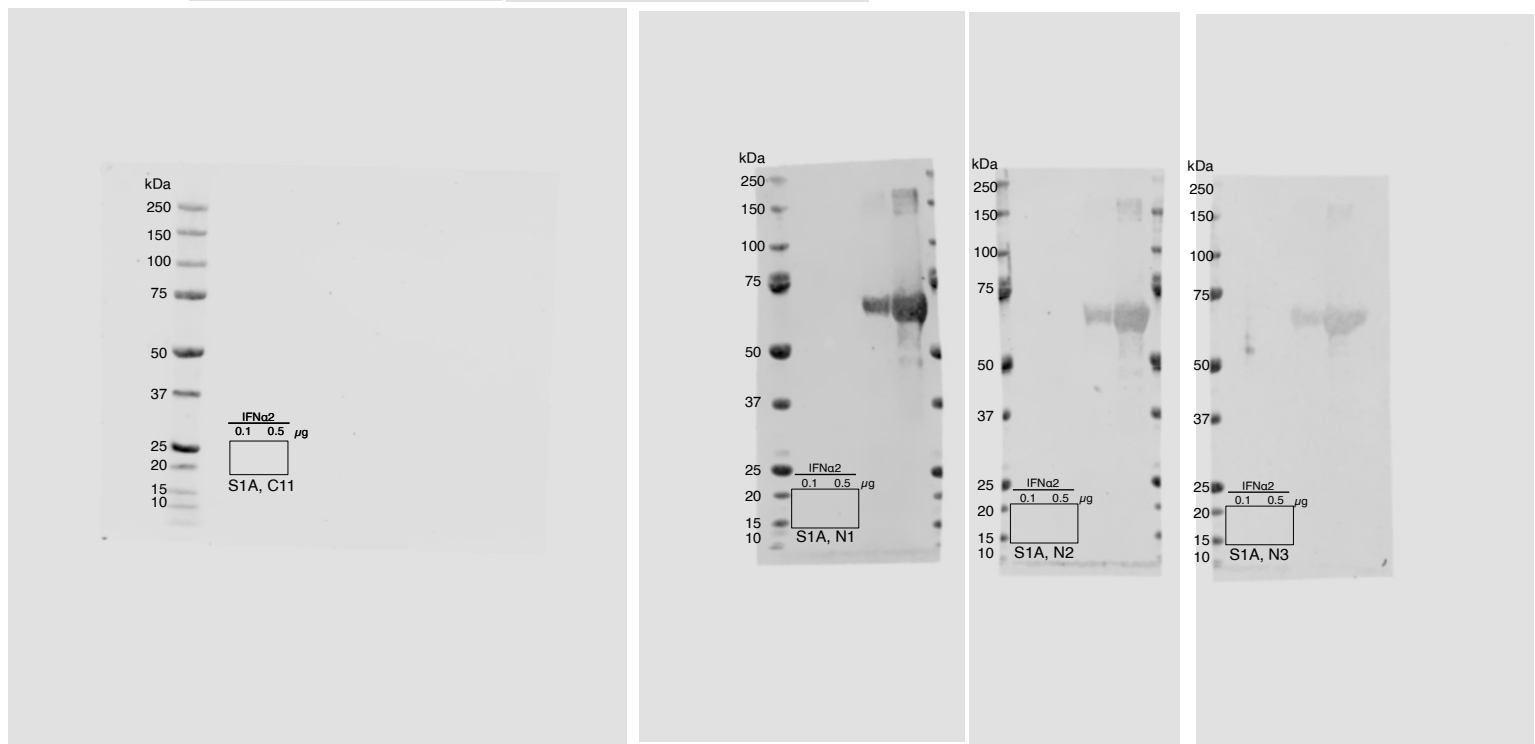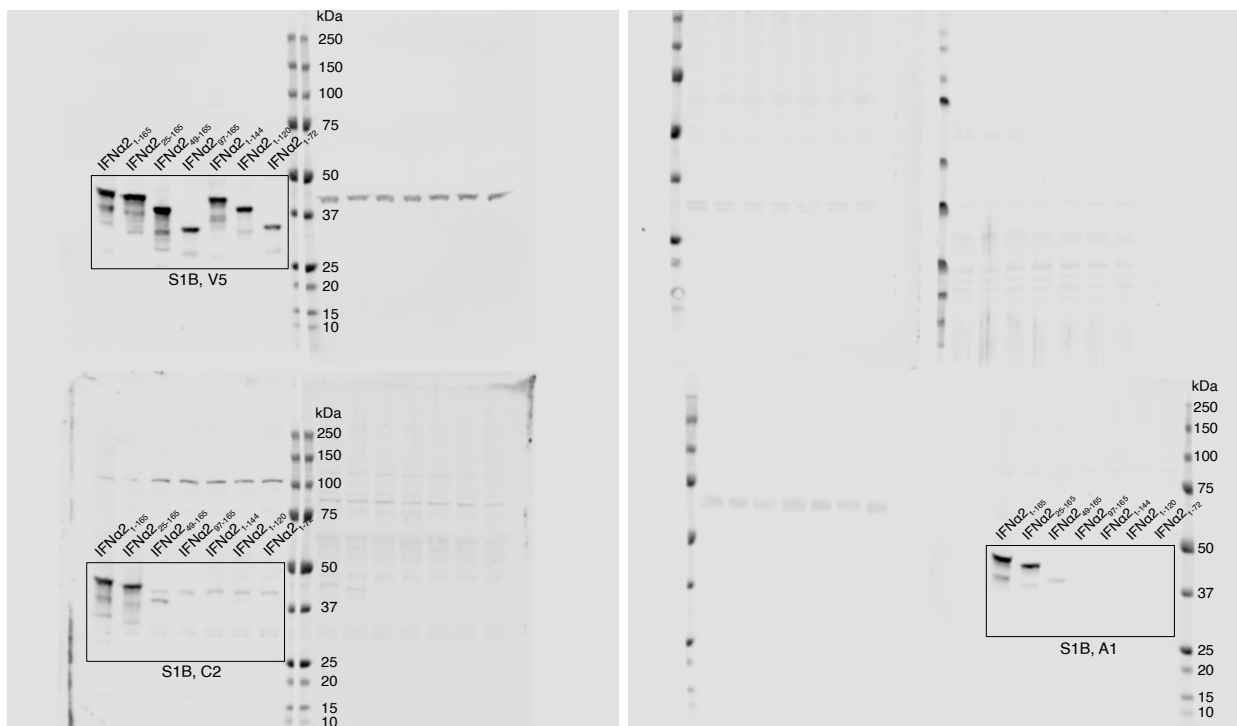

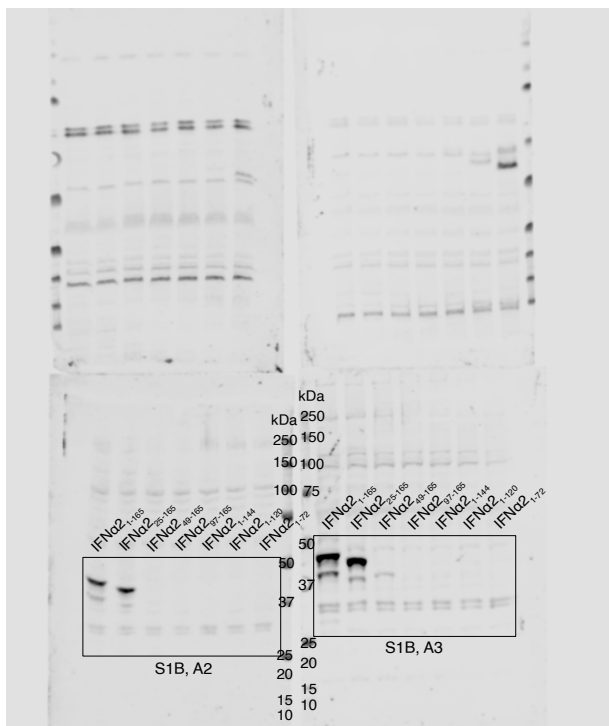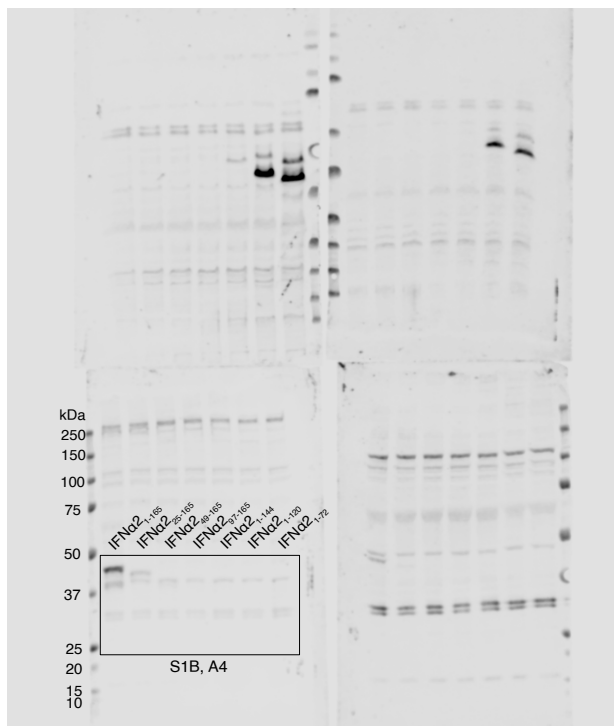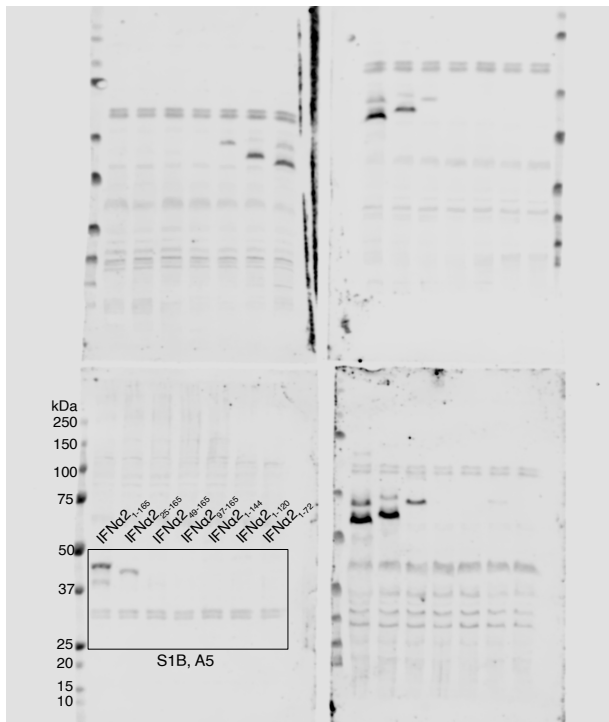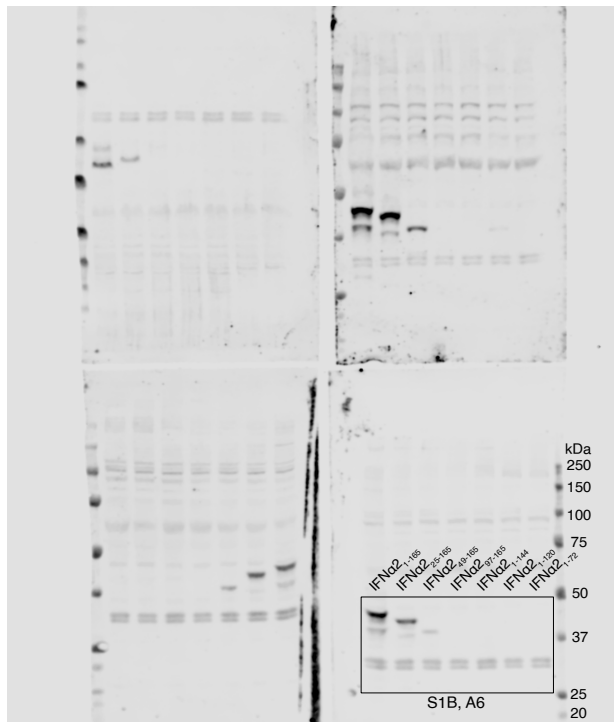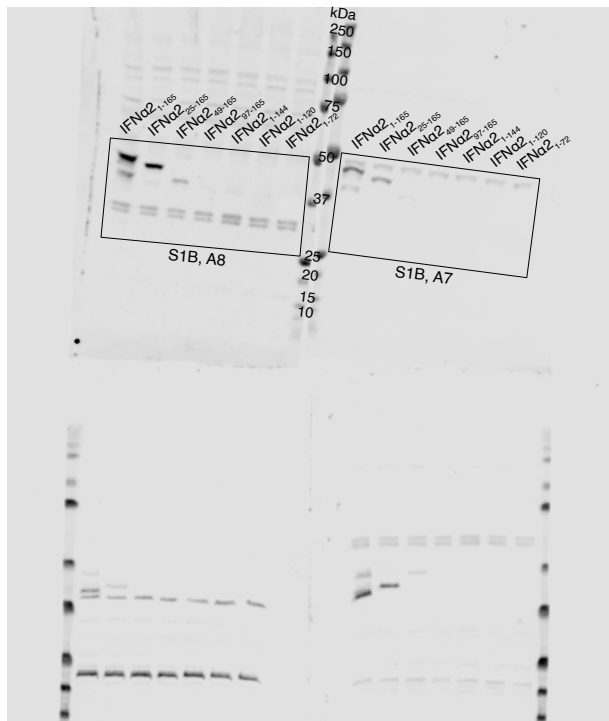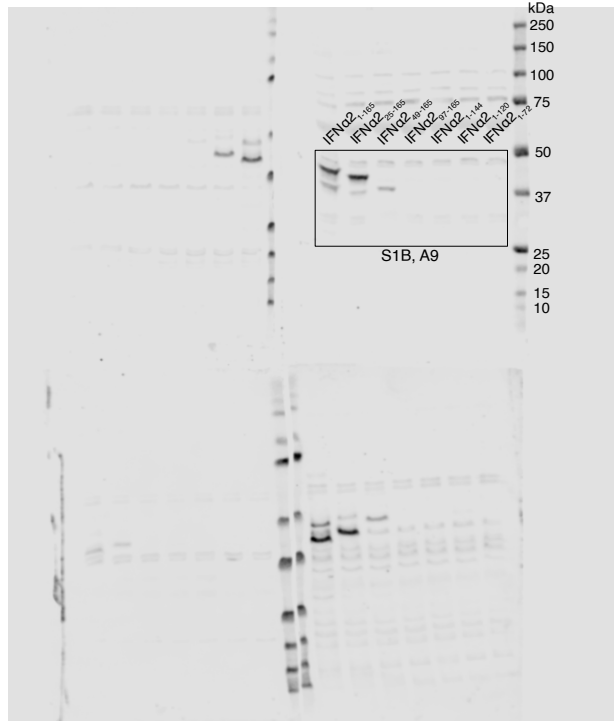

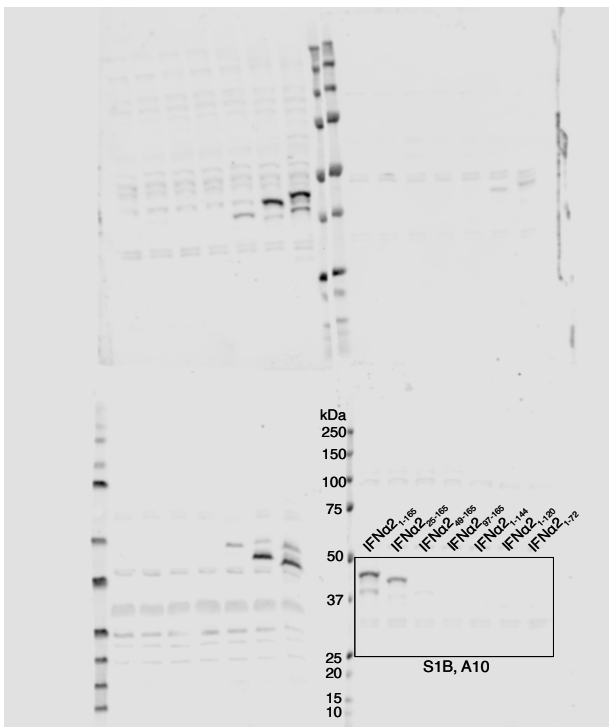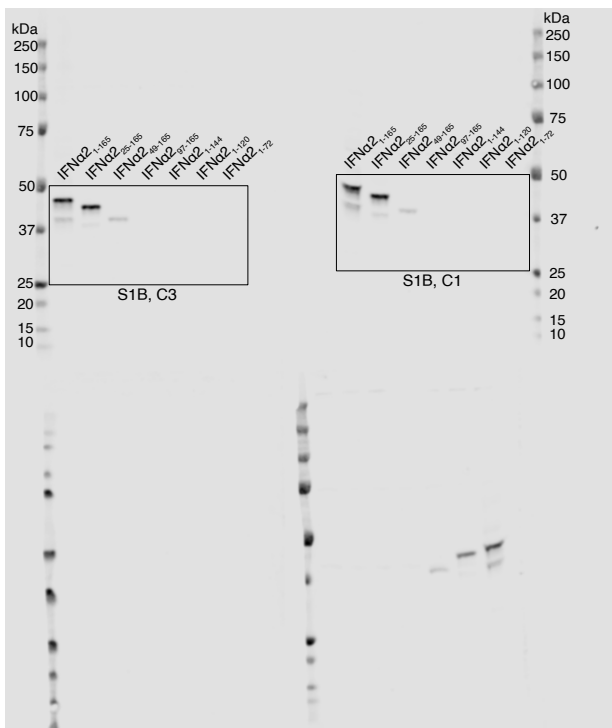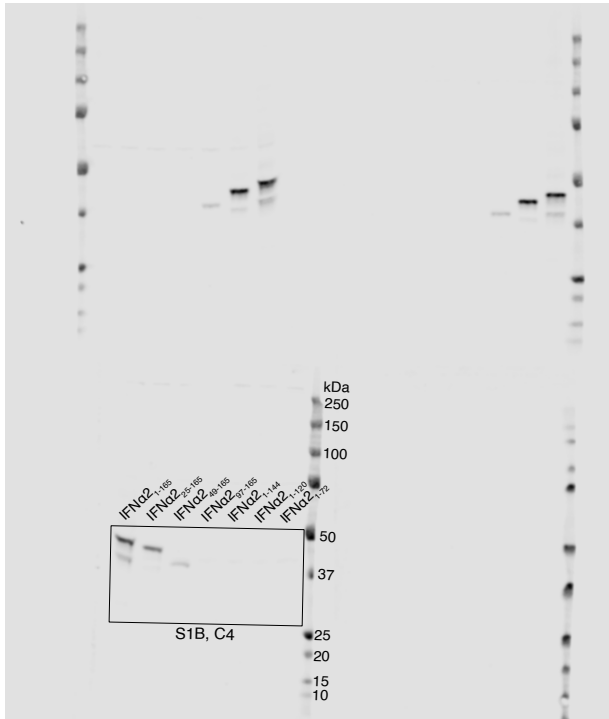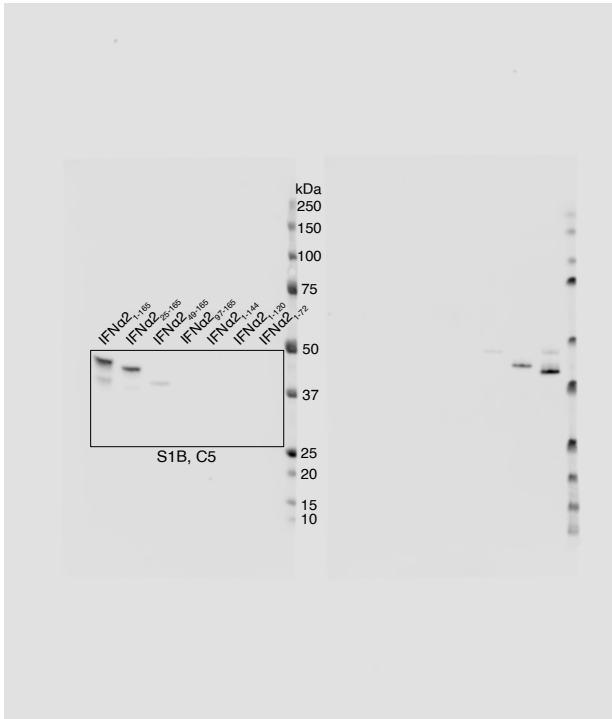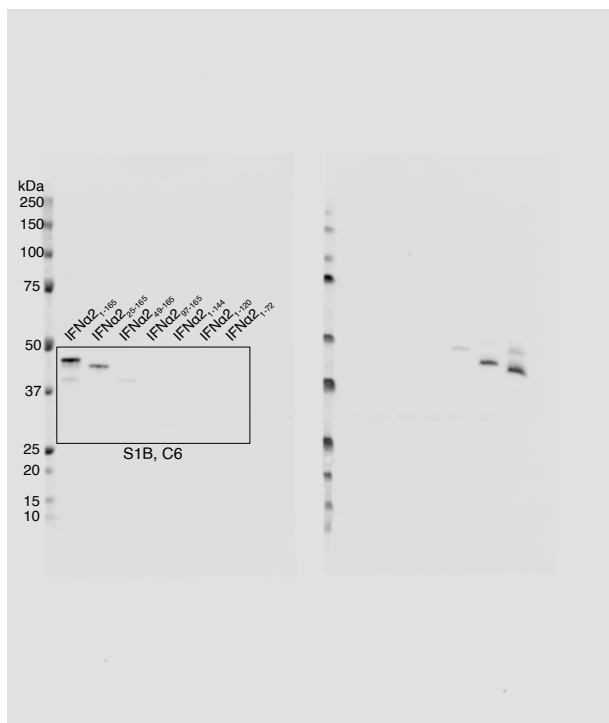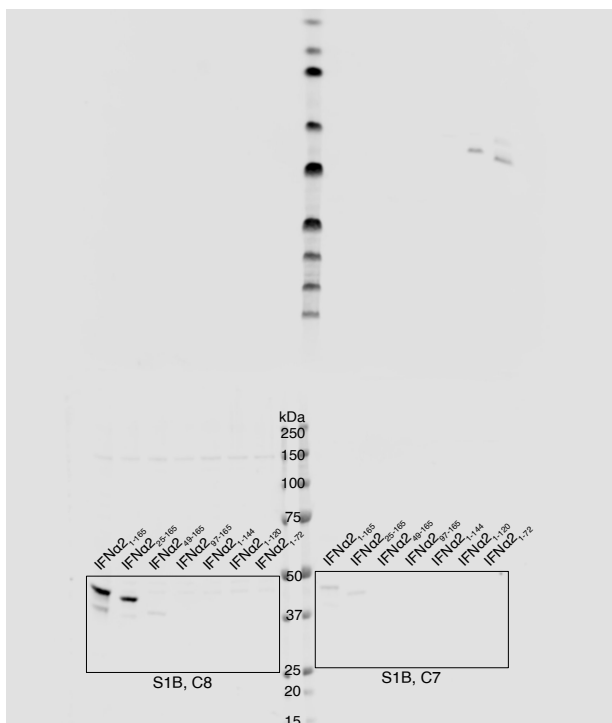

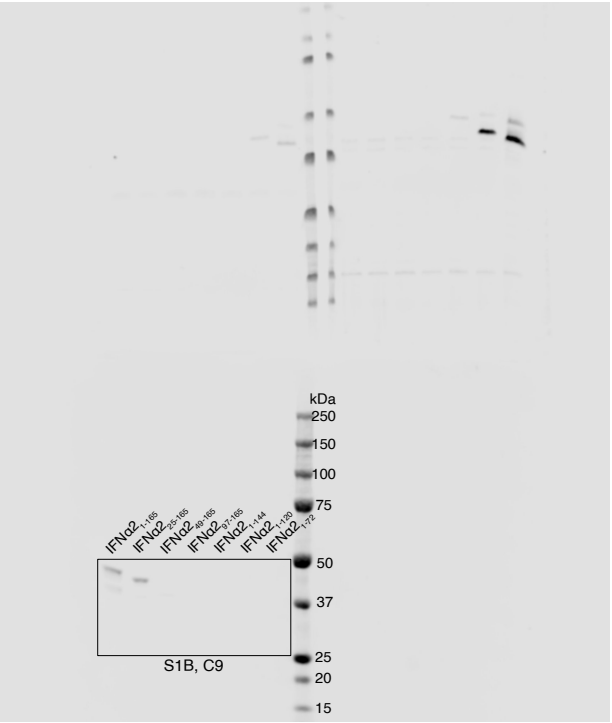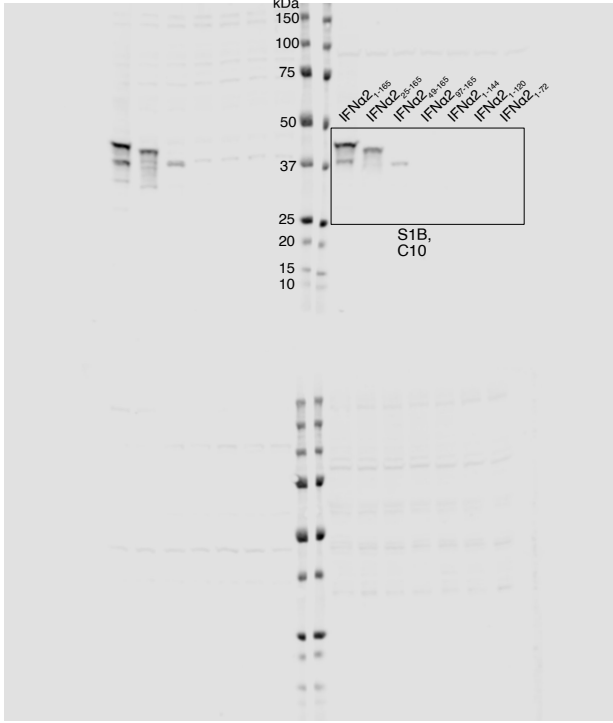

Supplement: SourceData FS1 — is the source file for Fig. S1. [file jem_20242039_sourcedatafs1.pdf]

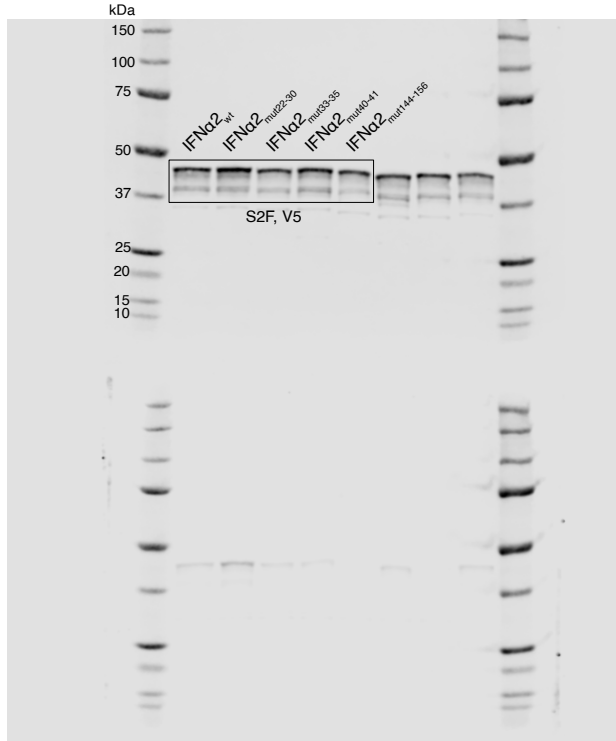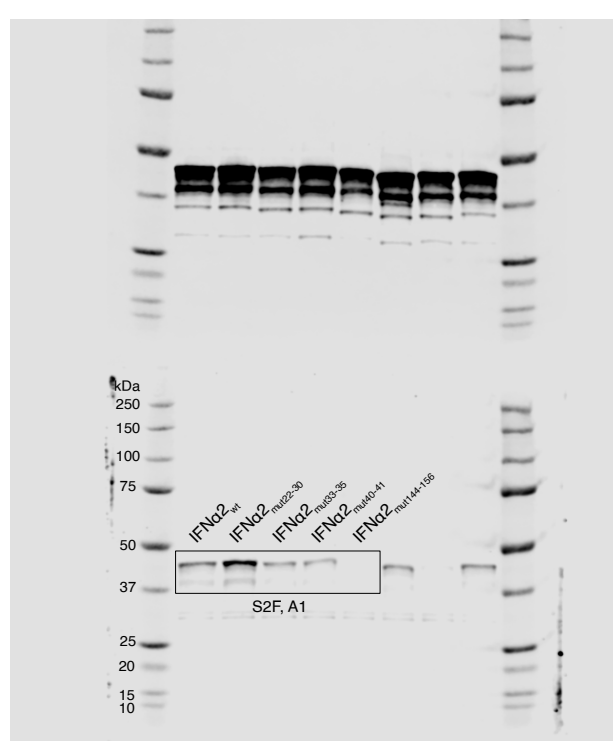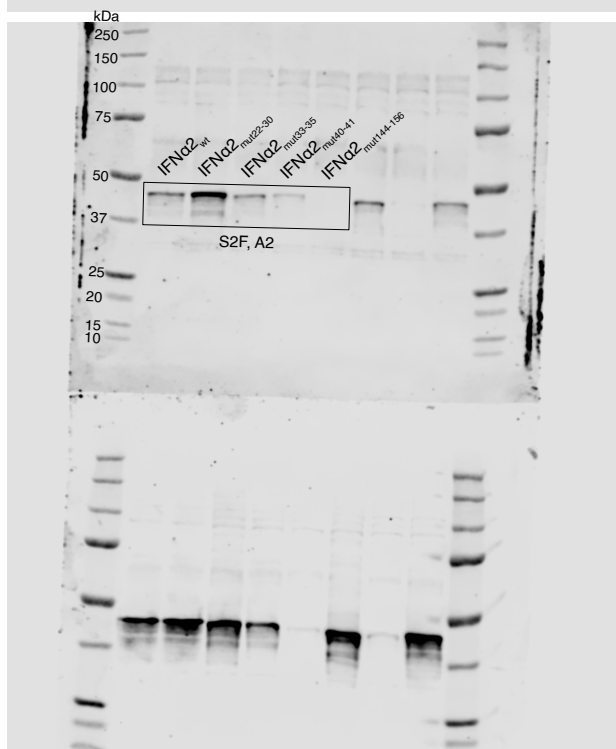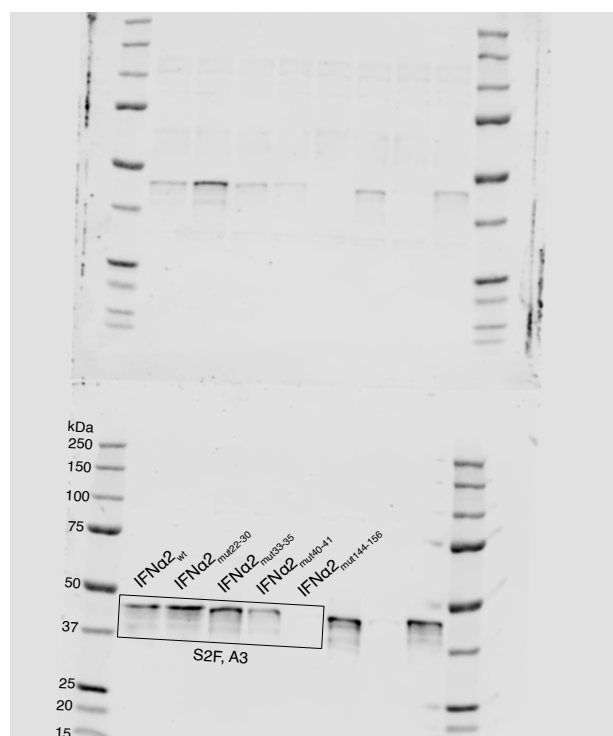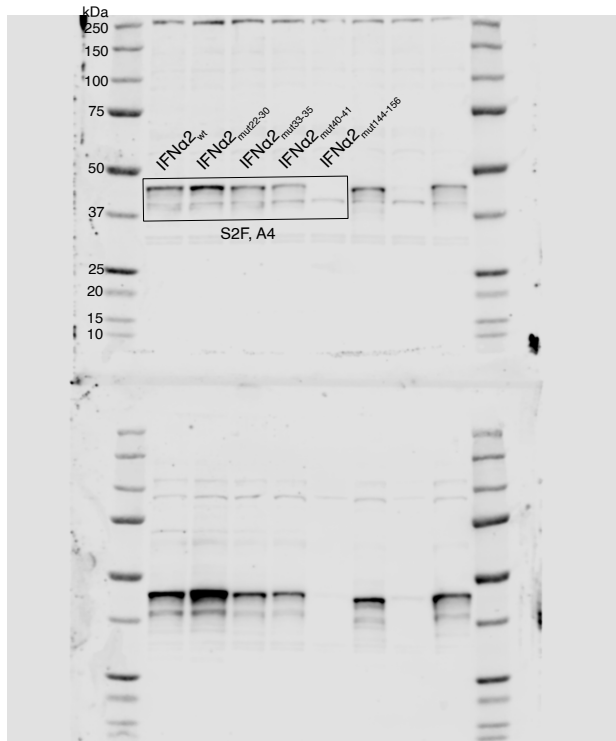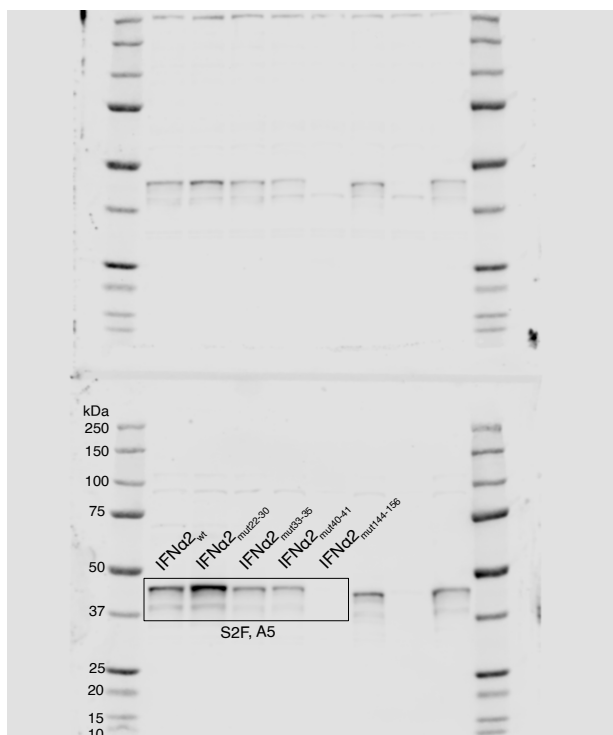

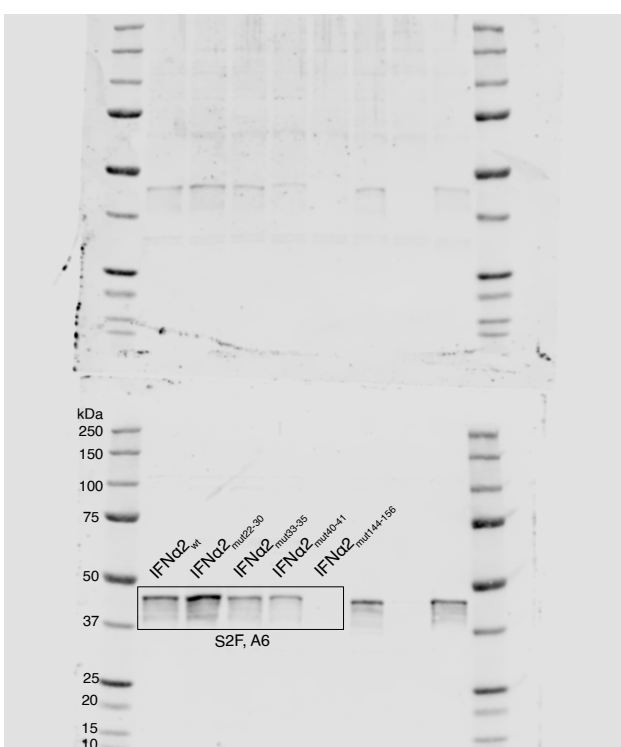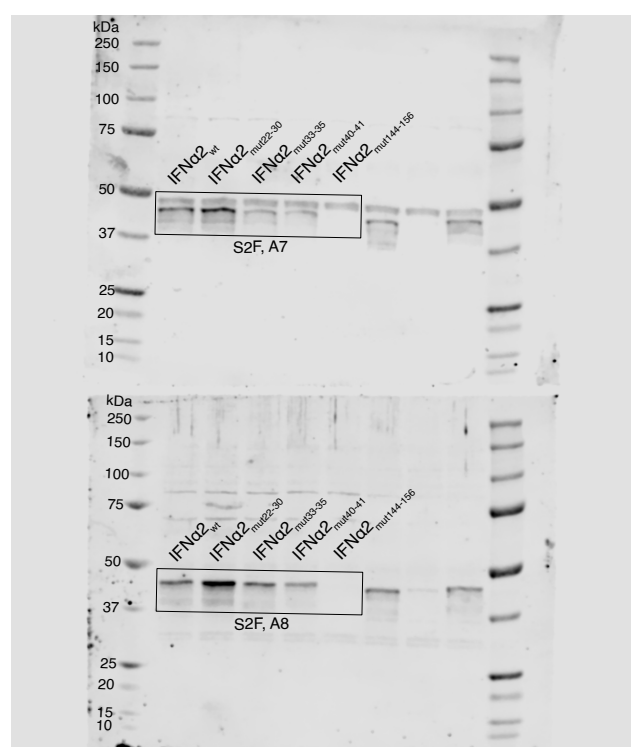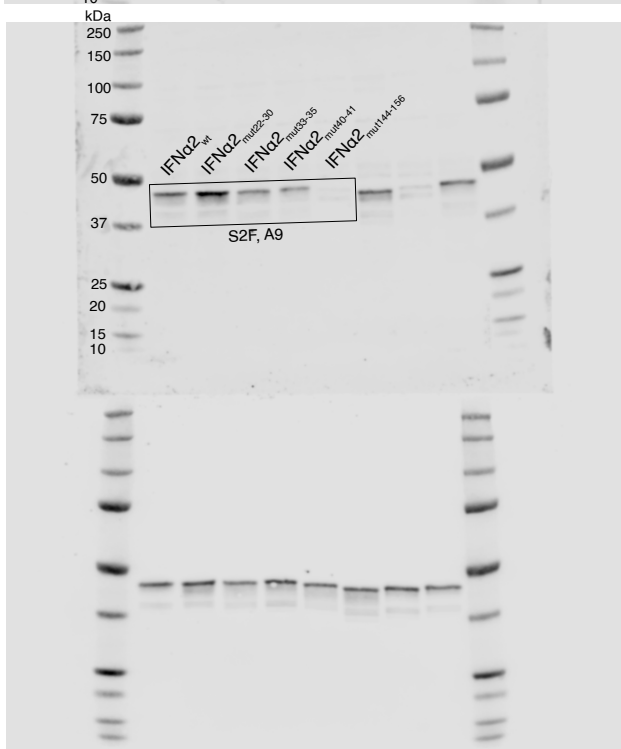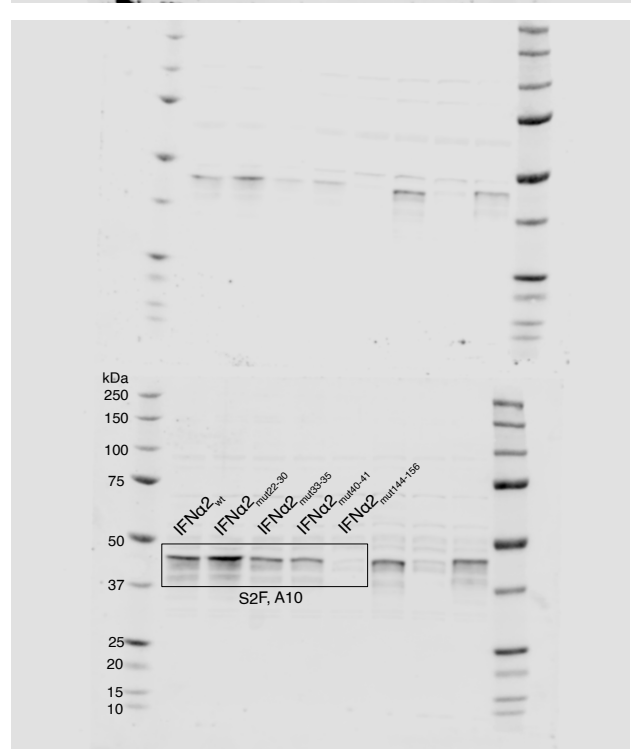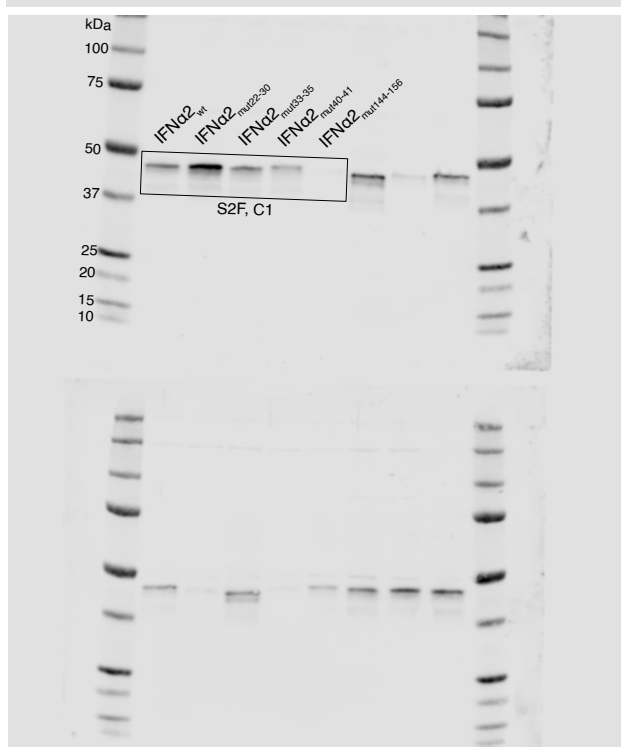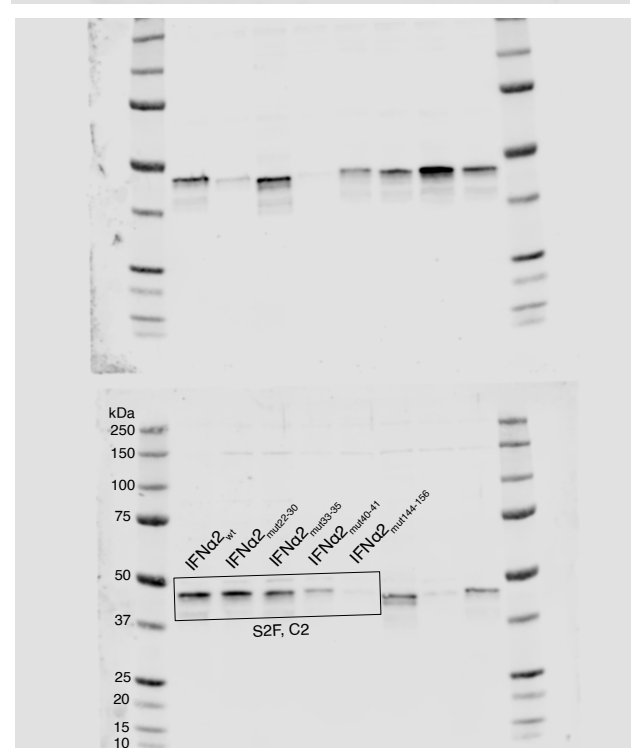

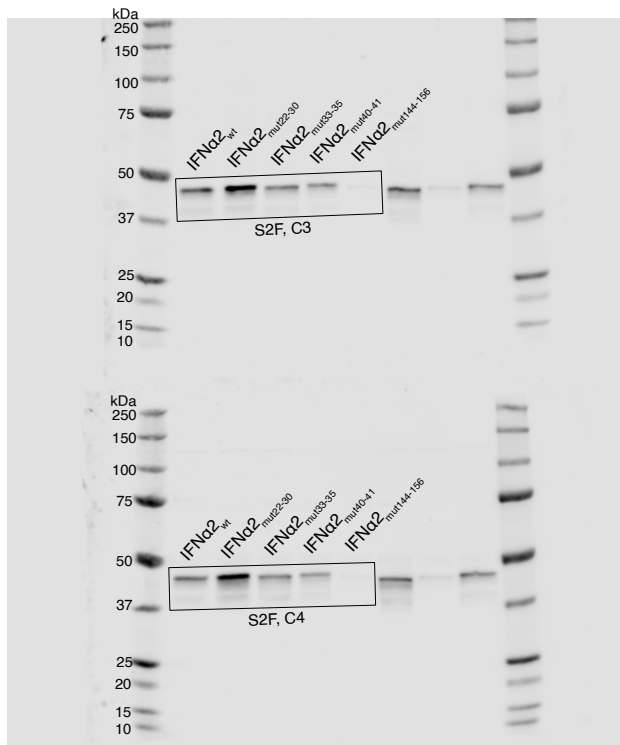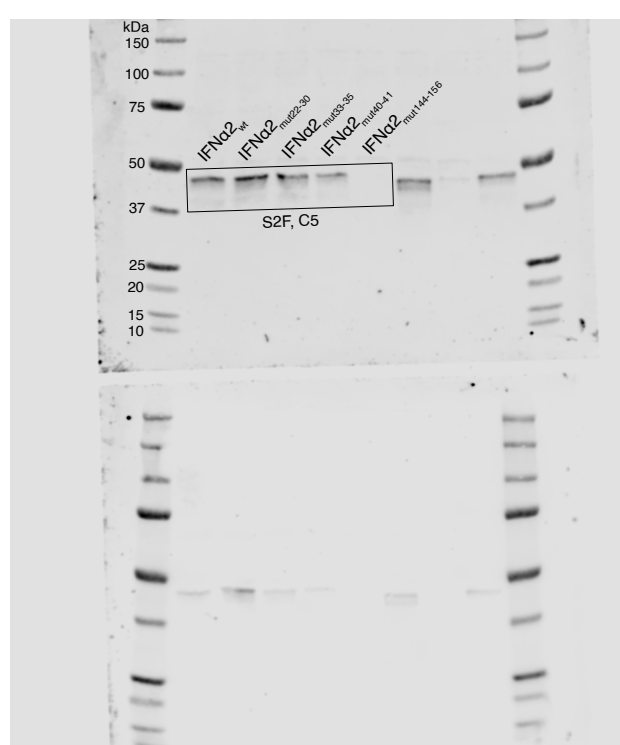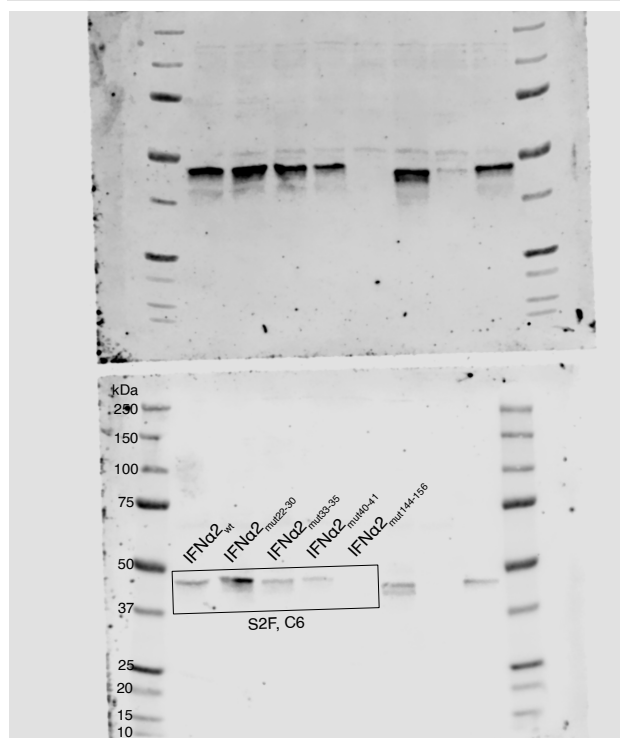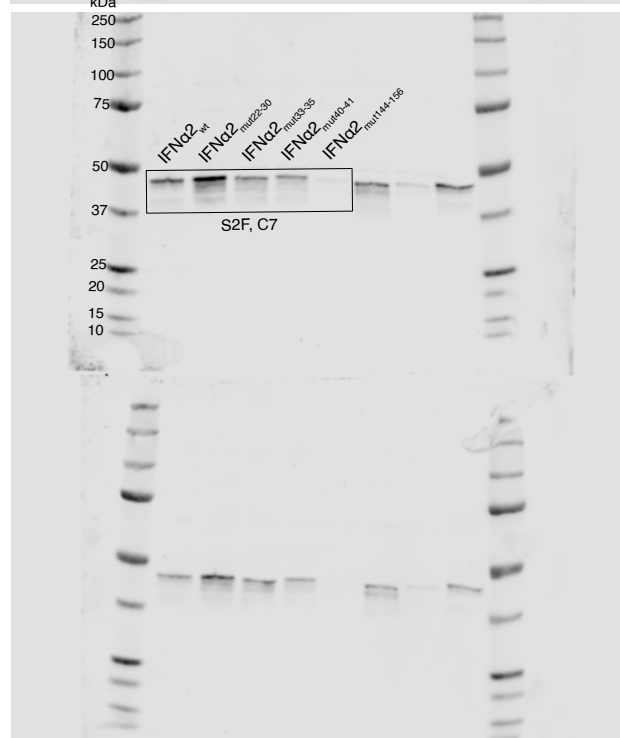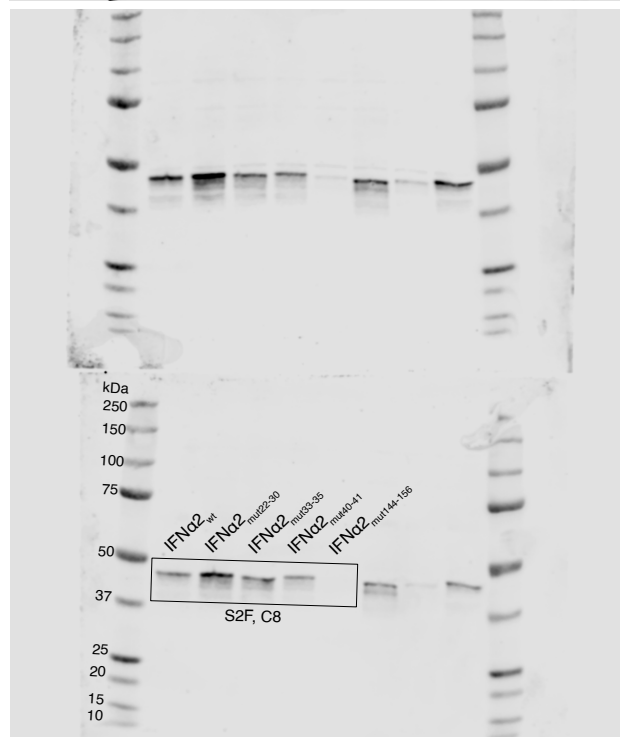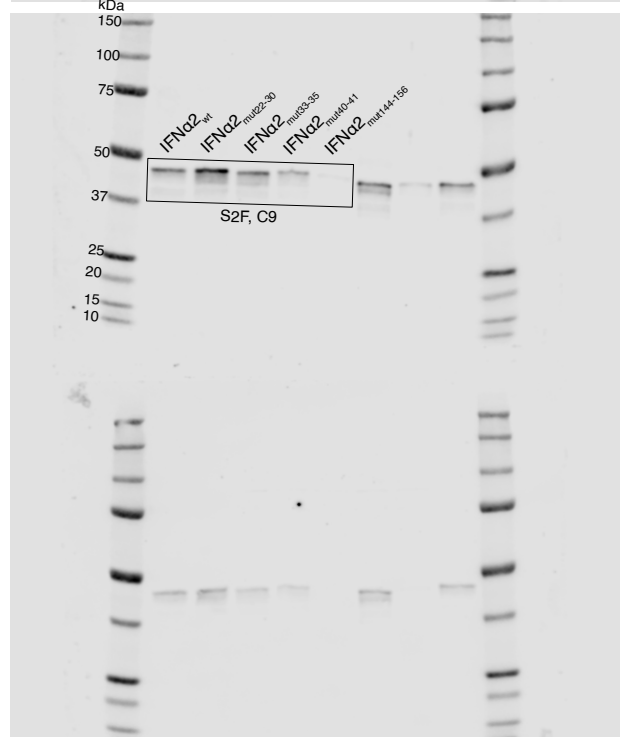

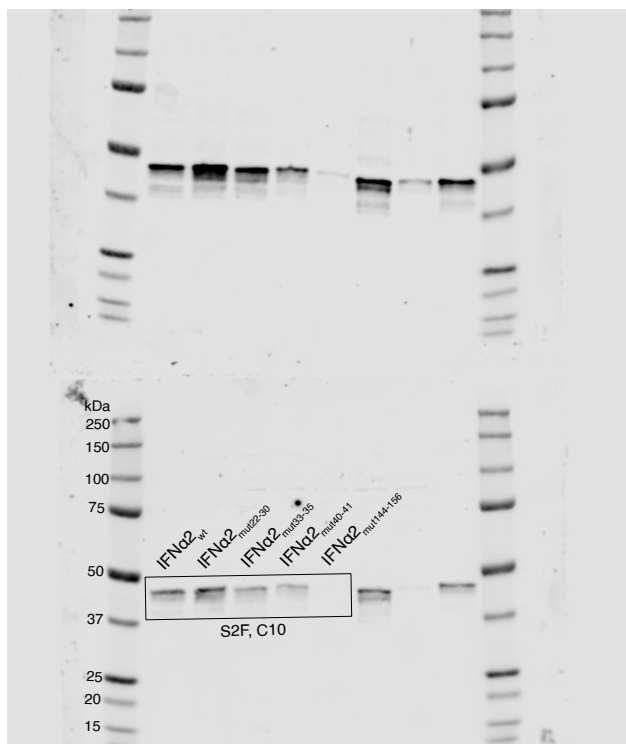

Supplement: SourceData FS2 — is the source file for Fig. S2. [file jem_20242039_sourcedatafs2.pdf]

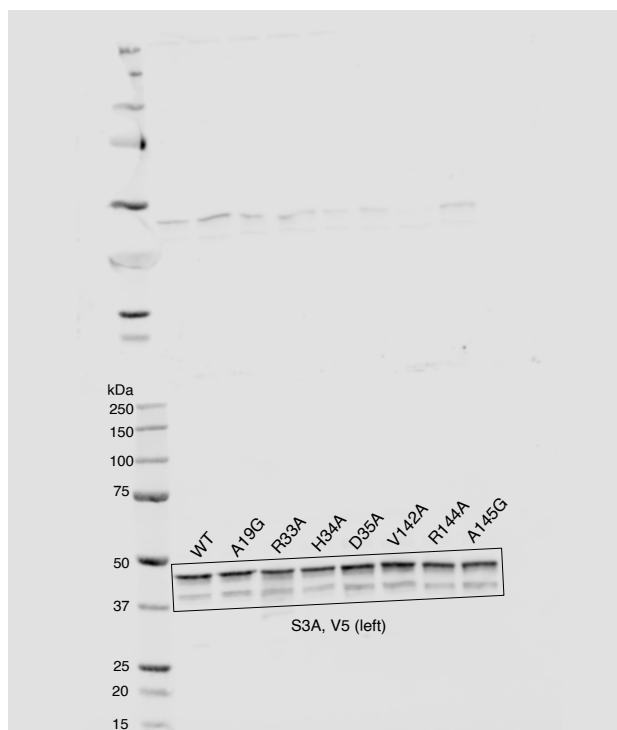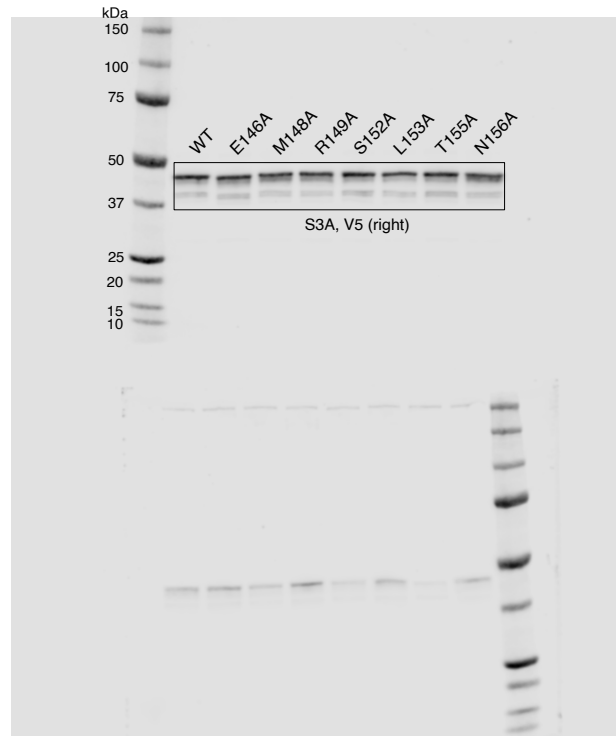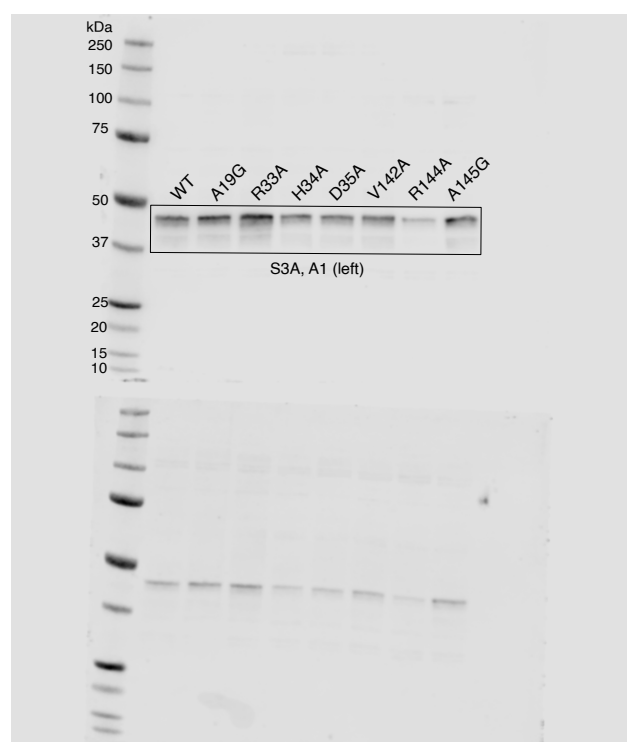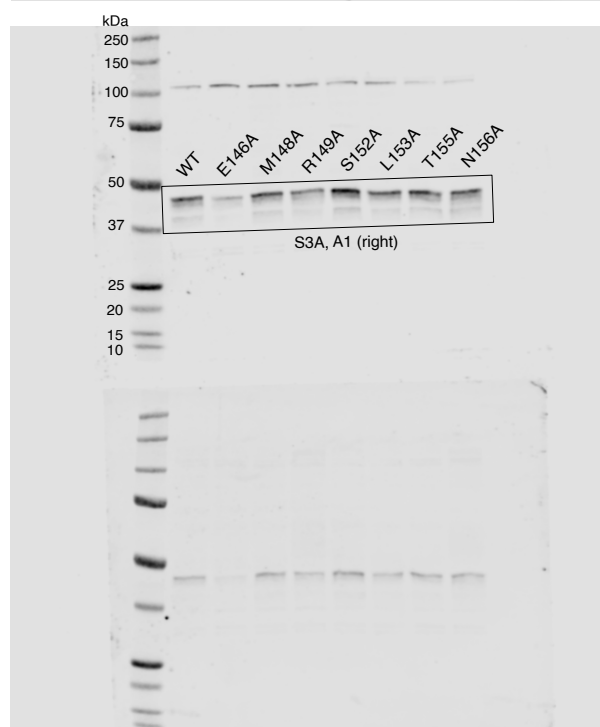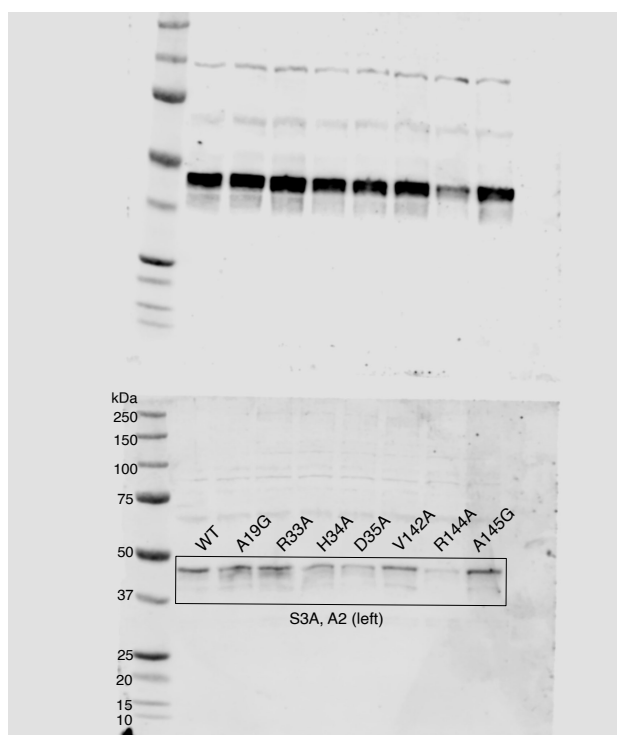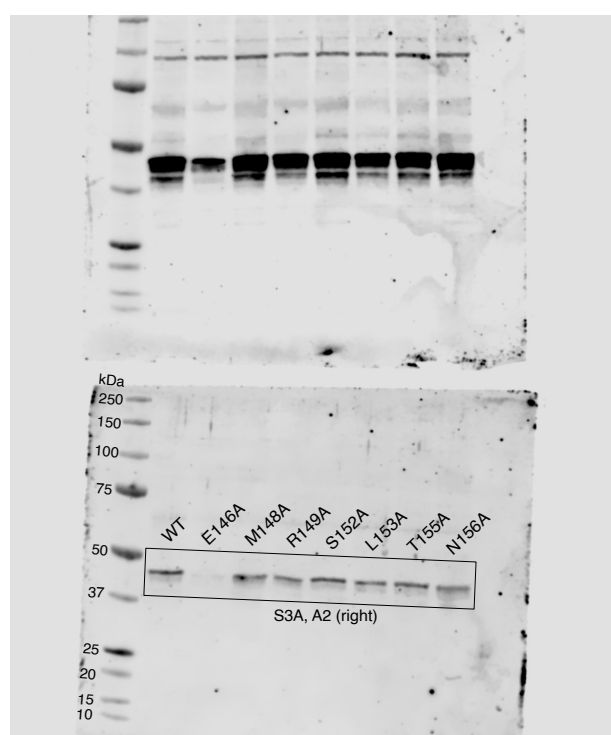

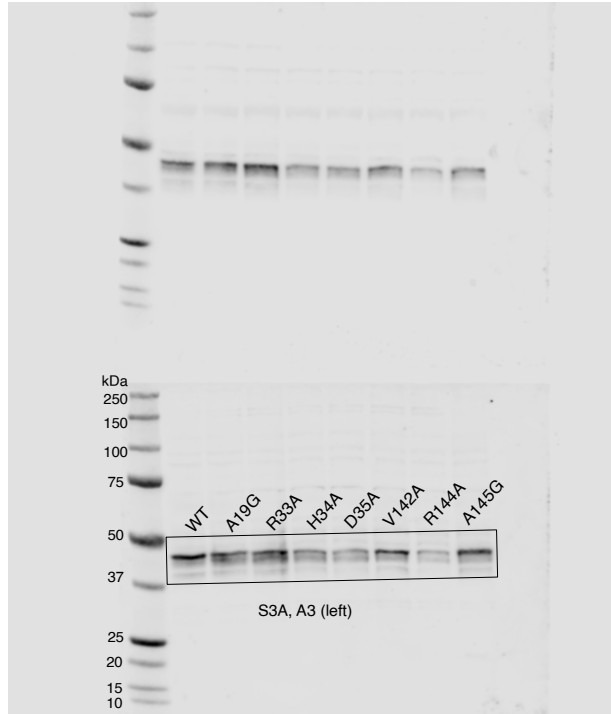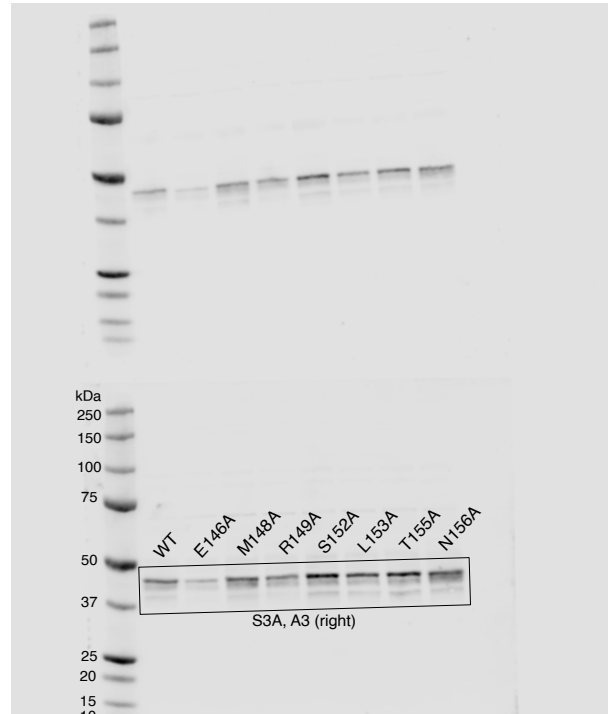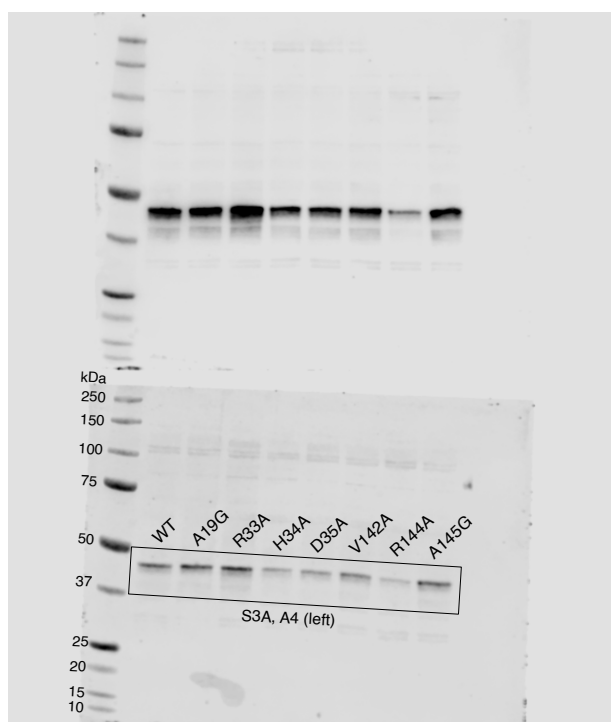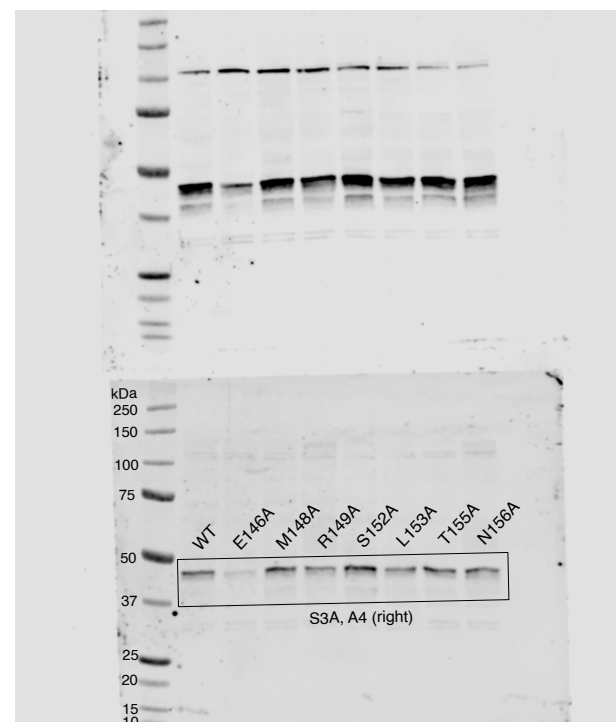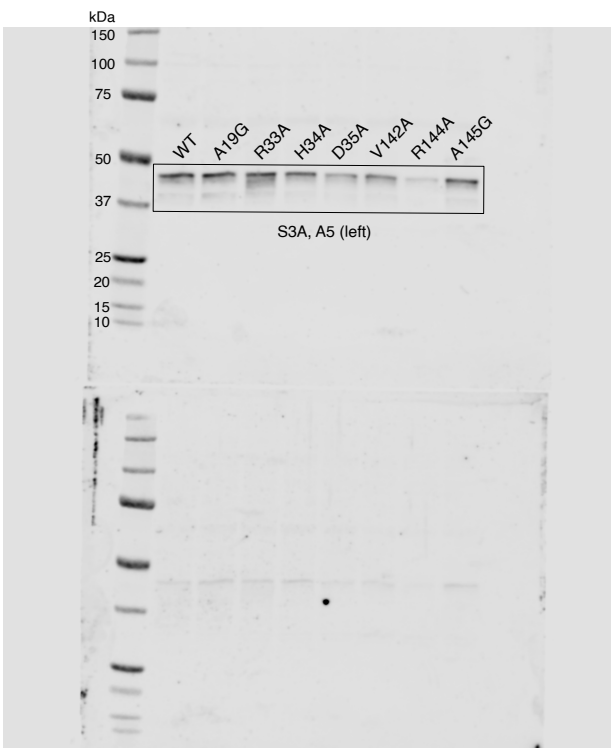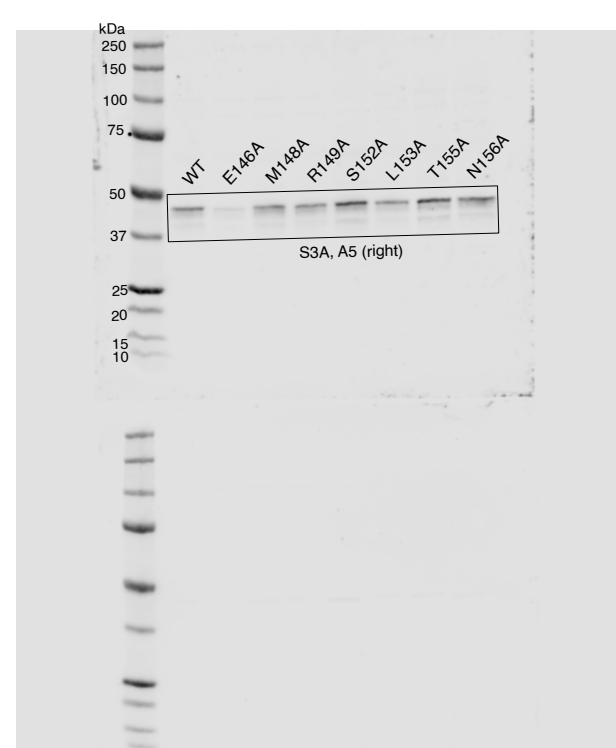

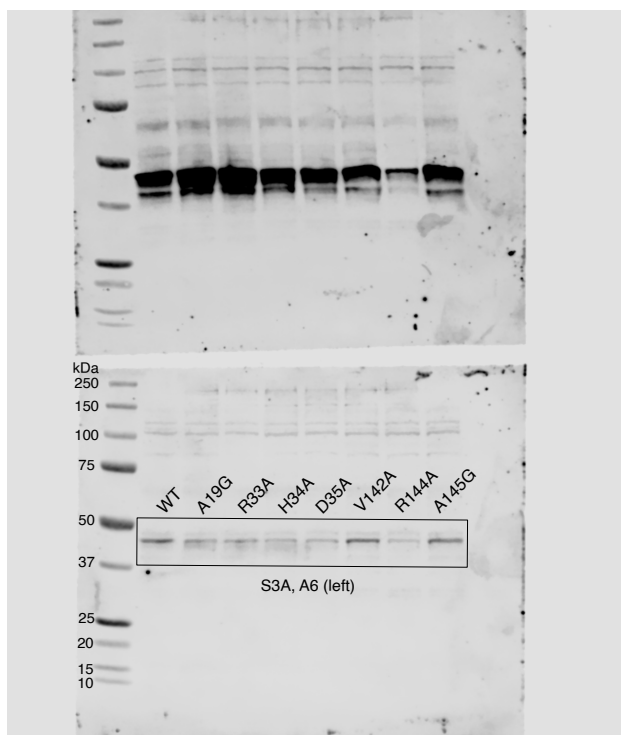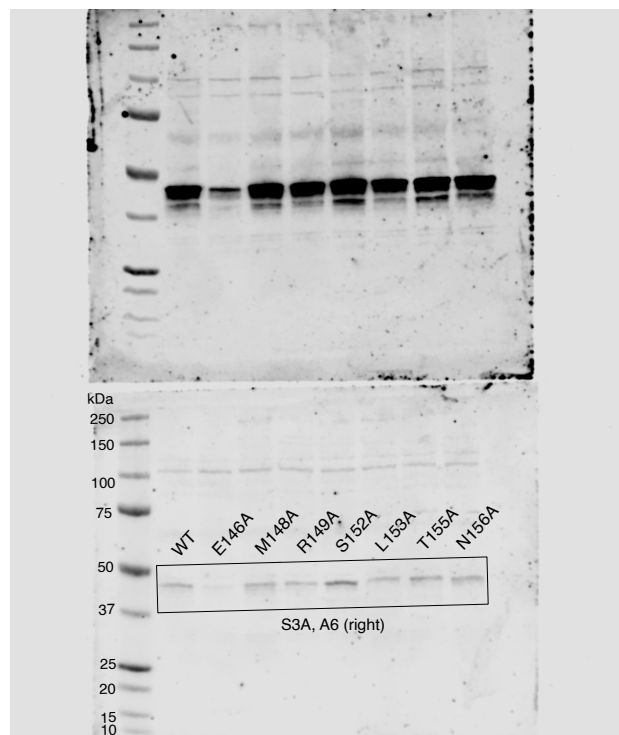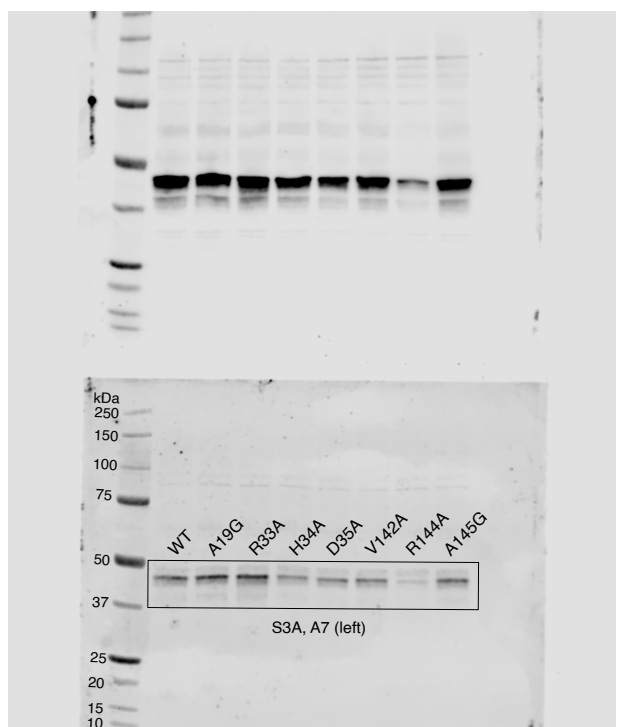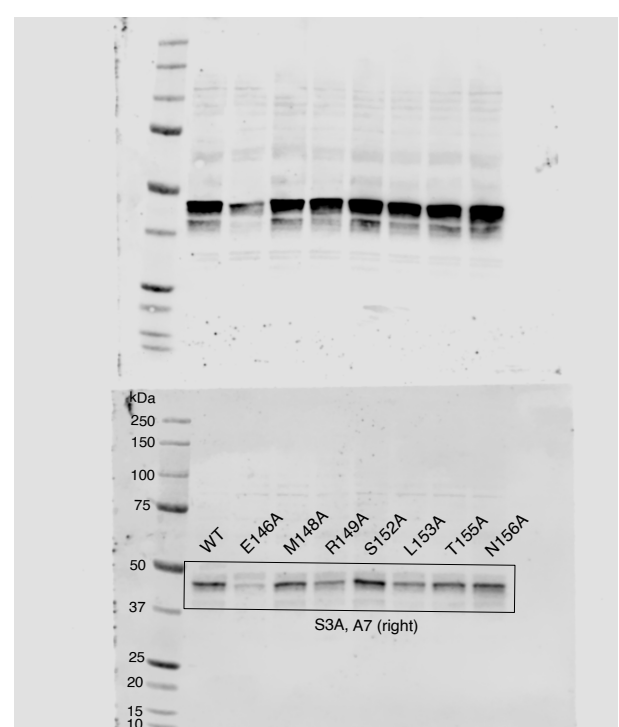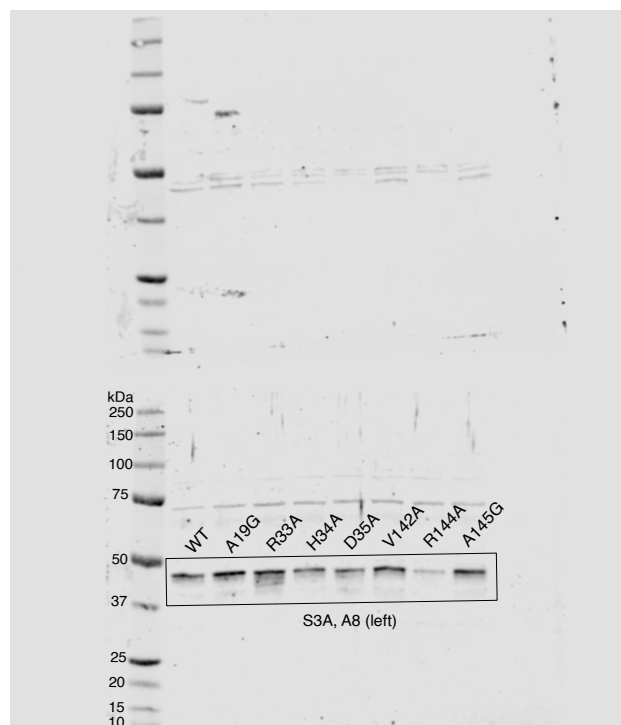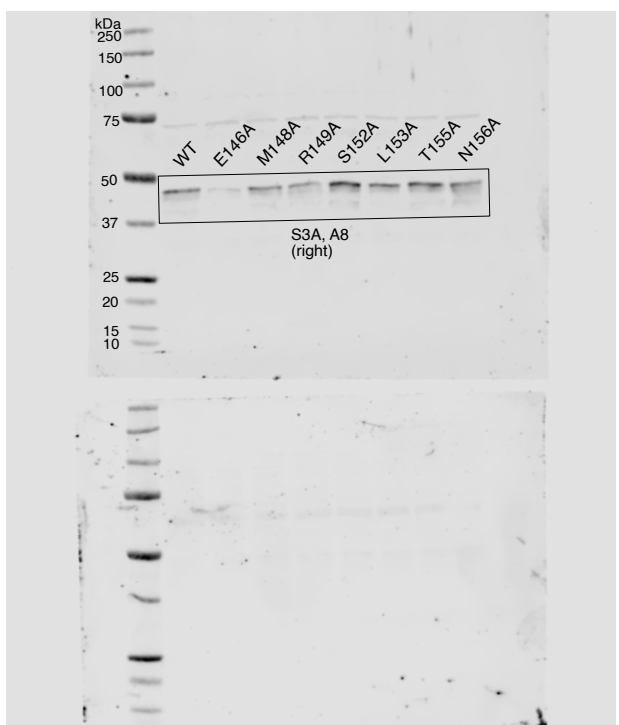

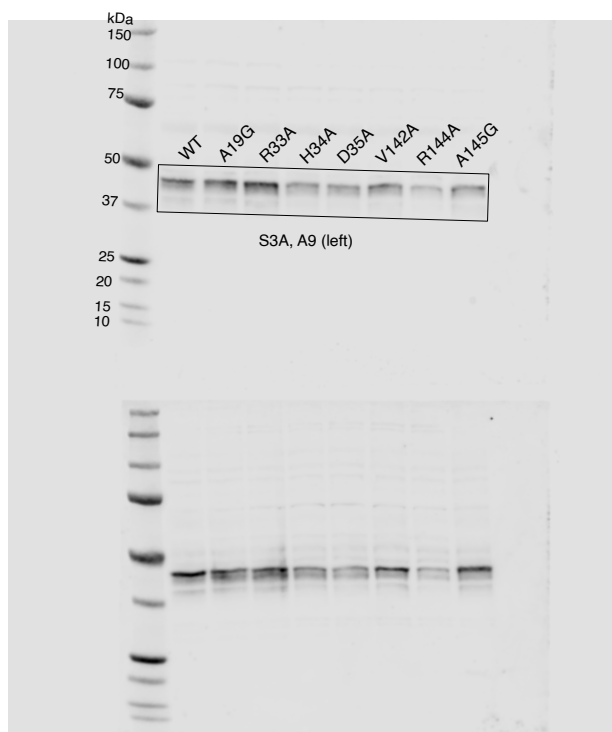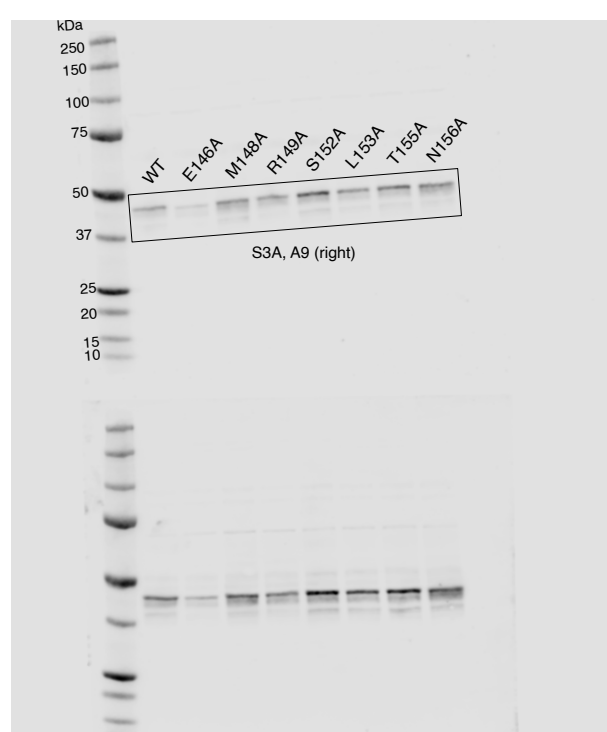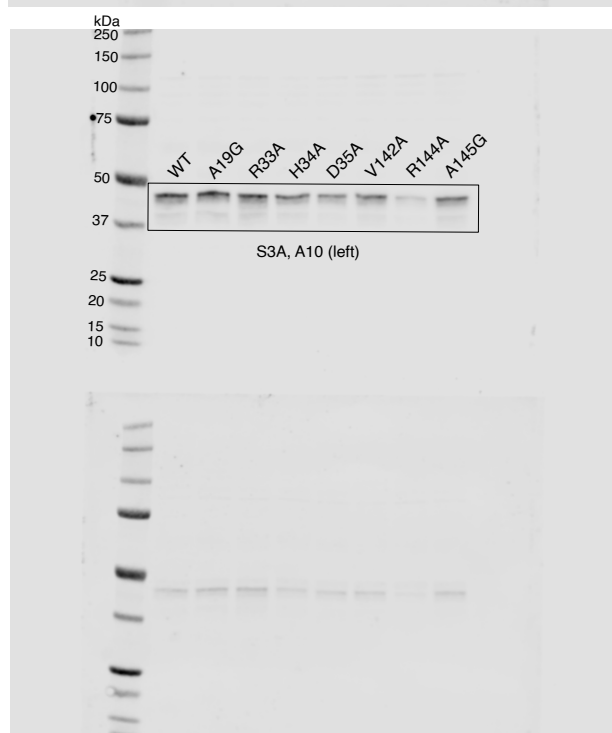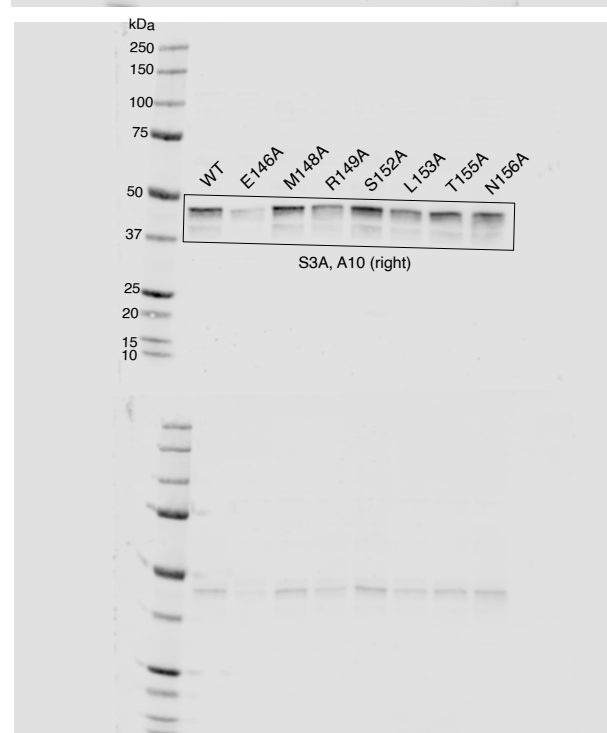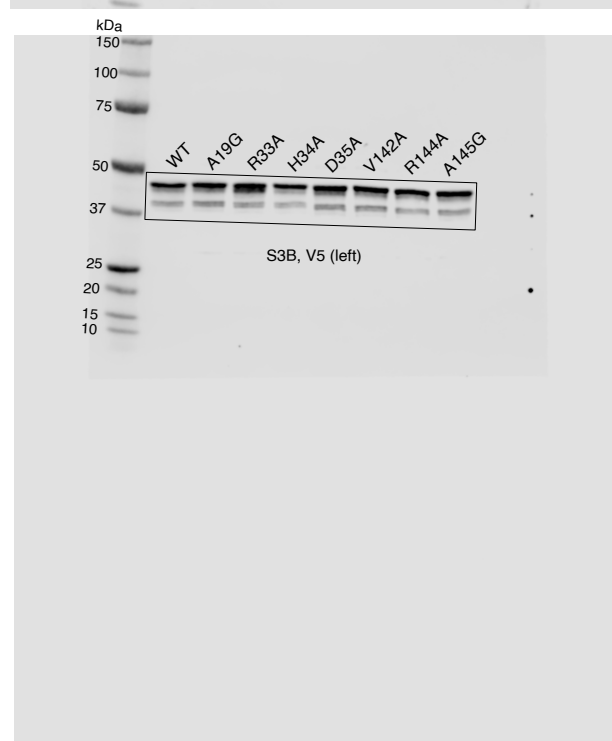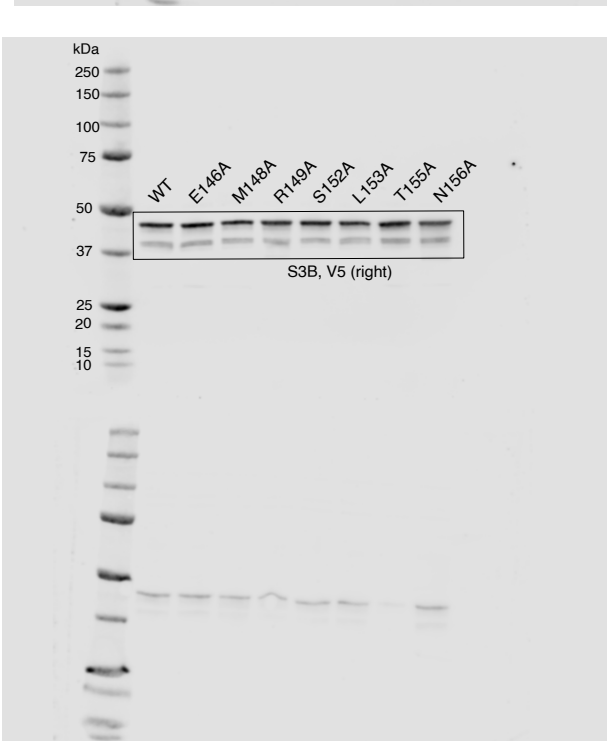

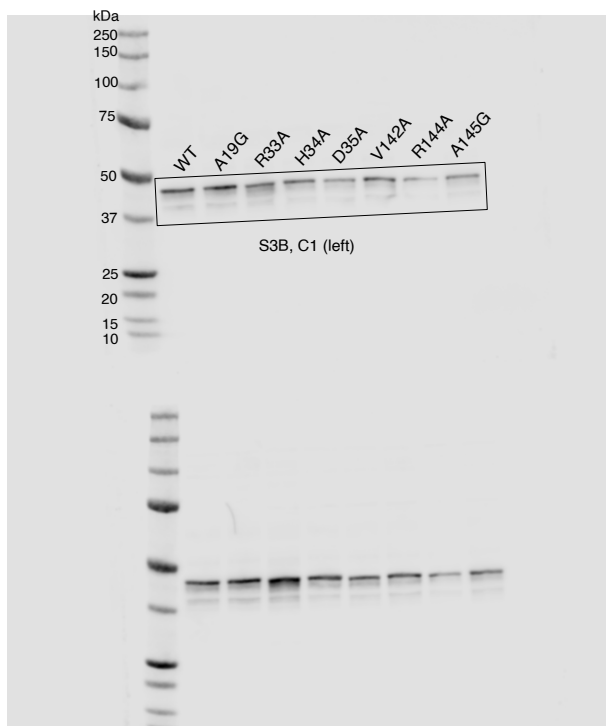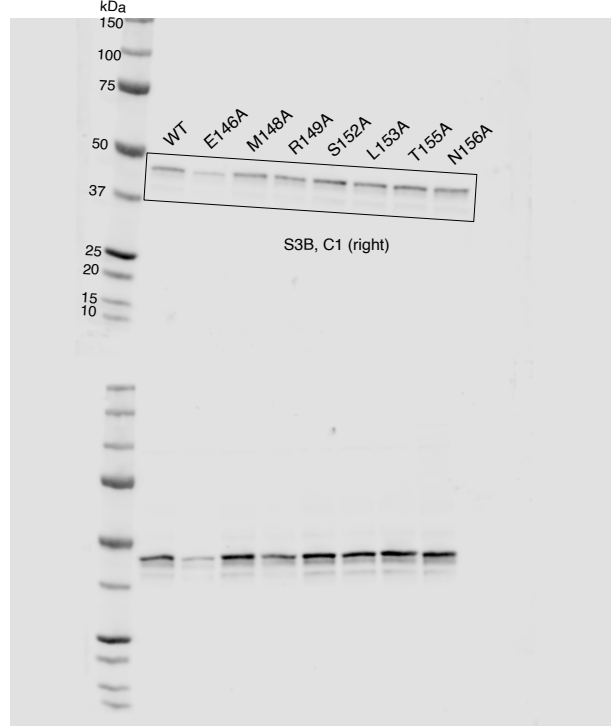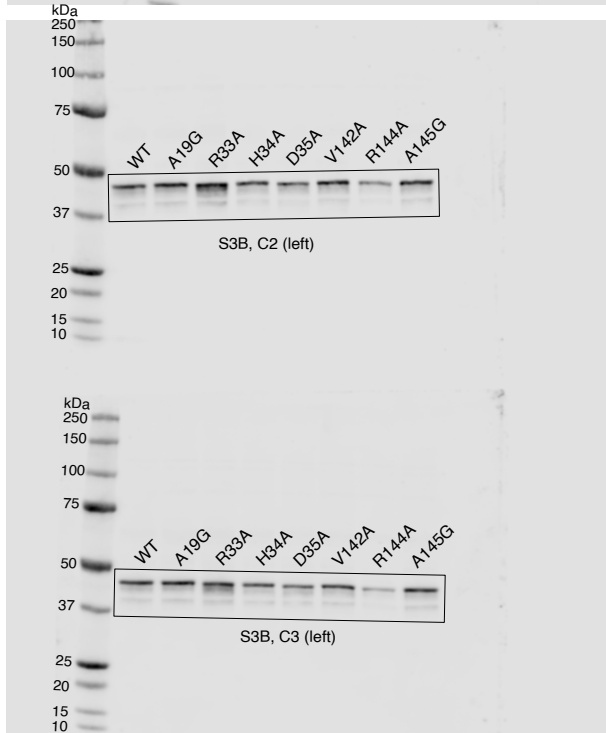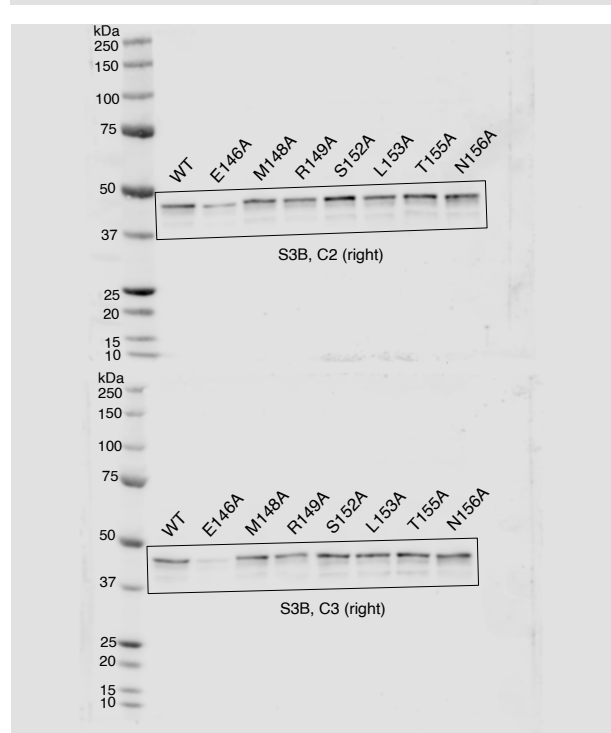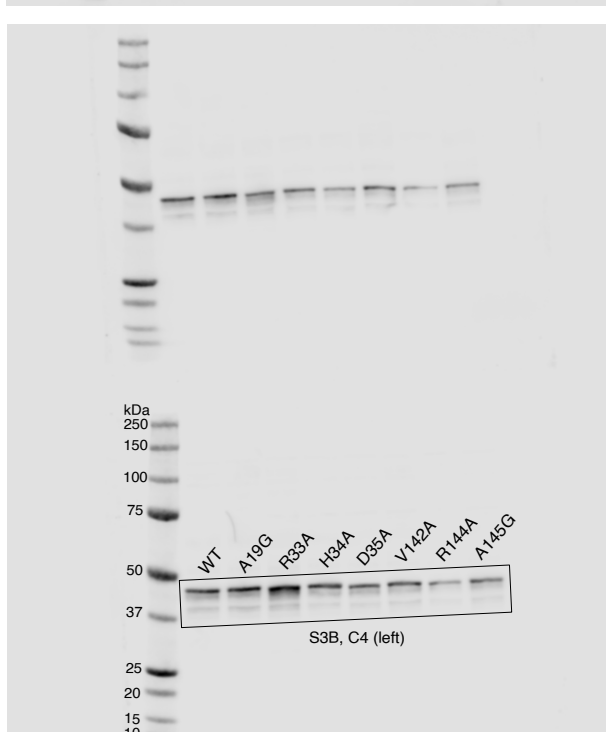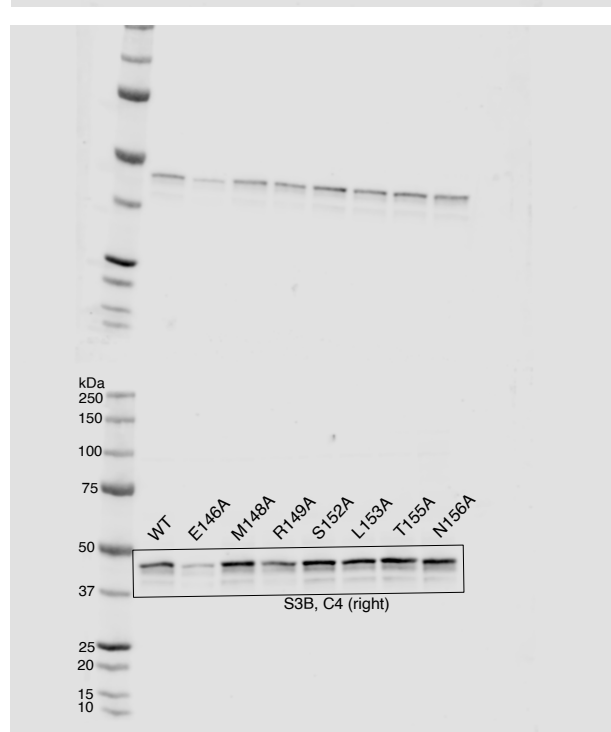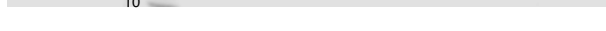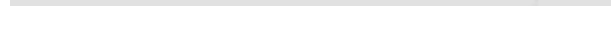

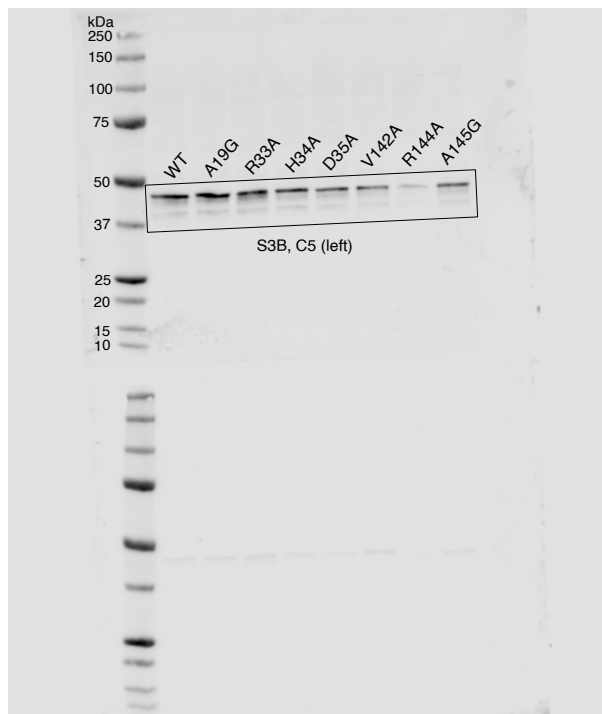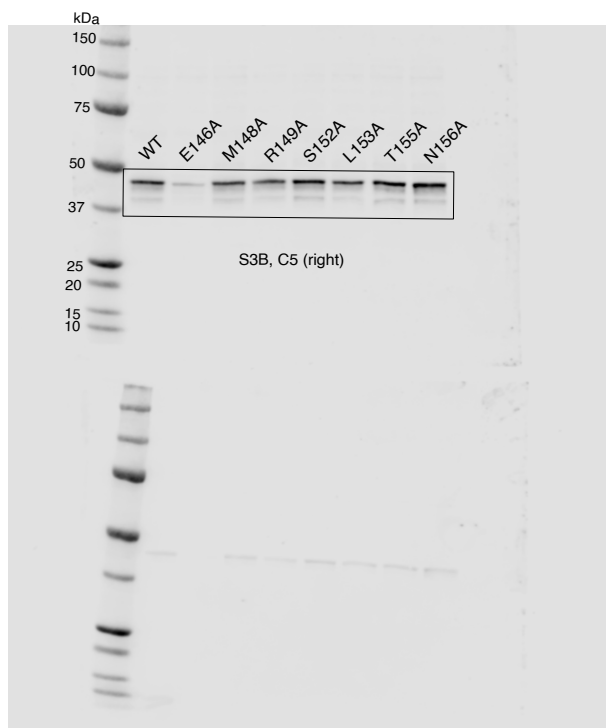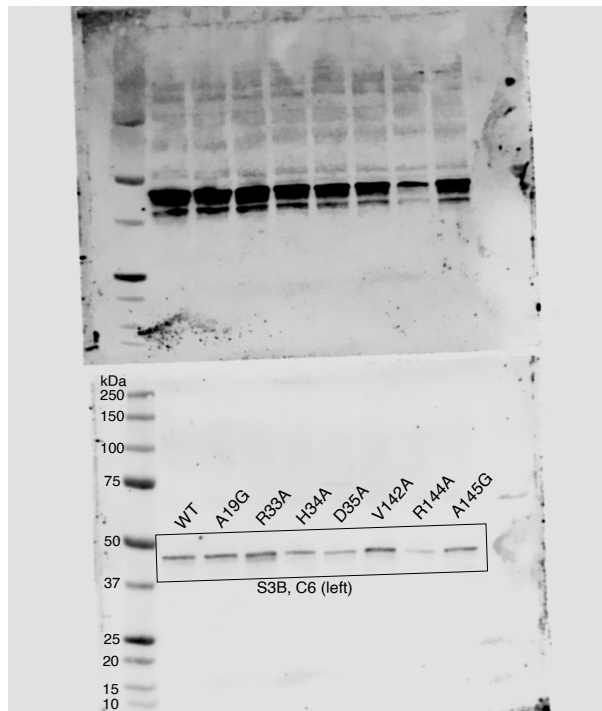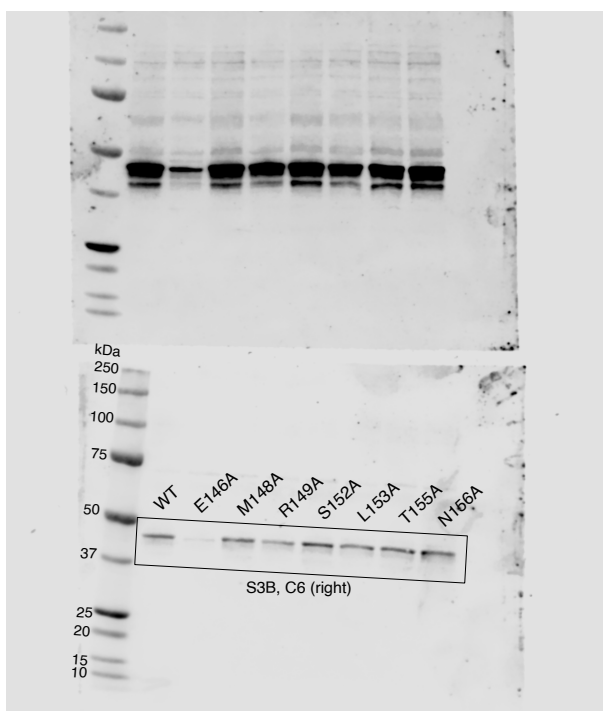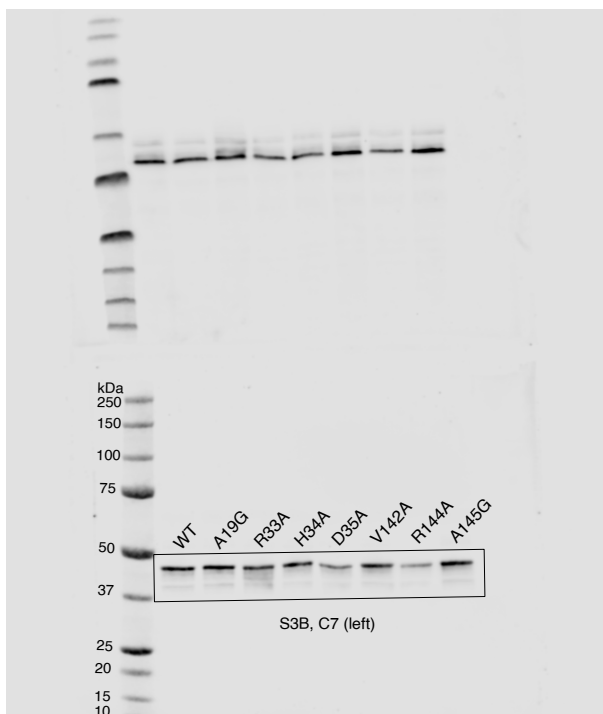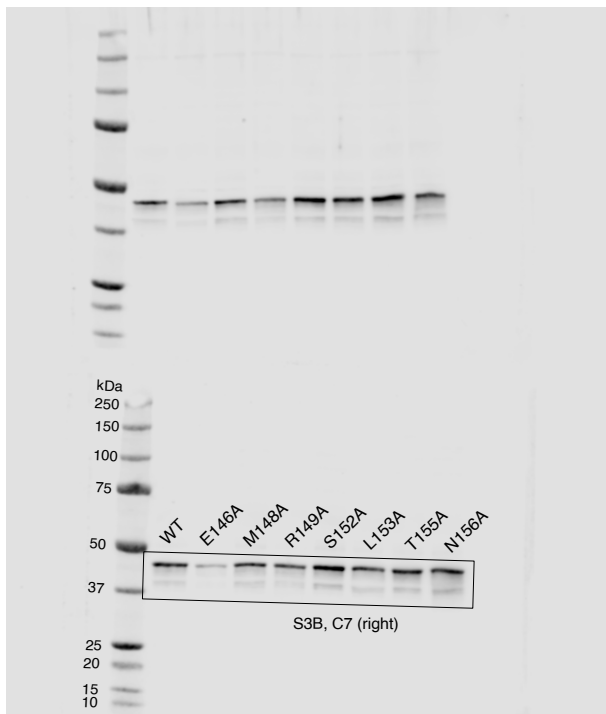

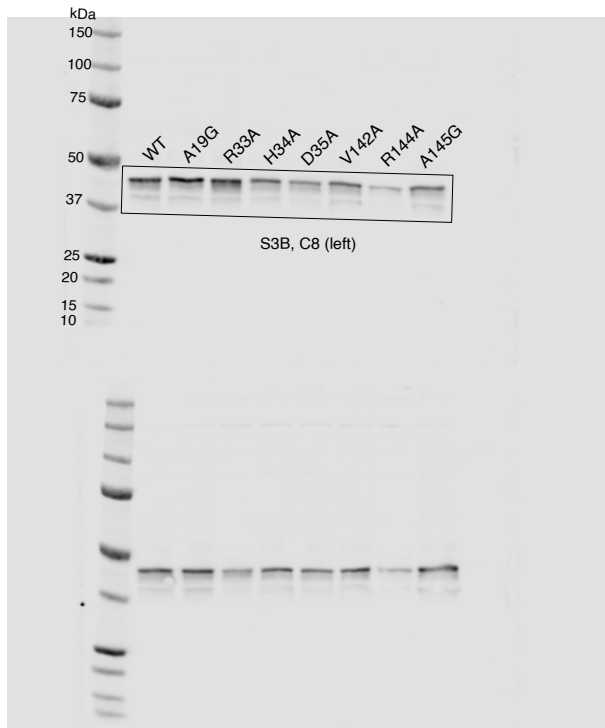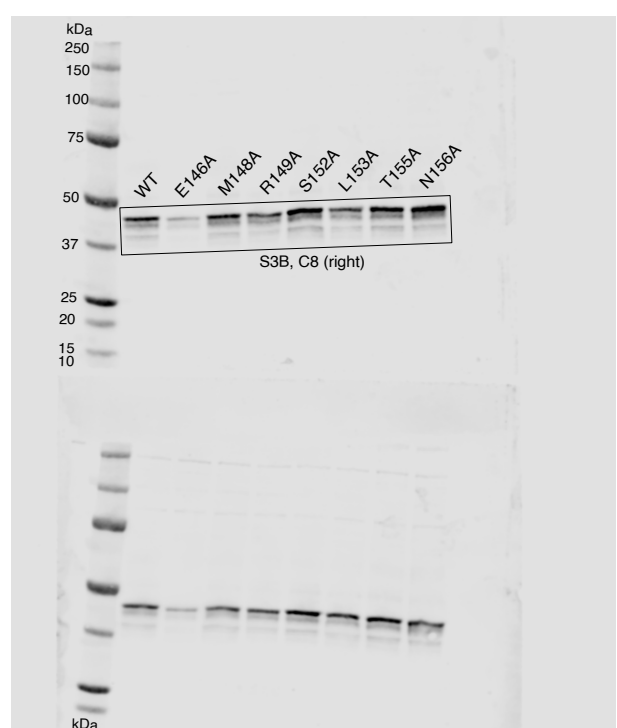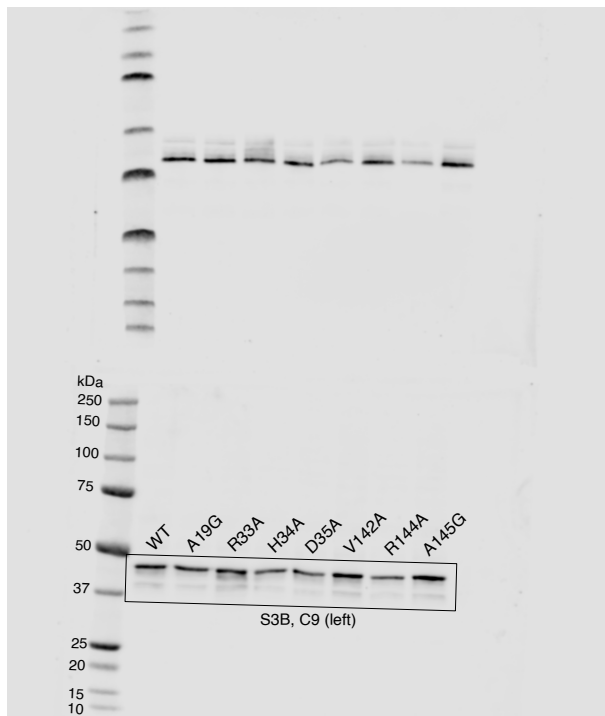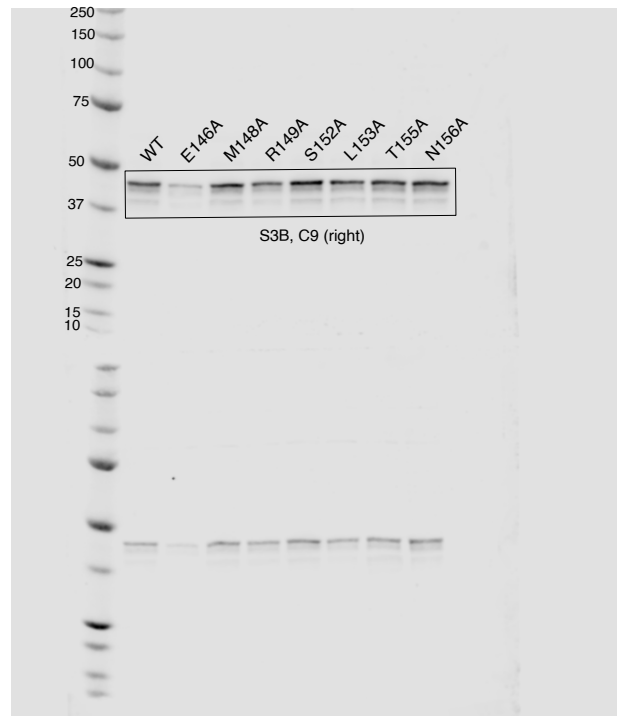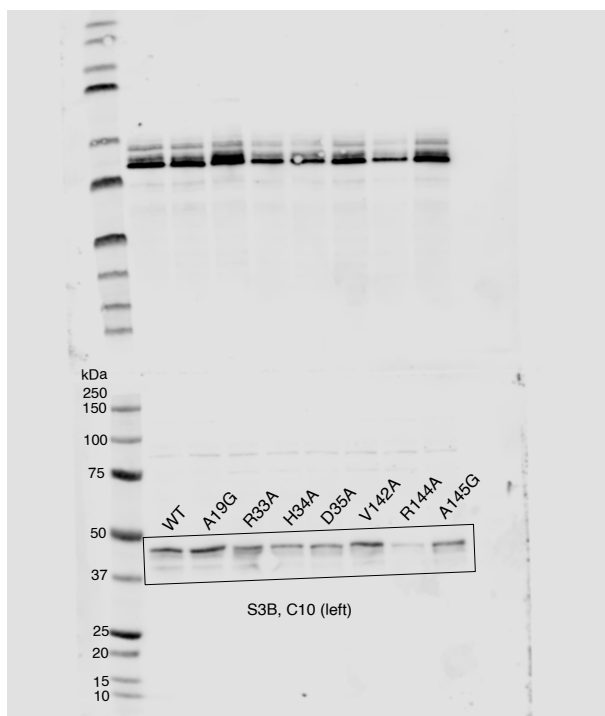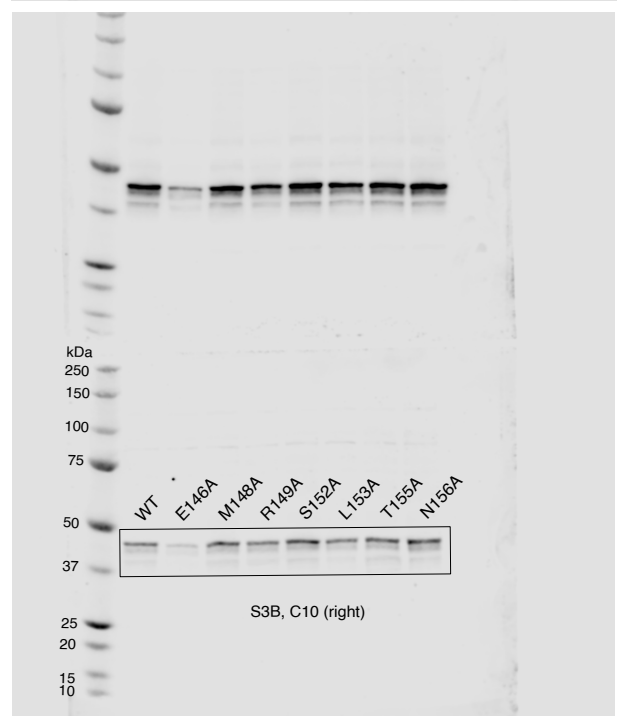

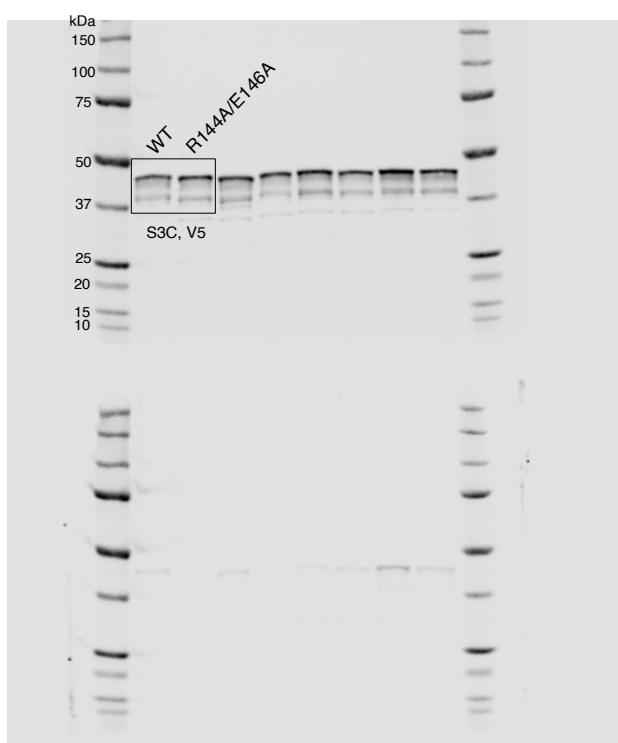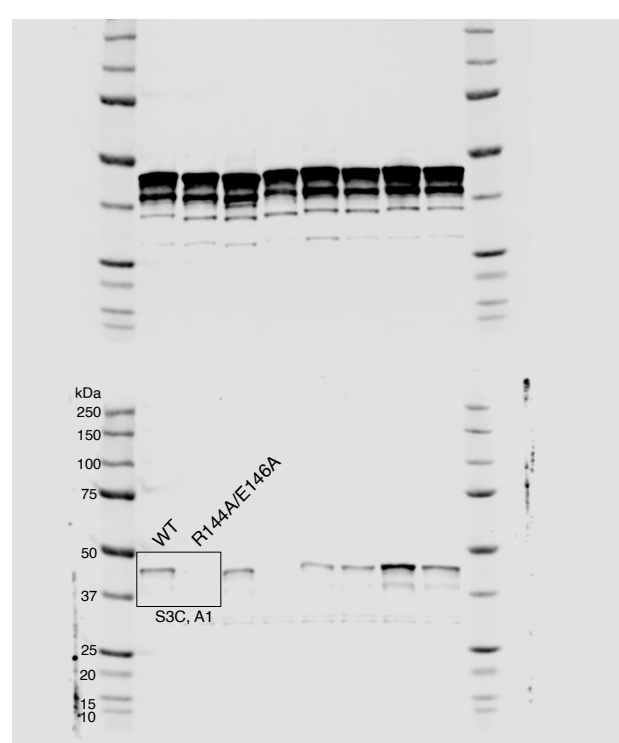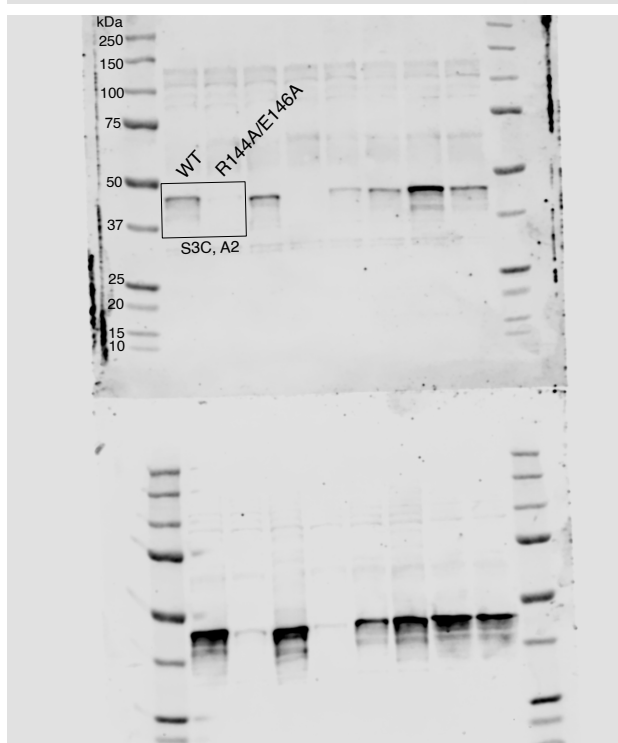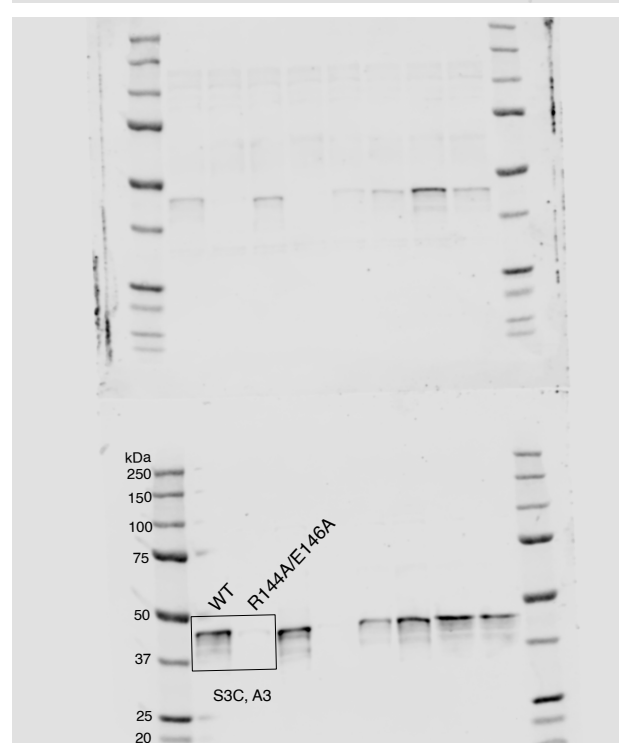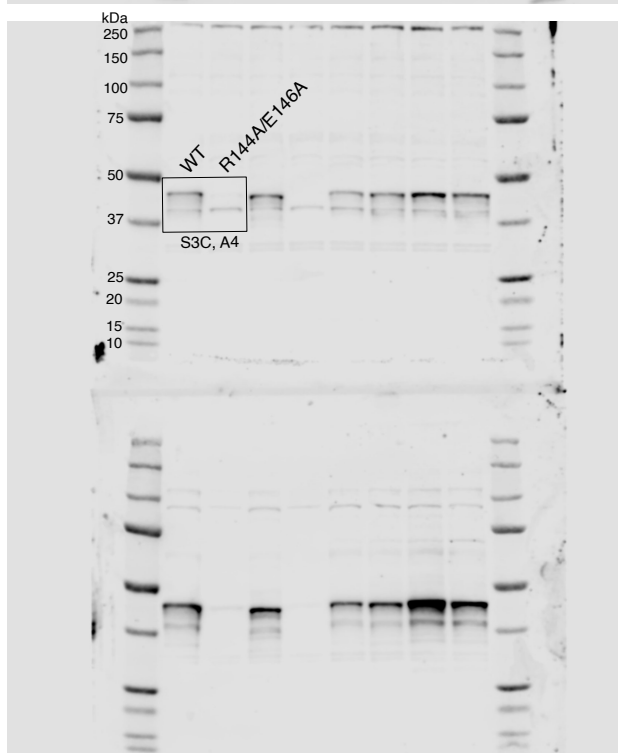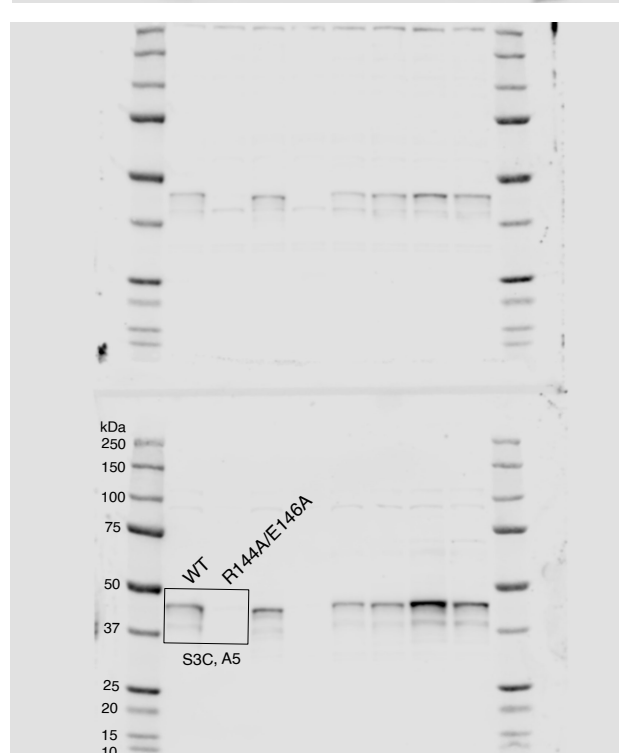

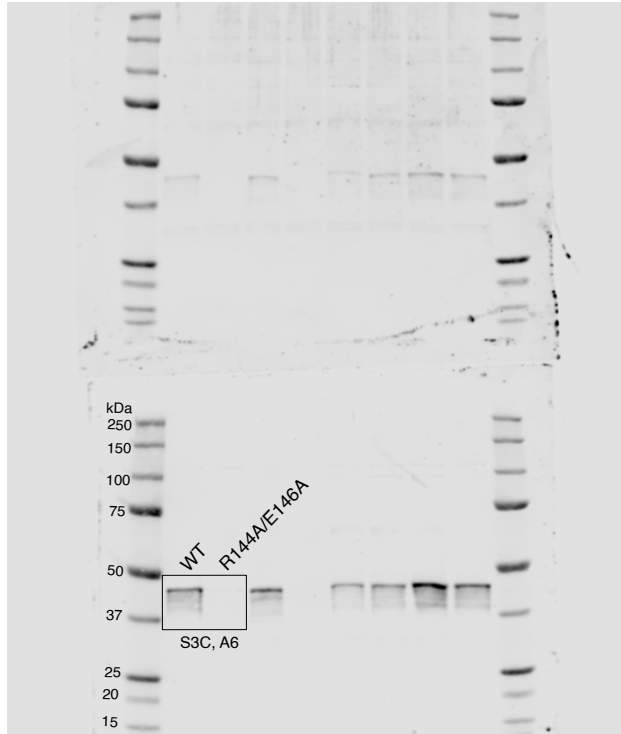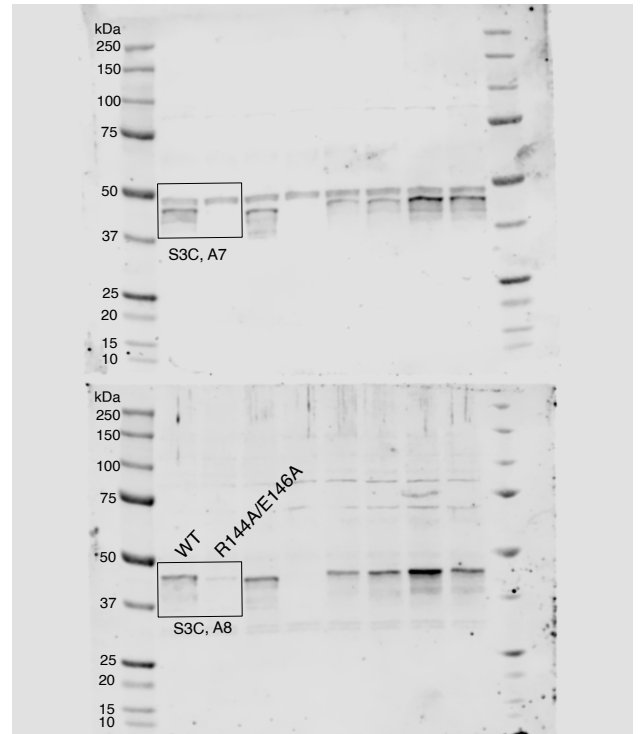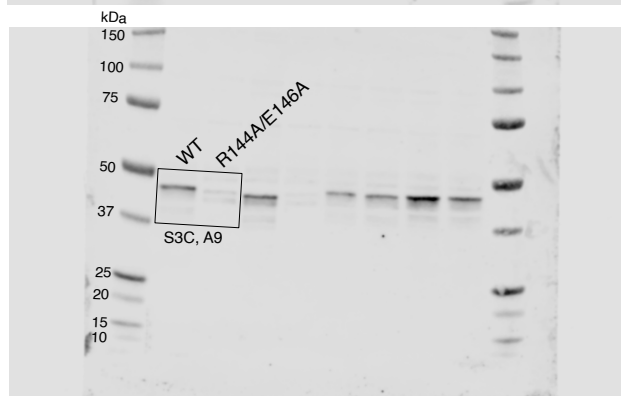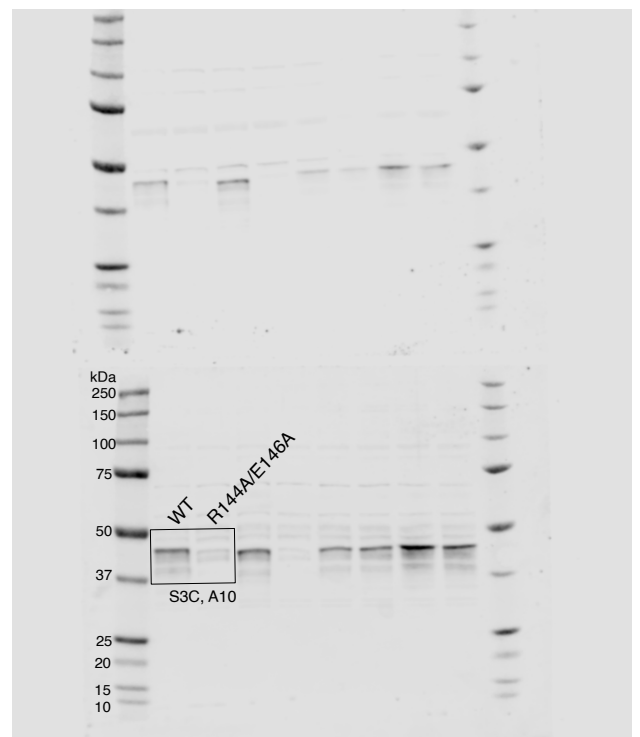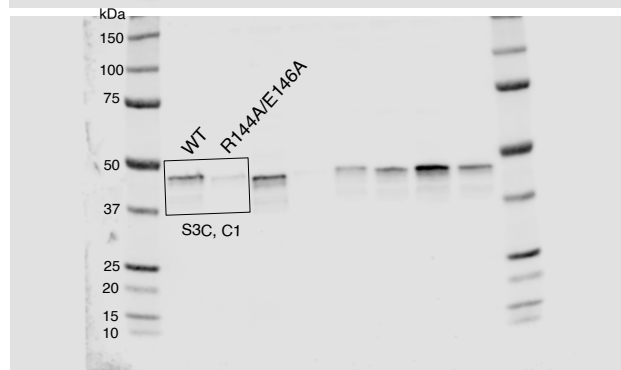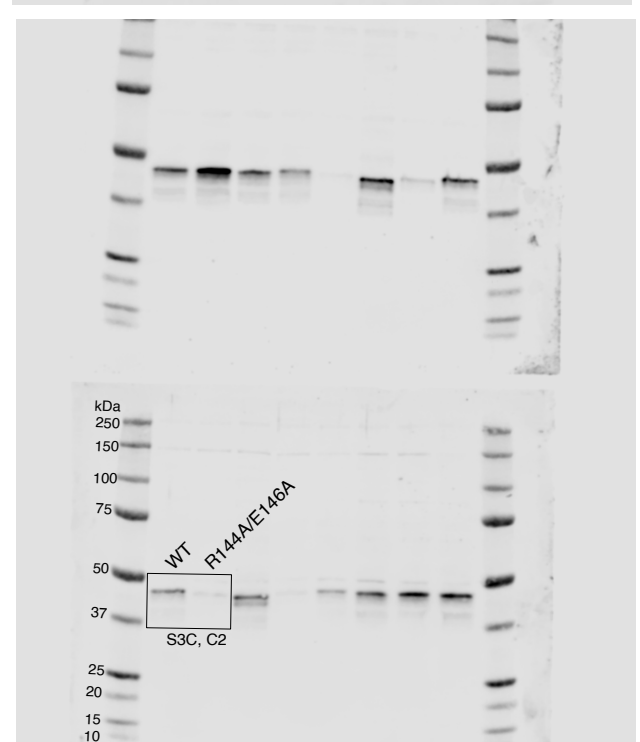

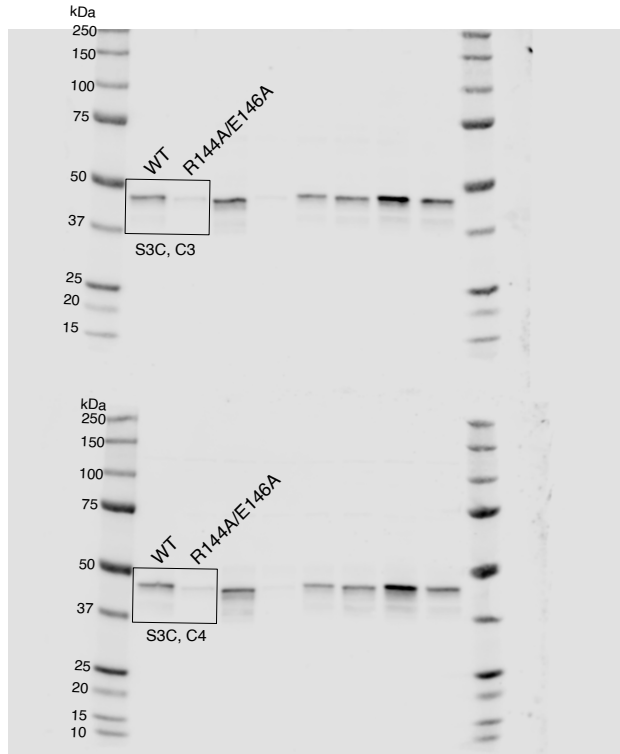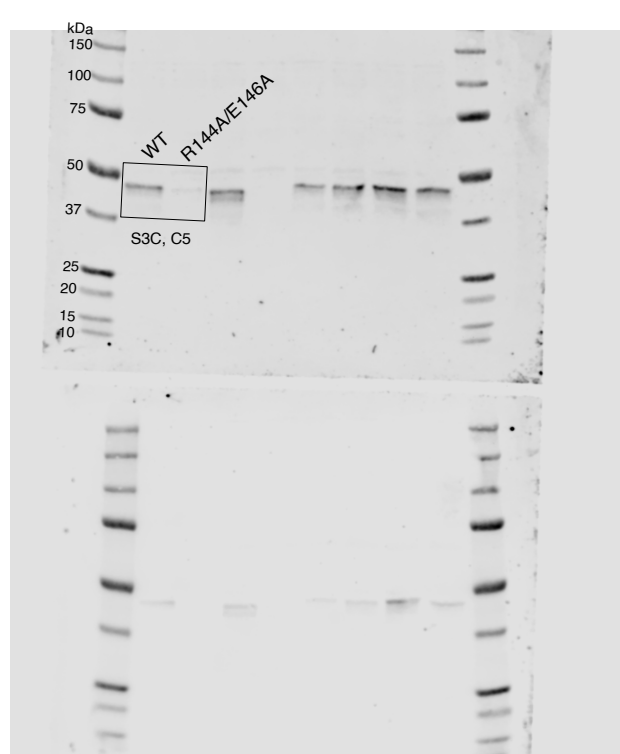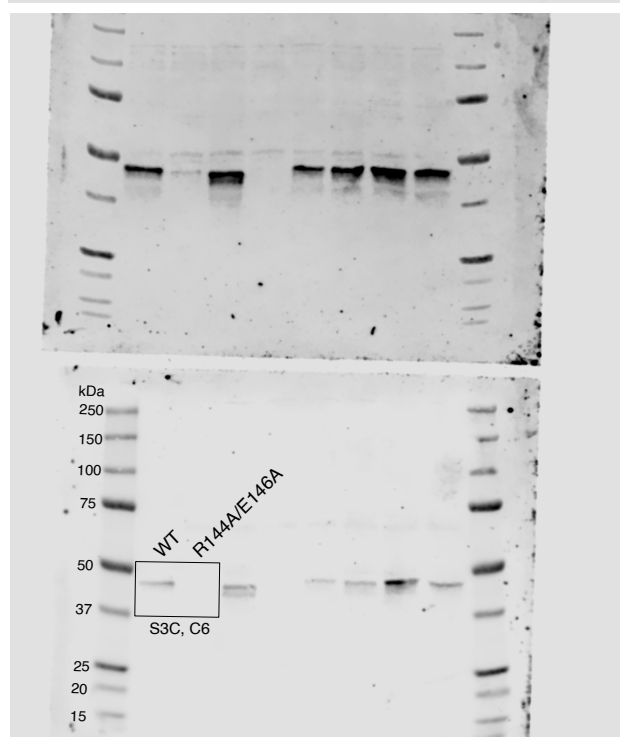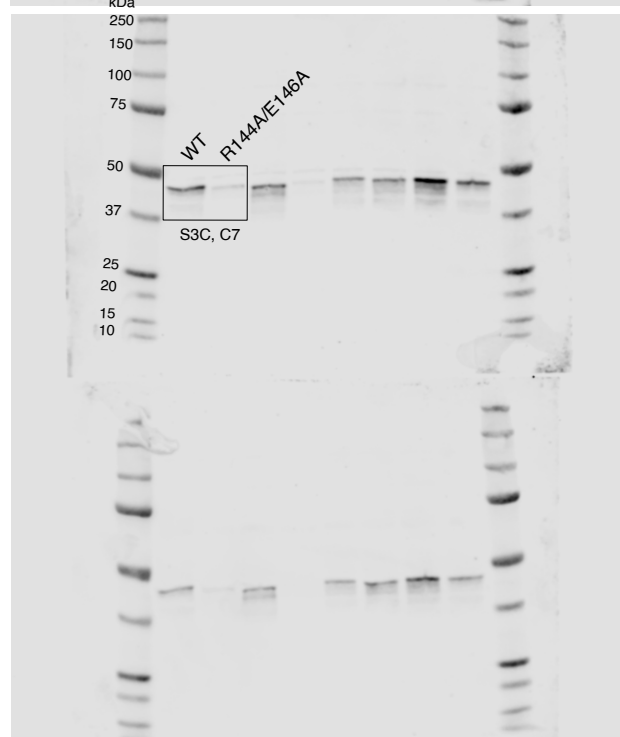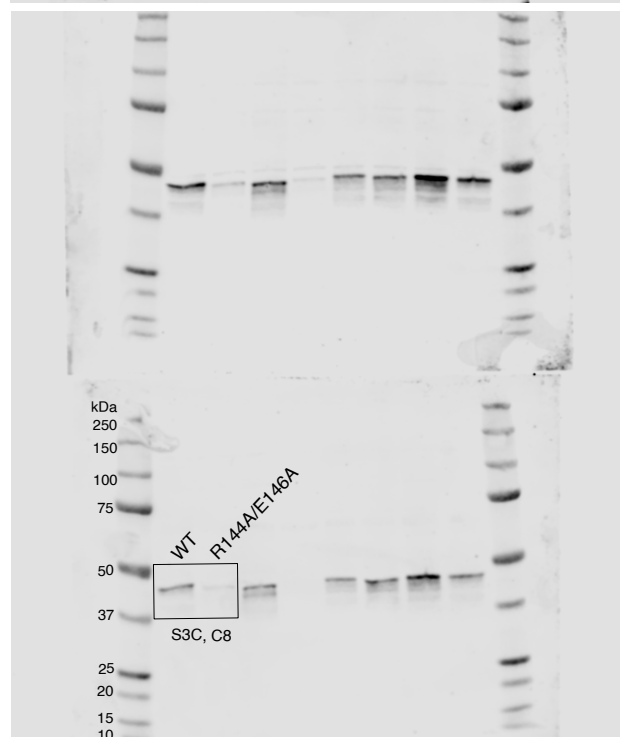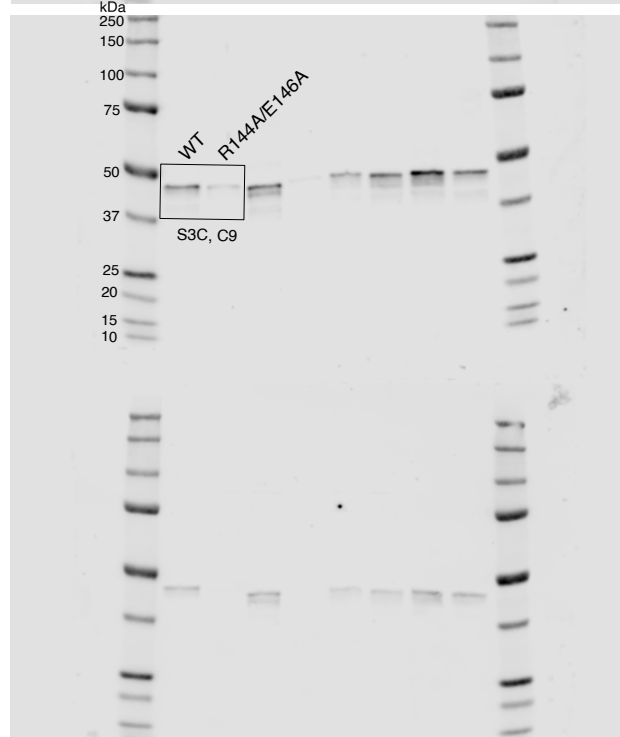

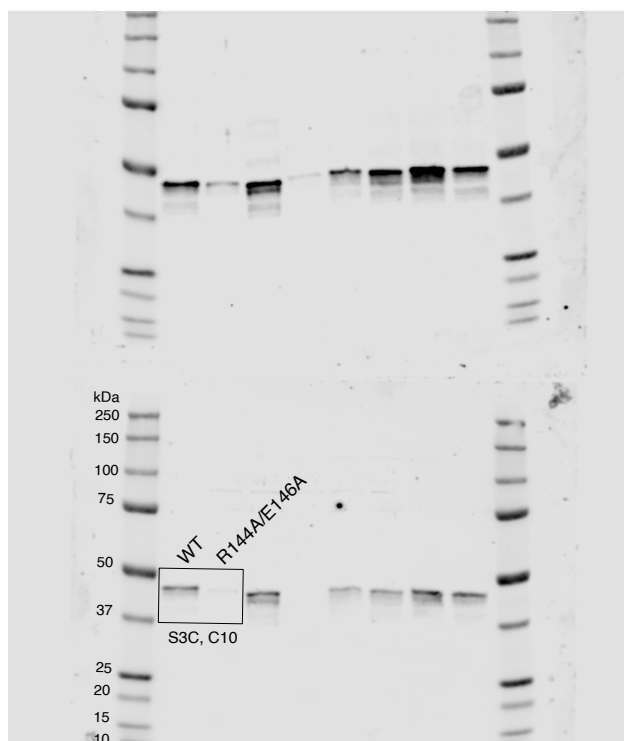

Supplement: SourceData FS3 — is the source file for Fig. S3. [file jem_20242039_sourcedatafs3.pdf]
